# Human wellbeing outcomes of involvement in industrial crop production: Evidence from sugarcane, oil palm and jatropha sites in Ghana

**DOI:** 10.1371/journal.pone.0215433

**Published:** 2019-04-25

**Authors:** Abubakari Ahmed, Eric Dompreh, Alexandros Gasparatos

**Affiliations:** 1 Department of Planning, Faculty of Planning and Land Management, University for Development Studies at Wa Campus, Wa, U.W.R, Ghana; 2 Graduate Program in Sustainability Science–Global Leadership Initiative (GPSS-GLI), The University of Tokyo, Building of Environmental Studies, Kashiwanoha, Kashiwa City, Chiba Japan; 3 Integrated Research System for Sustainability Science (IR3S), The University of Tokyo, Bunkyo-ku, Tokyo, Japan; University of British Columbia, CANADA

## Abstract

An extensive body of theoretical work has advocated the use of multiple human wellbeing indicators to assess the outcomes of agricultural investments in Sub-Sahara Africa (SSA). However, few studies have actually achieved it. This study investigates the human wellbeing outcomes of involvement in industrial crop production in Ghana by comparing the levels of different objective and subjective wellbeing measures for groups involved in industrial crop production as plantation workers and smallholders, and groups not involved (i.e. control groups). We use household income, adult consumption and the multidimensional poverty index (MPI) as indicators of objective wellbeing. We measure subjective wellbeing through self-reported levels of satisfaction with life, worthwhileness, happiness and anxiousness. Propensity Score Matching (PSM) analysis is used to assess whether involvement in industrial crop production increases household income and consumption. Overall, for most indicators of objective wellbeing industrial crop outgrowers, smallholders and independent smallholders are better off compared to other groups in their respective sites (in terms of mean scores), but involvement does not necessarily brings human wellbeing benefits (PSM analysis). On the other hand plantation workers are either worse off or have similar level of objective human wellbeing with control groups in their respective sites (in terms of mean scores), but involvement sometimes brings human wellbeing benefits (PSM analysis). However, workers tend to benefit from access to plantation infrastructure, which has a positive effect to their multi-dimensional poverty. In most cases the objective wellbeing measures do not correlate well with self-reported levels of subjective wellbeing. It is important to combine such indicators when evaluating the human wellbeing outcomes of agricultural investments in order to obtain a more comprehensive outlook of whether industrial crop production can become a valuable rural development strategy in SSA.

## Introduction

Industrial crop production has been expanding in several parts of Sub-Sahara Africa (SSA) during the past decades [1]. Perhaps the most widely studied industrial crop expansion occurred in the last decade, with the promotion of biofuel feedstocks such as jatropha [1]. Depending on the national context, this expansion has aimed to meet different policy objectives such as rural development, national economic growth and/or energy security [2,3]. The expectations of improved human wellbeing in rural areas through livelihoods diversification, poverty alleviation, and income/employment generation has often catalyzed national and local support for industrial crop production, and eventually the allocation of large tracts of land for such purposes [3,4]. Although jatropha was the most widely promoted biofuel-related industrial crops in SSA before its widespread collapse [4], other potential biofuel crops such as sugarcane and oil palm are currently being promoted across the continent for multiple industrial purposes [5–10].

However, the human wellbeing and rural development outcomes of engaging in the production of such crops depends on various factors such as the crop, mode of production (e.g. production in plantations or by smallholders), available markets, and the local socioeconomic and environmental context [11–14]. For example, apart from the absolute level of income obtained through engagement in industrial crop production, other factors such as the payment structure (e.g. one-off payments for smallholders, stable monthly salaries for plantation workers) can also affect significantly the rural development outcomes [15]. Furthermore, the different uses of industrial crops [e.g. bioenergy, food industry, other industrial uses) can further dictate their markets options, and eventually the costs and benefits to those involved in their production [16–18].

In any case, it is important to assess the household-level human wellbeing outcomes for the different possible types of involvement in industrial crop value chains (e.g. plantation workers, smallholders, outgrowers) in order to understand their possible rural development and poverty alleviation benefits in SSA [19]. However, our current understanding of such human wellbeing outcomes is fragmented. Firstly, most relevant studies tend to focus on single indicators of human wellbeing such as income, consumption or poverty [20,21]. While some studies have recently adopted measures of multidimensional poverty in industrial crop contexts [22], to our best knowledge no studies have combined mono-dimensional human wellbeing measures (e.g. income), with multidimensional poverty measures. Secondly, most current studies in agrarian contexts of SSA employ objective wellbeing measures, rather than measures of subjective wellbeing (e.g. satisfaction with life, happiness) [23–26]. While it is important to combine measures of objective and subjective wellbeing [27–31], there are very few studies that have jointly used and contrasted them in agrarian contexts of SSA [25,26].

The aim of this study is to undertake a comprehensive assessment of the objective and subjective human wellbeing outcomes of involvement in the production of different industrial crops (under various production models) in Ghana. We use multiple subjective and objective measures to gain a comprehensive understanding of how involvement in such crop systems can affect the human wellbeing at the household level. We focus on crops that can potentially be used as biofuel feedstocks (i.e. oil palm, sugarcane, jatropha), as such crops have experienced a substantial expansion in the country. Jatropha was the most promoted industrial crop in Ghana [4] before its collapse [32,33], while there are current discussions to expand oil palm and sugarcane production partly for biofuel purposes [34]. Considering that rural development has been a major driver of industrial crop expansion in SSA [2] and Ghana in particular [32] (including for biofuel purposes), it is important to understand the actual human wellbeing outcomes of involvement in the production of these crops. This can help ascertain whether industrial crop promotion is a good rural development strategy for Ghana (and possibly other SSA contexts).

To achieve this, we compare households involved in industrial crop production (i.e. waged plantation workers, smallholders) with households not involved (i.e. control groups). We focus on three operational industrial crop projects in Ghana, namely sugarcane (smallholder-based production), jatropha (plantation-based production) and oil palm (hybrid system that combines production in a large plantation surrounded by smallholders). We employ various indicators of objective and subjective wellbeing. In particular, we use income, consumption and the multidimensional poverty index as measures of objective wellbeing [22,35–38], and happiness, satisfaction with life, worthwhileness, and anxiousness as measures of subjective wellbeing. By juxtaposing so diverse metrics, crops, and modes of production, we seek to provide a more comprehensive picture of how involvement in industrial crop value chains affects the wellbeing of rural households. Being conscious of the socioeconomic, environmental and agronomic differences between crops and areas of production, we primarily compare the human wellbeing outcomes for groups within the same site. However, we also identify and discuss some of the consistent patterns that emerge between sites.

First we outline the adopted methodology, including the study sites, and the data collection and analysis methods (see “Methodology”). Subsequently we report the findings for the different indicators in each site, as well as their correlations (see “Results”. Finally we elicit the main patterns across the study groups and sites, and identify some of the policy implications and caveats of this study (see “Discussion”).

## Methodology

### Study sites

**Fig 1:**
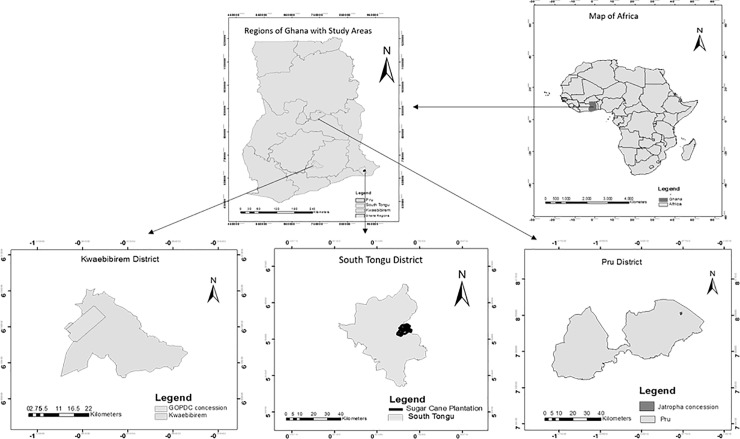
Location of the three study sites in Ghana.

The selected study sites have very different characteristics. First, they represent the main different biofuel feedstock options in Ghana (i.e. sugarcane, jatropha, oil palm) [32]. Between them, the study projects reflect the main modes of industrial crop production, i.e. smallholder-based, large plantations and hybrid systems [2] (Table 1). Table 1 is compiled based on [39,40]. They are also situated within different agro-ecological zones of Ghana that have radically different climatic and ecological conditions (Fig 1).

**Table 1:** Key characteristics of the study sites.

Feature	Dabala (sugarcane)	Yeji (jatropha)	Kwae (oil palm)
Location	5°59’7.76”N 0°40’29.76”E	8°13’34.46”N 0°39’12.93”W	6°14’40.82”N 0°58’12.43”W
District	South Tongu	Pru	Kwaebibirem
Industrial crop company	-	Smart Oil	GOPDC
Year of industrial crop production	Uncertain	2006	1975
Land ownership	Individual family farms	Corporate plantation	Corporate plantation surrounded individual family farms
Mode of production	Smallholder-based	Plantation-based	Hybrid
Agro-ecological zone	Semi-deciduous forest	Savanna	Rainforest
Agricultural water use	Rainfed, with irrigation during the dry season	Rainfed	Rainfed
Land acquired (ha)	4,124	6,750	14,000
Area cultivated (ha)	2,450	720	8,200
Annual Rainfall (mm)	900–1,400	1,088–1,197	1,400–1,800
Poverty incidence (%)	25.4	43.1	16.6
Number of Poor Persons	21,957	54,818	18,457
Area code	DB	YJ	KW

The sugarcane study site is located in Dabala (South Tongu district) within the semi-deciduous forest ecological zone. This is the oldest and less formalized of the three projects, as sugarcane production is completely performed by individual smallholders, without the overseeing of a company, NGO, or government agency. Oral history suggests that sugarcane has been the main source of livelihoods in the area since the 1950s. The landscape is characterized by a floodplain with small rivers, which makes it very suitable for sugarcane farming. The sugarcane production zone falls within the larger Anlo-Keta Lagoon Complex which, was recognized in 1992 as a wetland of international importance by the Convention on Wetlands (Ramsar Convention) [39]. This multi-purpose landscape also comprises of a zone demarcated for the protection and conservation of the endangered Sitatunga swamp-dwelling antelope (*Tragelaphus spekii*) under the International Union for Conservation of Nature (IUCN) Red List. In 2012, with the intention to protect the landscape and the Sitaunga species, the 15 local communities located within the catchment area formed the Avu Lagoon Community Protected Area [39].

In Dabala, sugarcane is produced by independent smallholders and processed locally mainly for alcohol production. Individual smallholders acquire land for sugarcane production from local landowners and chiefs, often by establishing a benefit-sharing arrangement (based on an agreed sharing ratio) for the produced sugarcane. The sharing ratio and other forms of land payment/compensation are subject to the different prevailing local social norms and rules. The district within which Dabala is located has a relatively high poverty incidence rate (25.4%), ranking 118^th^ among the 216 districts of Ghana [40].

The jatropha study site is located in Yeji (Pru district). It contains a large mono-cultural plantation owned and operated by Smart Oil Ghana. The company was incorporated in 2006 and started the commercial cultivation of jatropha in 2011. The land concession (6,750 ha) was created through the consolidation of land parcels from the Kadue, Agentriwa, Kwaese communities. Currently, as there is no jatropha biodiesel production in Ghana [32], the produced jatropha seeds are exported to the European Union and other African markets such as Burkina Faso. Company employees mainly come from the surrounding communities including Kadue, Kobre, Kwaese, Agentriwa, and Kojo Boffour, and are paid wages on a daily and monthly basis. Most permanent employees are involved in agricultural activities (e.g. jatropha harvesters, nursery workers) with far fewer being engaged in managerial and other professional jobs (e.g. machinery operators). Apart from its permanent employees, the plantation also hires seasonal workers to pick jatropha seeds during the harvesting period, as well as for transplanting trees from the nursery to the plantation. The Pru district is characterized by high poverty (43.1%), making it the 50^th^ poorest district in Ghana (out of 216 districts) [40].

The oil palm site is located in Kwae (Kwaebibirem district). Oil palm production in characterized by a hybrid system, with production undertaken by a large company (Ghana Oil Palm Plantation Development Company, GOPDC) surrounded by individual outgrowers and independent smallholders. The GOPDC used to be a state-owned company until it was privatized in 1992 following the government’s mass divestiture programme during the 1990s. Currently, GOPDC is solely owned by the SIAT Group, a Belgian agro-industrial group. The main plantation covers over 8,200 ha, and is surrounded by approximately 10,000 ha of oil palm outgrowers and independent smallholders. The GOPDC directly supports about 7,200 outgrowers and requires them sell their oil palm harvest solely to the GOPDC mill located within the Kwae Estate. This is stipulated in the contractual terms under which GOPDC provides seedlings and other agricultural inputs to these outgrowers. The other independent oil palm smallholders can decide whether to sell their harvest to the GOPDC mill, other licensed oil palm buyers (e.g. Oboama Company) or small independent processors depending on favourable price signals. GOPDC currently employs over 4500 staff member, of which over 70% is occupied in plantation-related activities such as harvesting, fertilizer application, and weeding. GOPDC plantation workers are paid salaries on a monthly basis, with the salary level being calculated through daily attendance records. Poverty rates are fairly low compared to other sites (and the rest of the country), as the district registers a poverty incidence rate (16.6%) [40].

### Data collection and handling

In order to estimate the human wellbeing outcomes of involvement in industrial crop production we estimate various objective and subjective wellbeing measures (see “Data Analysis”). Primary data was collected through surveys with households having different types of involvement in industrial crop value chains. This included plantation workers and industrial crop producers (involved groups), as well as community members not involved in industrial crop production (control groups). Control groups consisted of local community members involved in the main livelihood options in each study site, predominately food crop farming mostly performed under subsistence conditions.

The household survey mostly consisted of close-ended questions that aimed to elicit measures of objective and subjective wellbeing (see “Data Analysis”). Some open-ended questions elicited justifications for some specific close-ended questions. The development of the household survey was informed from preliminary site visits in August 2015, when we undertook informal interviews with local communities to understand the history of crop production and main livelihood options in each area.

Data was collected through extensive fieldwork in each site: Dabala (February-March 2016), Kwae (December 2016—January 2017), Yeji (August-September 2017) (See S1 File for the questionnaire used). In the first two fieldwork rounds, we employed a hardcopy version of the questionnaire, while for the third round we employed an electronic tablet version that was exactly the same. Due to the different configurations of the study projects and the characteristics of the host communities, we used different sampling strategies in order to randomize respondent selection (Table 2). Our sampling approach followed closely the protocol proposed for the elicitation of household-level impacts in industrial crop settings of southern Africa [41]. In a nutshell we purposefully selected intervention and control groups depending on the different characteristics of each study site, including different types of industrial crop smallholders (in Dabala, Kwae) and plantation workers (in Yehi, Kwae) (Table 2). Based on the available information in each site we randomized the selection of respondents for each group following different techniques, including random selection from smallholder/worker lists, snowballing and transect walks [41] (Table 2). In this sense, study group selection was purposive but respondent selection within each group was randomized to the extent possible [41].

**Table 2:** Study groups and sample sizes.

Study site	Group	Code	Communities	Sample per community	Sample per group	Sampling strategy
Dabala (Sugarcane)	Sugarcane smallholders **(Group 1)**	DB_S	Dabala	100	100	We divided each community into 4 zones, using the main roads. The first sugarcane-growing household in each zone was identified randomly through a transect walk starting at the edge of the community. Subsequently we selected 25–27 sugarcane smallholder households in each zone through a transect walk towards the centre of the community. To allow for randomization we selected respondents every 3–4 households following this transect. A similar approach was used to identify control group respondents.
	Food crop farmers within sugarcane area (control group) **(Group 2)**	DB_NS	Dabala	100	100	
Yeji (Jatropha)	Permanent plantation workers in Smart Oil plantation **(Group 3)**	YJ_JPW	Kadue	25	100	Permanent and seasonal workers from the identified communities were randomly selected at the company warehouse and main office as they came to record their daily attendance. We excluded workers from Yeji main city to avoid a sample that would include a combination of respondents from urban and rural areas.
			Kobre	25		
			Agentriwa	25		
			Kojo Boffour	25		
	Seasonal plantation workers in Smart Oil plantation **(Group 4)**	YJ_JSW	Kadue	14	50	
			Kobre	12		
			Agentriwa	12		
			Kojo Boffour	12		
	Food crop farmers within jatropha area (control group) **(Group 5)**	YJ_JC	Kadue	20	100	The first non-worker was randomly selected through a transect walk starting at the edge of the community. We then asked each subsequent respondent for the nearest non-worker neighbor (i.e. snowball sampling).
			Kobre	30		
			Agentriwa	20		
			Kojo Boffour	30		
Kwae (Oil Palm)	Permanent plantation workers for GOPDC plantation **(Group 6)**	KW_GW	Kwae	25	100	Workers congregate at assembly points from each community in work groups (e.g. mill workers, harvesters, security, nursery workers, plantation workers). For each worker group, we randomly selected five respondents from each community as they disembarked from buses.
			Anwean	25		
			Asuom	25		
			Otumi	25		
	Oil palm outgrowers for GOPDC **(Group 7)**	KW_OG	Kwae	25	100	We divided each community into 4 zones, using the main roads. The first out-grower was identified randomly through a transect walk starting from the edge of the community towards the center. Subsequent outgowers were identified every 3–4 houses, selected 5–6 respondents in each zone. A similar approach was used to identify independent smallholders and control groups.
			Anwean	25		
			Asuom	25		
			Otumi	25		
	Oil palm independent smallholders **(Group 8)**	KW_ID	Kwae	25	100	
			Anwean	25		
			Asuom	25		
			Otumi	25		
	Food crop farmers within oil palm area (control) **(Group 9)**	KW_C	Kwae	25	100	
			Anwean	25		
			Asuom	25		
			Otumi	25		
**Total**				**850**	**850**	

In Dabala (sugarcane) we divided the community into four zones based on the major roads traversing it. In each of the four zones we selected 25–27 sugarcane growing and control households through transect walks (see Table 2).

In Kwae we followed a different sampling approach for each study group to ensure the effective randomization of respondents (Table 2). GOPDC plantation workers were randomly sampled from assembly points either after finishing or before starting their shifts (Table 2). There were unique assembly points for workers coming from each of the four local communities around the plantation (i.e. Kwae, Anwean, Asuom, Otumi). In total we distributed 105 questionnaires to plantation workers, targeting between 25–27 workers from each of the four major communities. Subsequently, we selected an approximately equal number of outgrowers, independent smallholders and control groups in these communities through snowball sampling (Table 2). Mapping of the sampled households shows the spatial intersection (and not segregation) of plantation, workers, outgrowers, independent smallholders and control households in each community, with a relatively equal distribution within the respective communities.

In Yeji we followed a more targeted sampling approach. As Yeji is a major city, plantation workers come both from urban and rural areas, so they tend to have different livelihood sources. We avoided sampling respondents from both urban and rural areas as this could hinder proper comparisons with the other sites that are predominately rural. For this reason, we surveyed only workers and control groups from the rural communities of Kadue, Kobre, Agentriwa and Kojo Boffour. In particular, we selected randomly 25 workers from each rural community. Workers were identified at the warehouse and the company office, as they came randomly to record their names after the end of their daily shifts. We then selected control groups in each community through snowball sampling (Table 2).

To avoid sampling households with dual involvement in industrial crop production (e.g. households that grow industrial crops but at least one of their members is employed at a plantation) [41], we asked direct questions at the beginning of the interview. After ensuring that there is no dual involvement we progressed with the main questionnaire. If dual involvement was found, then the enumerators were trained to skip this household/respondent and move to the next one. In all study sites, questionnaires with serious data omissions were eliminated. Household surveys were analysed using SPSS version 25 following the procedures outlined in the following section.

We supplemented the household survey with focus group discussion (FGDs) with 7–10 respondents that elicited further key issues related to industrial crop production in each site. In the GOPDC (oil palm) site, four FGDs were conducted; two with oil palm growers and two with the control groups. In order to capture gender-differentiated perspectives for each group we conducted an FGD only with females and one only with males. In Yeji, we conducted two FGDs with plantation workers (one only with males, and one only with females) and two FGDs with the control groups (one with only males, and one only with females). In Dabala (sugarcane) site, we conducted two FGDs with sugarcane growers (one only with males, and one only with females) and two FGDs with the control groups (one with only makes, and one only with females).

### Data analysis

#### Income and expenditure analysis

Many scholars have reported the difficulty in capturing income accurately in agrarian settings of SSA [42,43]. Recent studies have indicated the tendency to under-report rural incomes [44,45], partly due to the fact that households in agrarian context of SSA tend to have multiple small income streams that cannot be captured fully or accurately.

For this study, we derive total household income by adding all the different distinct income streams including income from the sales of food crops, sales of industrial crops (in Kwae and Dabala), salaried work in industrial crop plantations (in Yeji and Kwae), sales of natural products (e.g. fuelwood, medicinal plants), sales of livestock/poultry, own-business (i.e. petty trading), pension and remittances. For comparative purposes we use the poverty lines provided by the Ghana Statistical Service [40] rather than an international cut-off measure, e.g. the World Bank’s USD 1.90 per day [46]. Based on their aggregate income we then divide households into four quartiles using the SPSS descriptive function (i.e. 1^st^ quartile “very low-income group”, 2^nd^ quartile “low income group”, 3^rd^ quartile “medium income group”, 4^th^ quartile “high income group). Subsequently we use the independent two samples *t*-test to identify whether the differences in mean income are statistically significant between groups.

Household expenditures were captured through the aggregation of expenses for farming, food purchases, education, health, housing, clothing, energy (e.g. biomass, electricity), supporting relatives, ceremonies, and communication. We compute household expenditures on the basis of adult consumption equivalent [47–49]. In Ghana, households with an annual adult consumption equivalent of less than GHC 792 and between GHC 792–1,314 are considered to be “extremely poor” and “poor” respectively [40]. We test the statistical difference between the mean adult consumption equivalents using independent two samples *t*-test.

Although t-tests can give some indication of differences between treatment and control groups, it is not enough to provide robust statistical evidence of the livelihood impacts of involvement in industrial crop production. This is because issues related to endogeneity, self-selection or systematic bias when selecting respondents [50]. To correct such biases, we employ the propensity score matching (PSM) technique, which has been used in several recent studies to assess the impact of agricultural interventions in SSA [51–54].

In this study we assess how involvement in industrial crop production can have an impact on household total income, income per capita, total consumption and consumption per capita. Household decisions to cultivate an industrial crop fits within the theory of farm households, and can thus be modeled using the random utility framework [55]. In this case, households are not only regarded as producers, but also as consumers that are expected to make production decisions that maximize their utility [56]. As rational decision-makers, households will engage in industrial crop cultivation only if it maximizes their utility. The decision to engage in industrial crop production (or not), is a binary variable, and is affected by other variables such as farm size and the age, gender, and education of the primary decision-maker in the household. Such outcome variables can be expressed as a function of the binary decision-making variable along with other explanatory variables and can be expressed as;

$$Y_{h}=\gamma X_{h}+\delta V_{h}+\mu_{h}$$ (1)

where *Y*_*h*_ is the outcome variable, *V*_*h*_ is the binary decision variable for industrial crop cultivation, *X*_*h*_ is a set of matching variables (confounders) and *μ*_*h*_ is represents random noise. In such estimations, it is highly possible to observe an endogeneity problem due to self-selection. The *μ*_*h*_ may correlate with *V*_*h*_ and/or *Y*_*h*_ which may lead to biases in the estimations [57,58]. The propensity score estimation is thus used to overcome this problem.

Following Hirano and Imbens [59], in estimating intervention impacts using propensity score matching, the Average Treatment Effect (ATE) is expressed as;

$$ATE=E\left[Y_{i}\left(1\right)-Y_{i}\left(0\right)\right]$$ (2)

where *Y*_*i*_(1) is the outcome for ith individual/household that is involved in industrial crop production and *Y*_*i*_(0) for ith individual/household that is not involved in industrial crop production.

However, it is difficult to estimate ATE because both involvement and non-involvement cannot be observed at the same time hence the counterfactuals are used. Thus in our study we estimate the Average Treatment effect on the treated (ATT) to evaluate the effect of the treatment on the population that is involved in industrial crop production. It is expressed as follows;

$$ATT=E\left[Y_{i}\left(1\right)-Y_{i}\left(0\right)|T_{i}=1\right]$$ (3)

$$=E\left[E\{Y_{i}\left(1\right)-Y_{i}\left(0\right)|T_{i}=1,p\left(X\right)\}\right]$$ (4)

$$=E\left[E\{Y_{i}\left(1\right)|T_{i}=1,p\left(X\right)\}-E\left\{Y_{i}\left(0\right)|T_{i}=0,p\left(X\right)\right\}|K=1\right]$$ (5)

ATT is estimated based on two underlying assumptions. The first is the assumption of conditional independence, which states that given a set of confounders, the outcomes being investigated should be independent of the household decision on the production of industrial crops. Secondly, the common support assumption should be satisfied. This assumption states that the compared respondents have to have similar characteristics in order to compare similar or the same propensity scores [60]. In this study we use the minima and maxima method to check the overlap and common support assumption suggested [61]. The confounders used include age of household head (years), education (measured as a dummy), size of cultivated land (in hectares), fraction of off-farm income in total income (in percent), household size (persons in adult equivalent) (Table A in S2 File). Using probit estimation in the propensity score analysis, we examined the statistical significance of the beta term (Tables B-F in S2 File). We conducted balancing tests with various matching algorithms to identify which one produces lowest mean bias (Tables G-K, Figures A-E in S2 File).

Since standard errors from analytical estimates may be biased, we employ the bootstrapping approach to achieve more consistent standard errors [62]. In this case we use the Rosenbaum bounds method to analyse the sensitivity of the results (Tables H-P in S2 File), as there may still be unobservable variables that might affect the assignment into the treated group and the outcome variable simultaneously [63]. In this case, a hidden bias may be imposed on the results obtained.

#### Multidimensional poverty analysis

There is an increasing proliferation of academic literature on the limitation of poverty measures based on monetary indicators/metrics (e.g. USD/day) [22,64,65]. The non-monetary understanding of poverty, such as the concept of multidimensional poverty, has gained momentum in the past decade.

An increasingly popular metric of multidimensional poverty is the Adjusted Headcount Ratio (Mo), also called Multidimensional Poverty Index (MPI), which is based on the methodology pioneered by Alkire and Foster [64]. This approach essentially counts the number of people within a group that suffer from different dimensions of deprivation, as well as the number of dimensions in which they fall below a certain threshold [66,67].

In this study we used a modification of the MPI aggregating 10 indicators across three different dimensions of deprivation (Table 3) [35,68]. The only change from the conventional MPI is that we used the food consumption score (FCS) as a proxy for nutrition rather than the Body Mass Index of adults [22]. We used a poverty cut-off of 33.33%, implying that a household is poor if it scores above this threshold [69]. Below we briefly outline the main equations used in this study and see S3 File for data.

**Table 3:** Multidimensional poverty dimensions, indicators, weights and deprivation cut-offs.

Dimension	Indicator	Deprivation cut-offs	Weight
Education	Years of schooling	If no household members who have completed 5 years of schooling	1/6
	Child school attendance	If any school-age child is not attending school in years 1–8	1/6
Health	Nutrition	Below the acceptable Food Consumption Score (FCS) i.e. an FCS of 35 or below	1/6
	Child mortality	A child has died in the family in the last 5 years	1/6
Living Standards	Improved drinking water	The household does not have access to improved drinking water (according to MDGs guidelines), or safe drinking water is more than a 30 min walk from home (round trip)	1/18
	Improved sanitation	The household's sanitation facility is not improved (according to MDG guidelines), or is improved but shared with other households	1/18
	Clean cooking fuel	The household cooks with dung, charcoal or wood	1/18
	Electricity	The household has no electricity.	1/18
	Flooring material	The flooring material is made of dirt, sand or dung	1/18
	Asset ownership	The household does not own more than one radio, TV, telephone, bike, motorbike or refrigerator, and does not own a car, truck or tractor	1/18

The Adjusted Headcount Ratio (Mo), is determined based on Eq (6),

Mo=H×A (6)

where “H” denotes the incidence of poverty representing the percentage of the population that is poor in a sample (see Eq 7). and “A” denotes the intensity of deprivation across the poor (see Eq 8).

H=q/n (7)

where *q* denotes the number of people identified as poor and *n* the total number of people in the sample

$$A=\sum_{i=1}^{n}\frac{C_{i}\left(k\right)}{q}$$ (8)

where *C_i_*(*k*) denotes the censored deprivation score which (*k*) indicates the share of possible deprivation experienced by the poor person *i*.

Considering the Eqs (6)–(8), the Adjusted Headcount Ratio (Mo) is expressed as:

$$M_{0}=\sum_{i=1}^{n}\frac{C_{i}\left(k\right)}{q}=HxA=\frac{q}{n}x\frac{1}{q}\sum_{i=1}^{q}C_{i}\left(k\right)=\frac{1}{n}\sum_{i=1}^{n}C_{i}\left(k\right)$$ (9)

Several studies have already reported the detailed steps for using the MPI in terms of whether the household is deprived in a specific indicator (deprivation focused), the number of indicator dimensions (poverty-focused) and ignoring of households not meeting the dual cut-offs in MPI calculations. The reader interested in the methodology of the MPI is referred to [35,64,68,69].

To explore whether groups involved in industrial crop production were significantly better off than the control groups, we used the bootstrap resampling approach for statistical inference of multi-dimensional poverty between groups [70]. We also perform a robustness test to check how robust are the MPI results in respect to changes in the weights of the indicators [48,68,71]. We perform a restricted dominance analysis by varying the weights of the indicators (Table 4) [22]. The new MPI rankings are compared with the original MPI by completing a Spearman rank correlation coefficient [22,71].

**Table 4:** Weighting variation for restricted dominance analysis.

Weighting Type	Dimension		
	Education	Health	Living Standards
Original weighting	33.33%	33.33%	33.33%
Alternative weighting 1	50%	25%	25%
Alternative weighting 2	25%	50%	25%
Alternative weighting 3	25%	25%	50%

As outlined above, the MPI calculation procedure aggregates the deprivation scores of poor households into a single index, which is obtained by multiplying the multidimensional poverty headcount ratio and the intensity of poverty. This makes it impossible to obtain individual MPIs for each household, as for other indicators of objective wellbeing (e.g. income, consumption). Therefore it is impossible to undertake PSM analysis using the MPI as an outcome variable, as performed for the other objective wellbeing indicators (see “Income and expenditure”). This means that caution needs to be paid when using the results of this study to understand whether there is a causal relationship between involvement in industrial crop production is an agent of poverty alleviation in terms of MPI.

#### Subjective wellbeing analysis

Several studies have made the point that measures of subjective wellbeing (SWB) need to be used alongside measures of objective wellbeing [23–25]. In our study we assessed subjective wellbeing asking questions about the life satisfaction, happiness, anxiousness and worthwhileness. These dimensions generally reflect the broader theoretical basis for subjective wellbeing measures [49,72,73]. Life satisfaction and happiness are closely related to the self-evaluation approach for measuring SWB. Anxiousness and worthwhileness relate to the experience and eudemonic measures. We, therefore, adopted the four questions recommended by Dolan & Metcalfe [73].

(i) Overall, how satisfied are you with your life nowadays?

(ii) Overall, how happy did you feel yesterday?

(iii) Overall, how anxious did you feel yesterday?

(iv) Overall, how worthwhile are the things that you do in your life?

We used a 4-level Likert scale to assess each indicator (not at all, very little, moderately satisfied and very satisfied). We then used descriptive statistics based on the distribution of the responses for each dimension [74–77]. Subsequently, we used the Mann-Whitney *U* test to assess whether the self-reported levels for each indicator were statistically significant between groups.

We do not undertake the PSM analysis for the different indicators of subjective wellbeing. First of all, subjective wellbeing can be affected by many factors that go beyond household livelihoods (i.e. adoption of industrial crops) such various cultural, socioeconomic, environmental and personal factors [78]. Furthermore, it is very difficult to link changes in subjective wellbeing with specific interventions [79]. This makes it very complicated to decide, which variables would be appropriate to perform the matching. Considering the above we use the subjective wellbeing indicators as an added layer of analysis to complement the objective wellbeing indicators, identifying interesting patterns between them for the different study groups. Similar to poverty (see “Multi-dimensional poverty analysis”), caution should be exercised when interpreting the subjective wellbeing indicator patterns.

### Ethical considerations and permissions

The development of the research protocol was informed by a similar protocol that elicited the impact of industrial crops in southern Africa [41], and adopted the good practice recommendations of the UK Economic and Social Science Research Council (ESRC). The research protocol was reviewed and approved by the Ethics Review Committee of the Graduate School of Frontier Sciences, University of Tokyo (identification number 15–186). This included approval of obtaining verbal consent from survey participants. The protocol was granted approval in December 2015, before the full surveys commenced. Prior to the commencement of the study we checked the necessary regulations for this type of research in Ghana. Considering that our study was not medically-oriented (i.e. we conducted household surveys that collected socioeconomic variables), our understanding after consulting the appropriate authorities in Ghana was that the ethical clearance received through our institution in Japan sufficed.

To ensure the smooth and proper community entry, prior to contacting respondents we undertook meetings with traditional authorities and representatives of the private companies and farmers’ associations operating in each study site. This was necessary as local chiefs are influential members of the local communities in Ghana. Furthermore, we sought the permission of the two industrial crop companies in Kwae (GOPDC) and Yeji (Smart Oil) to avoid causing problems to the interviewed workers. Eventually we were allowed to interview plantation workers in company estates (Table 2), which was possible only after receiving their permission.

We sought oral informed consent from all respondents before each interview, explaining the purpose and procedures of the research. Oral consent was considered as the most appropriate approach considering the high illiteracy rates in the local communities. Through this process seeking oral informed consent we ensured that all respondents were fully aware what their participation in this study entailed, and explained any concerns related to anonymity and disclosure of personal information. We did not interview any minors. The age ranges of the respondents in each site are: Dabala: 24–73 years; Yeji: 21–90 years; Kwae: 20–75 years.

Participation in the household survey was voluntary. Respondents reserved the right to decline being interviewed, and were informed that possible refusal would not lead to any adverse consequences. No payments were made to participants and all surveys were anonymised to avoid the identification of respondents.

## Results

### Basic household characteristics

Tables 5 and 6 include the basic household characteristics for the different study groups and the statistical differences of their means. In terms of the age and education of the household head there are significant differences only between groups in the oil palm area (Table 6). The heads of GOPDC worker households (KW_GW) tend to be significantly more educated, with over 87% (*n* = 100) having at least some level of formal education (i.e. at least basic education).

**Table 5:** Basic household characteristics for each study group.

Case Study	Group	Household Composition			Land (ha)		
		Total size	Adults	Children	Total	Cropland	Unused
**Dabala (Sugarcane)**	DB_S	5.1±1.5	3.2±1.1	1.9±.1.4	3.2±2.6	2.0±1.7	1.2±0.7
	DB_NS	5.0±1.9	3.3±1.2	1.7±1.2	2.9±2.4	2.2±1.6	0.7±1.3
**Yeji (Jatropha)**	YJ_JPW	5.7±3.0	2.5±1.2	3.2±2.2	3.6±1.6	1.4±0.9	2.2±1.3
	YJ_JSW	5.8±2.9	2.3±1.2	3.5±2.2	2.5±1.7	2.0±1.4	0.5±0.5
	YJ_JC	6.3±2.8	2.4±1.0	3.9±2.3	3.5±3.1	3.1±3.1	0.4±0.4
**Kwae (Oil Palm)**	KW_GW	2.5±1.0	2.1±0.5	0.4±0.7	0.6±1.3	0.5±1	0.1±0.5
	KW_OG	4.4±1.8	3.1±.14	1.3±1.2	7.2±3.9	4.1±2.4	3.1±2.3
	KW_ID	4.4±1.8	2.9±1.2	1.5±1.2	7.1±3.7	3.5±2.7	3.6±1.8
	KW_C	4.2±2.0	2.8±.14	1.4±1.4	4.9±4.5	1.9±0.9	3.0±2.5

**Table 6:** Statistical difference of means for basic household characteristics.

Case Study	Groups	Household Head		Household Members			Household Land (ha)		
		Age	Education	Total	Adult	Children	Total	Cropland	Unused
Dabala (Sugarcane)	DB_S vs DB_NS	0.228	0.256	0.551	0.794	0.791	0.118	0.274	0.028**
Yeji (Jatropha)	YJ_JPW vs YJ_JSW	0.312	0.170	0.765	0.139	0.370	0.000***	0.001***	0.000***
	YJ_JPW vs YJ_JC	0.535	0. .131	0.068	0.995	0.025**	0.007***	0.000***	0.000***
	YJ_JSW vs YJ_JC	0.014	0.905	0.235	0.150	0.365	0.021**	0.008*	0.347
Kwae (Oil Palm)	KW_GW Vs KW_OG	0.864	0.000***	0.000***	0.000***	0.000***	0.000***	0.000***	0.000***
	KW_GW vs KW_ID	0.001***	0.000***	0.000***	0.000***	0.000***	0.000***	0.000***	0.000***
	KW_GW vs KW_C	0.337	0.000***	0.000***	0.000***	0.000***	0.000***	0.000***	0.016**
	KW_OG vs KW_ID	0.001***	0.481	0.931	0.734	0.205	0.314	0.022**	0.428
	KW_OG vs KW_C	0.412	0.888	0. .218	0.263	0.737	0.000***	0.000***	0.259
	KW_ID vs KW_C	0.013**	0.397	0.150	0.495	0.095	0.000***	0.000***	0.057*

Note: Based on Mann Whitney *U* test for the comparison of mean ranks as data is not normally distributed.

***: p<0.01

**: p<0.05

* p<0.1

Overall the smallest household sizes are reported in Kwae (oil palm), with GOPDC workers having the smallest households, largely due to the fact that most originate from other parts of Ghana. Worker households usually consist of adult members with few children. With the exception on workers, there are no other significant differences in terms of household sizes and composition between the other study groups (i.e. KW_C, KW_OG, KW_ID). Similarly, there is no significant difference in total household size and composition between sugarcane smallholders and the control group in Dabala. On the contrary there are significant differences in household size and composition between permanent jatroph workers (YJ_JPW) and the control group in Yeji. Overall, the jatropha site exhibits the largest mean household sizes compared to other sites, at 5.7, 5.8 and 6.3 persons per household for permanent workers, seasonal workers and the control group respectively. At the same time the study groups in Yeji have the highest fraction of children to adult members among all sites. These patterns reflect well the context of Ghana, where households in the savanna ecological zone (where Yeji is located) have larger household sizes and higher fertility rates compared to other parts of the country [39,80].

When it comes to land ownership there is no significant difference in total land size and cropland between sugarcane smallholders and control groups in Dabala (Tables 5 and 6). In Yeji (jatropha site), there is a significant difference in land ownership, in terms of total land size, cropland and unused land (Table 6). Permanent plantation workers have, on average, more unused land (2.2 ha) and less cropland (1.4 ha) compared to the other groups. This is partly due to the fact that their plantation employment does not allow them enough time for food crop farming as indicated in open-ended questions and the FGDs. In Kwae (oil palm site), GOPDC workers have significantly less land (0.6 ha) than the other study groups. This is largely due to the fact that 60% of GOPDC worker respondents originate from other parts of Ghana, migrating in Kwae for work or marriage. The control group in Kwae reported relatively small total land (4.9 ha) and cropland (1.9 ha) sizes, which possibly explains why they have not adopted oil palm cultivation (see next sections for more extensive discussion).

### Income and consumption

#### Mean income and consumption analysis

**Fig 2:**
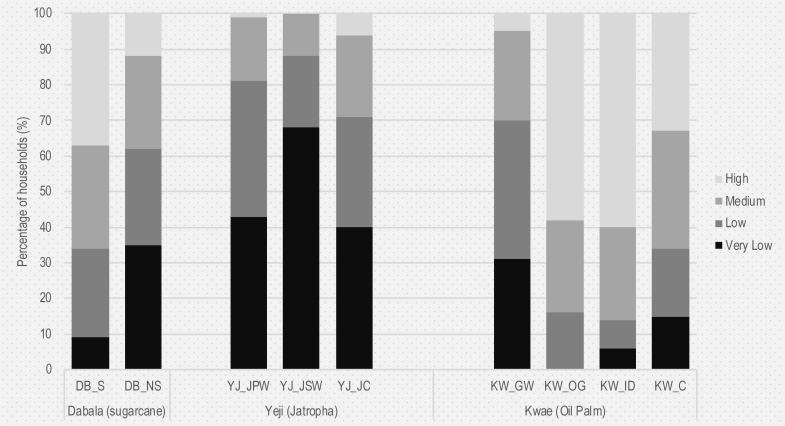
Group distribution across income quartiles.

Table 7 highlights the mean annual income, adult consumption equivalent and poverty rates for the different groups across all sites. Table 8 identifies whether the differences in mean income and consumption levels are statistically significant between the study groups in each site. Fig 2 outlines the distribution of groups across different income quartiles following the procedure outlined in the sub-section “Income and expenditure”.

**Table 7:** Mean annual income, consumption and incidence of poverty for each study group.

Case Study	Group	Mean annual income per household (GHC)	Mean Annual Income per family member (GHC)	Mean Adult Consumption Equivalent (GHC)	Incidence Rate (%)		
					Total	Extremely Poor	Poor
**Dabala (Sugarcane)**	DB_S	10648.1±6488.1	2347.4±1900	1103.9±227.2	87	1	86
	DB_NS	6386.3±3253.9	1415.9±870	988.4±318.6	90	25	65
**Yeji (Jatropha)**	YJ_JPW	5086.8±1759.9	1142.0±930.7	827.9±410.6	92	54	38
	YJ_JSW	4275.4±1790.2	1043.8±979.5	819.4±534.0	90	76	14
	YJ_JC	5907.3±3018.9	1254.9±1357.0	1080.8±769.0	81	37	44
**Kwae (Oil Palm)**	KW_GW	5834.5±2501.1	2585.1±1263.8	1663.8±869.9	31	7	24
	KW_OG	12915.2±6051.0	3331.6±1982.8	2275.6±984.1	1	0	1
	KW_ID	13429.9±7071.1	3474.6±2151.8	1979.3±479.6	3	0	3
	KW_C	9092.5±4424.2	2714.5±2056.4	1682.0±462.9	21	1	20

**Table 8:** Statistical difference between groups for income and consumption.

Case Study	Group Comparison		Mean annual income per household (P value)	Mean Annual Income per family member (P value)	Mean Adult Consumption Equivalent (P value)
**Dabala (Sugarcane)**	**1 vs 2**	DB_S	0.000***	0.000***	0.000***
		DB_NS			
**Yeji (Jatropha)**	**3 vs 4**	YJ_JPW	0.000***	0.573	0.163
		YJ_JSW			
	**3 vs 5**	YJ_JPW	0.283	0.494	0.012**
		YJ_JC			
	**4 vs 5**	YJ_JSW	0.000***	0.346	0.001***
		YJ_JC			
**Kwae (Oil Palm)**	**6 vs 7**	KW_GW	0.000***	0.002***	0.000***
		KW_OG			
	**6 vs 8**	KW_GW	0.000***	0.000***	0.000***
		KW_ID			
	**6 vs 9**	KW_GW	0.000***	0.593	0.074*
		KW_C			
	**7 vs 8**	KW_OG	0.759	0.626	0.076*
		KW_ID			
	**8 vs 9**	KW_OG	0.000***	0.032**	0.000***
		KW_C			
	**7 vs 9**	KW_ID	0.000***	0.011**	0.000***
		KW_C			

Note: Based on Mann Whitney *U* test for the comparison of mean ranks as data is not normally distributed.

***: p<0.01

**: p<0.05

* p<0.1

In the Dabala site (sugarcane), sugarcane growers report significantly higher mean income levels compared to the control group (Table 8). Furthermore, income distribution shows very different patterns between groups, with a substantially higher proportion of control group respondents falling within the “very low” and “low” income brackets, when compared to sugarcane producers. For sugarcane growers, the actual income received from sugarcane sales amounts, on average, to 59% of their total income, with only 14% coming from food crop sales. On the other hand, income from food crop sales constitutes approximately 51% of the total mean income of the control, with other significant income sources being livestock sales (16%) and remittances (15%). Actually the mean income received from sugarcane amounts to GHC 6237 per year, which is 40% higher than the combined incomes that the control group respondents receive on average from food crop and livestock sales.

In Yeji (jatropha), control group respondents report substantially higher mean income compared to permanent and seasonal workers (Table 7). Actually most of the workers fall in the “very low income” and “low-income” categories. For permanent and seasonal workers, annual salaries constitute respectively 59% (mean salary = GHC 2878) and 46% (mean salary = GHC 1783) of total household income. Workers report lower income generated from food crop and livestock sales compared to the control group. Food crop and livestock sales constitute on average 67% and 29% respectively of the total annual mean income of control group respondents. It is worth noting that the mean income of seasonal workers is substantially lower to that of permanent workers. This, combined with the fact that seasonal workers have on average the smallest landholdings in the area, suggests that it is the relatively less endowed households that are willing to engage in seasonal plantation work.

In Kwae (oil palm), mean income levels are very significantly different between all groups, with the exception of outgrowers (KW_OG) and independent smallholders (KW_ID) (Table 8). Oil palm outgrowers and independent smallholders have both the highest mean income and proportion of households in the “high-income” quartile in Kwae. At the same time their income sources are slightly more diversified, as income from involvement in oil palm activities constitutes on average 75% of the overall household income, compared to 81% for GOPDC workers. It is worth noting that even though the control group reports a higher mean total annual income compared to workers (Table 7), there is no statistical difference in annual income per family member due to the relative low household size of oil palm workers (Table 8).

Table 7 reports the mean adult consumption equivalent with poverty lines adopted from the Ghana Statistical Service. In Dabala (sugarcane), the proportion of households that are below the poverty line is high for both sugarcane smallholders (87%) and the control group (90%). However, those involved in sugarcane production have a significantly higher mean adult consumption equivalent (mean of 1103.9 vs. 988.4 GHC/year). In Yeji (jatropha), the poverty incidence rates are over 90% for households engaged in jatropha employment and over 80% for the control group. At the same time the control group has a significantly higher mean adult consumption equivalent than permanent and seasonal workers (Table 7). In Kwae, oil palm outgrowers and independent smallholders have significantly lower incidence of consumption poverty compared to GOPDC workers and the control group (Table 7). Actually, only 1% of outgrowers and 3% of independent smallholders could be characterized as poor, compared to 21% and 31% for the control group and GOPDC workers respectively (Table 7).

#### Propensity score matching analysis

We use the PSM technique to assess whether there is a causal relationship between involvement in industrial crop production and improvement in the four objective wellbeing indicators. The PSM analysis for sugarcane (DB_S) and non-sugarcane (DB_NS) producers uses the radius caliper (0.1) matching algorithm and suggests that engagement in sugarcane production leads to both significantly higher total household income (by GHC 3512.62, p<0.01) and per capita household income (by GHC 731.77, p<0.01) (Table 9). Similarly total household consumption and per capita household consumption increases by GHC 294.16 (p<0.1) and GHC 81.51 (p<0.1) respectively (Table 9). These results imply that involvement in smallholder-based sugarcane production increases significantly objective wellbeing.

**Table 9:** Propensity score matching (PSM) analysis of the impact of involvement in industrial crops on household income and consumption.

Variable	Groups (observations after common support)	Treatment effect	Balancing Test				Rosenbaum bounds (Gamma)
		Difference (GHC)	Pseudo R^2^	p-Value LR*	Mean Bias	Comment	
Total Household Income	YJ_JPW (94) & YJ_JC (79)	500.78 (313.31)	0.003	0.976	4.7	Good Matching	1.20
	DB_S (88) & DB_NS (98)	3512.62*** (519.85)	0.002	0.992	2.6	Good Matching	3.10
	KW_W (29) & KW_C (99)	-970.77 (1042.41)	0.028	0.815	15.0	Weak matching	1.30
	KW_OG (19) & KW_C (67)	-1436.35** (693.58)	0.037	0.858	13.6	Weak matching	1.60
	KW_ID (27) & KW_C (84)	-39.73 (825.79)	0.007	0.992	6.8	Weak matching	-
Per Capita Income	YJ_JPW (94) & YJ_JC (79)	146.95 (144.40)	0.003	0.976	4.7	Good Matching	-
	DB_S (88) & DB_NS (98)	731.77*** (129.48)	0.002	0.992	2.6	Good Matching	2.30
	KW_W (29) & KW_C (99)	-483.481 (493.15)	0.028	0.815	15.0	Weak matching	1.50
	KW_OG (19) & KW_C (67)	-833.14* (454.32)	0.037	0.858	13.6	Weak matching	2.00
	KW_ID (27) & KW_C (84)	-54.79 (397.36)	0.007	0.992	6.8	Weak matching	-
Household consumption	JPW (94) & KW_JC (79)	-578.09** (227.58)	0.003	0.976	4.7	Good Matching	2.10
	DB_S (88) & DB_NS (98)	294.16* (162.30)	0.002	0.992	2.6	Good Matching	1.40
	KW_W (29) & KW_C (99)	-93.09 (371.22)	0.028	0.815	15.0	Weak matching	-
	KW_OG (19) & KW_C (67)	29.97 (446.39)	0.037	0.858	13.6	Weak matching	-
	KW_ID (27) & KW_C (84)	-551.64 (365.66)	0.007	0.992	6.8	Weak matching	1.20
Per capita consumption	JPW (94) & KW_JC (79)	-166.67 (101.97)	0.003	0.976	4.7	Good Matching	2.00
	DB_S (88) & KW_NS (98)	81.51*(41.68)	0.002	0.992	2.6	Good Matching	1.50
	KW_W (29) & KW_C (99)	-26.49 (151.64)	0.028	0.815	15.0	Weak matching	-
	KW_OG (19) & KW_C (67)	-0.31 (152.18)	0.037	0.858	13.6	Weak matching	-
	KW_ID (27) & KW_C (84)	-183.31 (148.41)	0.007	0.992	6.8	Weak matching	1.20

Note:

***: p<0.01

**: p<0.05

* p<0.1

The PSM estimation for permanent jatropha workers (YJ_JPW) and the control group (YJ_JC) suggests that engagement in permanent jatropha employment leads to higher total income (by GHC 500.78) and per capita income (by GHC146.95), but this is not statistically significant (Table 9). To achieve the common support assumption we conducted the analysis within the range of 0.0352 and 0.9006. The results suggest that the radius caliper estimation technique provides a better matching with reduced bias, compared to the kernel and nearness neighbour algorithm. However, engagement in permanent jatropha employment decreases both total household consumption (lower by GHC578.09, p<0.05) and per capita consumption (by GHC 166.67), with both results not being significant (Table 9). This can be possibly explained by the fact that jatropha workers have, on average, much smaller cultivated cropland and more unused land (Table 5), hence investing and spending much less in their farms.

The PSM analysis in Kwae suggests that involvement in oil palm production as workers (KW_W), outgrowers (KW_OG) and independent smallholders (KW_ID) leads to declining levels of most objective wellbeing indicators (Table 9). The PSM analysis of oil palm workers (KW_GW) and the control group (KW_C) was done within 0.0021 and 0.8289 range. For oil palm outgrowers (KW_OG) and the control group (KW_C) the analysis was done within the 0.0093 and 0.75515 range. For oil palm independent smallholders (KW_IG) and the control group (KW_C) the analysis was done in the 0.0112 and 0.7618 range. However, with few exceptions most of these results are not statistically significant (Table 9). These patterns are somewhat expected for plantation workers, as paid employment for GOPDC is usually the last resort for migrants or less endowed local households. However, it is somewhat surprising for oil palm outgrowers and independent smallholders. A possible explanation could be the land requirement of oil palm and declining oil palm prices (see sub-sections “Patterns between groups in each site” and “Patterns between sites”). We should note, however, that the PSM analysis in Kwae did not produce as good matches as in the other two sites. The common support assumption achieved with the minima and maxima approach [51,69] resulted in the loss of most treated observations (more than 60%) (Table 9).

### Multidimensional poverty

**Fig 3:**
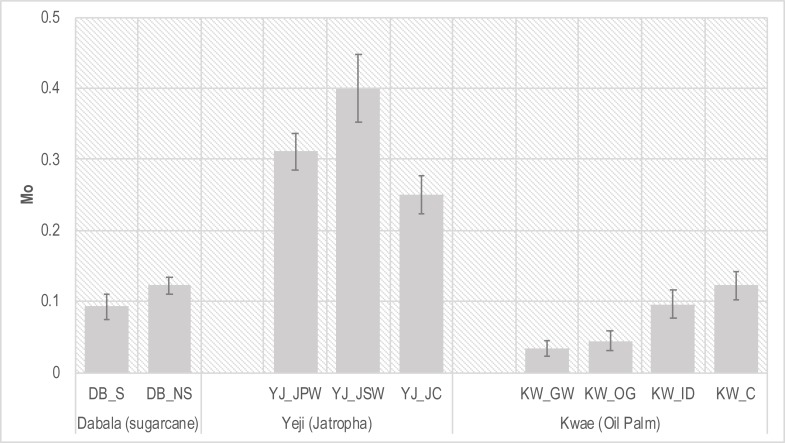
Multi-dimensional poverty index for each study group.

Table 10 and Fig 3 contain the aggregated MPI at the 99% confidence interval. Sugarcane smallholders in Dabala exhibit lower levels of multi-dimensional poverty (0.093) compared to the control group (0.123). In Yeji, the control group has a lower MPI (0.250) compared to both permanent (YJ_JPW) (0.311) and seasonal workers (YJ_JSW) (0.400). However, the permanent workers (YJ_JPW) tend to be less multi-dimensionally poor (0.311) than the seasonal workers (YJ_JSW) (0.400). In Kwae (oil palm), all groups involved in oil palm production, whether as outgrowers (KW_OG, 0.045), independent smallholders (KW_ID, 0.096), or workers for GOPDC (KW_GW, 0.034) exhibit lower MPI than the control group (KW_C, 0.123), with GOPDC workers reporting the lowest levels among all groups.

**Table 10:** Multi-dimensional poverty index and confidence intervals for each study groups.

Case Study	Group	Mo	Standard error	99% Confidence Interval	
				Lower bound	Upper bound
**Dabala (Sugarcane)**	DB_S	0.093	0.0173	0.0545	0.1417
	DB_NS	0.123	0.0116	0.0083	0.0656
**Yeji (Jatropha)**	YJ_JPW	0.311	0.0266	0.2429	0.3785
	YJ_JSW	0.400	0.0470	0.2735	0.5202
	YJ_JC	0.250	0.0271	0.1845	0.3196
**Kwae (Oil Palm)**	KW_GW	0.034	0.0116	0.0083	0.0656
	KW_OG	0.045	0.0136	0.0139	0.0839
	KW_ID	0.096	0.0200	0.0472	0.1489
	KW_C	0.123	0.0197	0.0761	0.1834

In summary, all groups engaged in industrial crop production (whether as plantation workers or industrial crop growers) exhibit very different poverty levels compared to their respective control groups. Groups involved in sugarcane and oil palm production are better off than their respective control groups, while the opposite is true for jatropha workers. In fact, among all groups seasonal workers in the jatropha plantation (YJ_JSW) have the highest MPI (0.400). To check the robustness of the MPI results, we change indicator weights (Table 4). Table 11 shows that the MPI results are generally robust with respect to weight changes, as there were only two alternative weight changes in Yeji (Table 11).

**Table 11:** Multi-dimensional poverty index based on indicator weight variation.

Case Study	Group	Original MPI (Mo)	Alternative weight 1	Alternative weight 2	Alternative weight 3
**Dabala (Sugarcane)**	DB_S	0.093	0.103	0.082	0.135
	DB_NS	0.123	0.115	0.135	0.143
**Yeji (Jatropha)**	YJ_JPW	0.311	0.369	0.217	0.413
	YJ_JSW	0.400	0.206	0.362	0.497
	YJ_JC	0.250	0.263	0.202	0.402
**Kwae** **(Oil Palm)**	KW_GW	0.034	0.043	0.051	0.037
	KW_OG	0.045	0.056	0.057	0.044
	KW_ID	0.096	0.092	0.135	0.094
	KW_C	0.123	0.124	0.141	0.119

Table 12 highlights the high levels of deprivation for living standard, particularly for those indicators related to access to improved drinking water, sanitation and clean cooking fuels. The only exception is GOPDC workers (KW_GW), which was found to have lower deprivation in living standards compared to other groups. This is possibly due to their access to the different social amenities developed by the company such as schools, pipe water supply and hospital.

**Table 12:** Deprivation against individual indicators for each study group.

Case Study	Group	Education		Health		Living Standards					
		Years of schooling (%)	Child school attendance (%)	Nutrition (%)	Child mortality (%)	Improved drinking water (%)	Improved sanitation (%)	Clean cooking fuel (%)	Electricity (%)	Flooring materials (%)	Assets ownership (%)
**Dabala (Sugarcane)**	DB_S	11	18	9	14	33	67	97	37	13	1
	DB_NS	11	17	16	21	18	59	95	39	14	1
**Yeji** **(Jatropha)**	YJ_JPW	53	56	5	3	100	97	100	49	13	0
	YJ_JSW	54	54	42	8	100	94	100	86	10	0
	YJ_JC	39	39	2	10	100	95	100	67	18	0
**Kwae** **(Oil Palm)**	KW_GW	8	10	11	5	13	12	31	19	3	15
	KW_OG	9	9	5	14	31	46	97	9	1	2
	KW_ID	8	18	12	25	20	55	91	12	4	3
	KW_C	4	26	33	9	23	52	69	21	13	7

**Note:** Expressed as a fraction (%) of the total population in each group experiencing the specific type of deprivation

In Dabala the patterns of deprivation across the different MPI dimensions is fairly similar for sugarcane smallholders and the control group, with both groups exhibiting high deprivation for access to clean energy and improved sanitation.

In Yeji, the patterns of deprivation are almost identical between groups for the living standards dimension. Overall, while it is not clear-cut, the control group tends to have the lowest deprivation scores for most indicators. It is also worth mentioning that compared to all other sites, groups in Yeji have the highest deprivation for education, largely due to the fact that some of the sampled communities do not have basic school facilities in their vicinity.

Finally, in Kwae (oil palm), deprivation patterns are quite mixed between groups. One interesting finding is that GOPDC workers have low deprivation for access to clean drinking water, sanitation, and clean cooking fuel as the company has different facilities within the estate for social service delivery such as schools, pipe water supply and hospital. For most other indicators oil palm outgrowers (KW_OG) and independent smallholders (KW_ID), have lower deprivation compared to GOPDC workers (KW_GW) and the control group (KW_C).

### Subjective wellbeing

**Fig 4:**
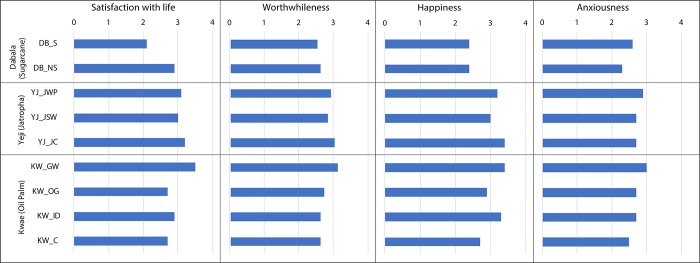
Mean subjective wellbeing indicator scores for each study group.

Fig 4 contains mean scores for each indicator of subjective wellbeing for each group, and Table 13 the score distribution. We observe that most groups mainly report moderate levels of satisfaction with life, with only two groups (i.e. GOPDC workers, control group in Yeji) reporting high satisfaction with life. Similarly, most groups report moderate levels of worthwhileness, happiness and anxiousness (Table 13).

**Table 13:** Distribution of responses for each subjective wellbeing indicator (in %).

Dimension	Scale	Dabala (Sugarcane)		Yeji (Jatropha)			Kwae (Oil Palm)			
		DB_S	DB_NS	YJ_JWP	YJ_JSW	YJ_JC	KW_GW	KW_OG	KW_ID	KW_C
**Satisfaction with life**	Not at all	28	11	0	0	8	1	9	2	7
	Somewhat	41	17	18	16	10	15	33	34	35
	Moderate	24	45	53	64	38	21	33	32	38
	Very	7	27	29	20	44	63	25	32	20
**Worthwhileness**	Not at all	10	13	0	2	7	2	7	3	8
	Somewhat	41	33	26	22	13	26	29	44	35
	Moderate	36	35	57	70	53	35	52	42	48
	Very	13	19	17	6	27	37	12	11	9
**Happiness**	Not at all	14	25	0	2	3	0	9	4	3
	Somewhat	39	26	10	14	7	16	28	19	35
	Moderate	36	36	59	64	40	27	27	25	49
	Very	11	13	31	20	50	57	36	52	13
**Anxiousness**	Not at all	10	15	9	16	24	7	12	14	15
	Somewhat	35	40	13	14	13	25	24	22	26
	Moderate	38	41	56	58	35	26	41	49	51
	Very	17	4	22	12	28	42	23	15	8

However, differences of the mean scores are not always statistically significant between groups (Mann-Whitney *U*, Table 14). In Dabala (sugarcane) we observe significant differences between sugarcane smallholders and the control group only for satisfaction with life and anxiousness. In Yeji (jatropha), the only observed statistically significant difference relates to the levels of happiness between the control group and seasonal workers. In Kwae (oil palm), GOPDC workers exhibit higher levels (and statistically significant difference) for satisfaction with life, worthwhileness and anxiousness. On the other hand, there are only few instances of significant differences among the other groups in Kwae in the mean scores of the different subjective wellbeing indicators.

**Table 14:** Comparison of the levels of subjective wellbeing between study groups.

Case Study	Group Comparison		Dimensions (*p*-value)			
			Satisfaction	Worthwhileness	Happiness	Anxiousness
**Dabala (Sugarcane)**	**1 vs 2**	DB_S	0.000***	0.498	0.664	0.030*
		DB_NS				
**Yeji (Jatropha)**	**3 vs 4**	YJ_JPW	0.505	0.421	0.102	0.099
		YJ_JSW				
	**3 vs 5**	YJ_JPW	0.151	0.151	0.023	0.024
		YJ_JC				
	**4 vs 5**	YJ_JSW	0.062	0.038	0.001***	0.693
		YJ_JC				
**Kwae (Oil Palm)**	**6 vs 7**	KW_GW	0.000***	0.002**	0.000***	0.034
		KW_OG				
	**6 vs 8**	KW_GW	0.000***	0.000***	0.288	0.004**
		KW_ID				
	**6 vs 9**	KW_GW	0.000***	0.000***	0.000***	0.000***
		KW_C				
	**7 vs 8**	KW_OG	0.159	0.281	0.011	0.449
		KW_ID				
	**8 vs 9**	KW_OG	0. .791	0.289	0.099	0.070
		KW_C				
	**7 vs 9**	KW_ID	0.083	0.997	0.000***	0.268
		KW_C				

Note: All *p* values are based on Mann Whitney *U* test because of lack of normally distributed

***: p<0.01

**: p<0.05

* p<0.1

### Correlations between indicators of objective and subjective wellbeing

Tables P-Y in S2 File, report the correlations between the different indicators for each study group. For most groups there is a strong relationship between the indicators of objective wellbeing (i.e. income, consumption). For some groups there are also strong correlations between some of the indicators of subjective wellbeing. However, there are very few instances of strong relationships between indicators of objective and subjective wellbeing. Still, some interesting strong relationships exist for some groups.

In Dabala, there is a strong negative correlation between satisfaction with life and income for sugarcane growers (Table Q in S2 File). This indicates possibly that increases in income due to involvement in sugarcane do not necessarily translate into gains in subjective wellbeing. For example as seen in Table 12, sugarcane growers report low satisfaction with life, worthwhileness and happiness, at 69%, 51% and 53% respectively, as well as moderate and very high levels of anxiousness (55%). On the other hand, the control group in Dabala exhibited a strong positive correlation (albeit small) between consumption levels and satisfaction with life (Table R in S2 File).

In Yeji, there are strong (albeit small) correlation between consumption, and two indicators of subjective wellbeing (i.e. satisfaction with life, happiness) for permanent jatropha workers (Table S in S2 File). Similar types of correlation can be observed for the control group (Table T in S2 File). However, no strong correlations between indicators of objective and subjective wellbeing are observed for the seasonal plantation workers (Table U in S2 File).

In Kwae, very few strong correlations exist between indicators of objective and subjective wellbeing (Tables V-Y in S2 File). Only for GOPDC workers and independent smallholders there are signs of strong (albeit small) correlations between some indicators of objective and subjective wellbeing (Tables V-W in S2 File).

The above suggests that the different indicators tend to highlight very different aspects of human wellbeing for these groups. For this reason, it becomes necessary to consider them collectively in order to understand better the human wellbeing outcomes of involvement in industrial crop production.

## Discussion

### Patterns between groups in each site

In the sugarcane site (Dabala), sugarcane smallholders have lower levels of multi-dimensional poverty, and higher levels of income and consumption than the control group (see “Income and expenditure” and “Multi-dimensional poverty”). This is consistent with recent studies in other SSA countries that have reported relatively higher income levels and lower poverty levels for sugarcane smallholders [22,81–83]. We also observe that the income obtained through food crop farming is much lower than that received from sugarcane cultivation (see “Basic household characteristics”). This suggests that income gains from involvement in sugarcane production cannot be offset from other locally available livelihoods options such as food crop farming, especially when taking into account the relatively similar sizes of agricultural land between the two groups (Tables 5 and 6).

However, the higher levels of objective wellbeing of sugarcane growers do not necessarily translate in higher levels of subjective wellbeing. Indeed most sugarcane smallholders have reported low levels of satisfaction with life, worthwhileness, and happiness (Table 13, Fig 4). A possible reason might be the uncertainty of finding stable markets to sell their harvest, as well as the perceived impacts of climate variability (e.g. floods, droughts) on sugarcane production. For example, in contrast to oil palm growers in Kwae that have access to multiple market options such as the GOPDC mill and individual small-scale processors (see below), sugarcane farmers in Dabala have much more limited and stable market options. In particular they rely on individual small-scale processors scattered in the Dabala area. Furthermore, during the household survey and FGDs many sugarcane smallholders reported that a flood in 2015 devastated their sugarcane production for that year, leading to massive sugarcane loss. These limited market options and climatic variability seem to cause serious concerns to sugarcane smallholders over the stability of their livelihoods, possibly contributing to the low levels of subjective wellbeing indicators.

In Yeji (jatropha), the control group has lower poverty (in terms of MPI) and higher mean income than jatropha workers. The relatively higher poverty of jatropha workers might reflect the fact that by the time of the survey they were employed only for 2–5 years, which is possibly a short period of time for strong poverty alleviation effects to materialize, especially those related to some of the longer-term MPI components (e.g. education, assets, child mortality). However, the PSM analysis suggests that engagement in jatropha production increases income for permanent workers, which means that such longer-term MPI indicators might improve in the future. Our results contrast a recent study that found lower levels of multi-dimensional poverty for permanent jatropha plantation workers in Mozambique [22], but confirms to some extent the better performance for some individual indicators, especially those related to access to social services [3].

The relatively lower human wellbeing levels of jatropha workers is also reflected in the subjective wellbeing indicators, as a notable proportion of control group respondents reported higher satisfaction with life, happiness and worthwhileness compared to jatropha workers (Table 13, Fig 4). However, interestingly, control group respondents tend to also report higher levels of anxiousness. This might indicate the fact that the low (but stable) plantation salaries are very much appreciated in an area with few formal employment opportunities, as they can provide a buffer against livelihood risks [15].

In Kwae (oil palm), we observed lower MPI levels for GOPDC workers compared to other groups (see “Multi-dimensional poverty”), despite their relatively lower mean income amongst study groups (see “Income and expenditure”). This discrepancy is largely due to the fact that their employment entitles them to some social services offered by GOPDC such as access to piped water, electricity, a hospital and a school. Similar findings have been reported for workers in other types of plantations in Mozambique (jatropha), and Malawi/Swaziland (sugarcane) [22].

Our study also confirms other studies in Ghana (and elsewhere in SSA), which identify oil palm income as a major livelihood source for smallholders and outgrowers [84–86]. However, the actual outcome of engagement in oil palm cultivation on income generation is not clear-cut between analyses. While there is no significant mean income difference between oil palm outgrowers and independent smallholders, their income is significantly higher than that of plantation workers and control groups (Tables 7 and 8). However the PSM analysis suggests that engagement in oil palm cultivation (both as outgrowers and independent smallholders) has a generally negative (but not statistically significant) effect on income (Table 9). As discussed below, some of the diverging human wellbeing outcomes of engagement in oil palm cultivation can be possibly explained by three interrelated factors: (a) agronomy of oil palm, (b) land access/profitability, and (c) market options.

First, due to its size and spacing requirement, oil palms need extensive land areas in order to generate large economic output (i.e. oil palm production benefits from economies of scale). Indeed oil palm producers have on average much larger plots compared to control groups and workers (Tables 5 and 6). GOPDC provides land to outgrowers and requires them to sell their oil palm solely to the GOPDC for the following 20 years. On the contrary, independent smallholders produce oil palm on their private land, which is usually bought over time and/or inherited over generations. This implies that households with higher land endowment have a competitive advantage to engage in independent oil palm production. Thus it might be the case that oil palm production becomes less lucrative in smaller plots, such as those owned by control group respondents (4.9 ha on average, Table 5).

Second, market prices can have a strong effect on the income obtained from oil palm production. Even though the GOPDC is the main oil palm market option in Kwae, there are multiple other informal buyers operating in the area (see “Study sites”). Outgrowers are contractually obliged to sell their harvest to the GOPDC, but independent smallholders can decide where to sell according to price signals. Generally speaking, GOPDC offers a slightly lower price for fresh fruit bunches, compared to informal buyers (respectively GHC 480 and 520 per tone of fresh bunches during the time of survey). This ability to tap into better market options might explain the relatively higher mean income (Table 7) and more favourable comparison to control group for the independent smallholders (Table 9). Another possible explanation for the poor performance of oil palm growers (Table 9) might be the slump in palm oil prices in the period preceding our survey. The prices offered by GOPDC fell constantly from GHC 600 per tonne of fresh bunches (in 2013) to GHC 550 (2014), GHC 500 (2015) and GHC 480 at the time of survey (2016). During the writing of this paper prices were at GHC 450 (February 2019) (personal communication, GOPDC Public Relations Officer, February 2019). Considering the above, even though the generally stable access to markets allows oil palm outgrowers and independent smallholders to capitalize on their production [7,87,88], such price variation might have affected substantially the overall income eventually ending up in their households. When considering the lower prices offered by GOPDC, coupled with the slightly lower mean income and larger cultivated land of outgrowers compared to individual smallholders (Tables 6 and 7), it can be argued that outgrowers might not make the most out of their land and labour.

### Patterns between sites

As outlined in the “Introduction” and “Methodology”, the main group comparison in this paper are performed for groups within the same site. This is because it is difficult to compare and generalize results between sites, due to the pre-existing differences and the specific socioeconomic/environmental contexts in each site. However, we can identify three interesting patterns between sites:

- levels of objective wellbeing decline when moving on a wet-dry gradient (mean scores);
- industrial crop outgrowers, smallholders and independent smallholders are generally better off than other groups in their respective sites (mean scores), but involvement in industrial crop production does not necessarily bring human wellbeing benefits (PSM analysis);
- plantation workers are either worse off or have similar level of human wellbeing with control groups in their respective sites (mean scores), but engagement in plantation work sometimes brings human wellbeing benefits (PSM analysis).

Regarding (a), mean incomes in the oil palm area (Kwae) are generally higher than in the sugarcane (Dabala) and jatropha area (Yeji), both for groups involved in industrial crop production and control groups (Table 7). In particular, control groups in the rainforest zone (Kwae), have higher mean incomes followed by control groups in semi-deciduous (Dabala) and savanna (Yeji) areas. Similar patterns among control groups are also evident (but to a lesser extent) for multi-dimensional poverty and consumption (Tables 7 and 9). These patterns confirm other studies that have identified a wet-dry gradient for income and poverty in West Africa, with increasing poverty levels from rainforest areas (wet regions) to the dry savannas and semi-arid interior [89–91]. Furthermore these patterns are consistent with income inequality levels between the different regions of Ghana [40].

Regarding (b) and (c), when looking at the mean income and consumption levels, the outgrowers and smallholders are better off than other study groups in each site, while plantation workers are either worse off or have the same levels compared to their respective control groups (Tables 7 and 8). However, these patterns are not always visible for some indicators and tests. For example, the results of the PSM analysis for jatropha workers in Yeji and oil palm growers in Kwae suggest that involvement in industrial crop production brings benefits in the first case and disadvantages in the latter (see “Patterns between groups in each site”). Furthermore, when looking at multi-dimensional poverty, workers (especially in Kwae) tend to benefit from some social services offered by the GOPDC and Smart Oil (Tables 11 and 12). Despite the fact that most workers were employed for a small period of time (e.g. permanent jatropha workers have been employed for less than 5 years), they have significantly better MPI levels compared to control groups.

However, it is worth noting that it is highly possible that these multidimensional poverty benefits are precarious. For example, the access to social services might cease (taking a toll on MPI scores), once plantation employment is lost either through termination or company collapse [22]. This is particularly important to consider for industrial crop investments such as jatropha that are prone to collapse; for an extensive discussion of jatropha collapse in Ghana refer to [33].

### Patterns between different indicators of wellbeing

As already discussed above there is good correlation between objective wellbeing indicators such as income and consumption (see “Correlations between indicators of objective and subjective wellbeing”). However, the levels of these indicators do not always correspond with MPI, due to the fact that some of the social benefits that plantation workers receive affect “disproportionally” overall MPI levels (see “Multi-dimensional poverty”).

There are only a few strong correlations between indicators of objective and subjective wellbeing (see “Correlations between indicators of objective and subjective wellbeing”), but it is not clear from our cross-sectional data why this happens. In theory larger income can help households attain greater material welfare thereby contributing positively to subjective wellbeing [23–26]. However this is not evident for most study groups, and contrasts other studies in SSA that have reported the positive correlation between income and subjective wellbeing [25,92], and how enhanced economic security can enhance happiness [93].

At the group level, relatively high incomes with low correlation with subjective wellbeing indicators might indicate "frustrated achievers" [94]. In our study this might be reflect that the higher income and consumption from engagement in industrial crop activities can be precarious, not allowing the high-achieving households enjoy psychologically the benefits. For example, open-ended survey questions and FGDs have suggested that plantation workers are often concerned about job security and overall wages, while smallholders are concerned about market stability and whether industrial crop prices represent a good return on their land/labour investment (see below).

When it comes to job security, plantation employment is among the few formal employment opportunities in poor rural areas of Ghana [15]. However, during the FGDs and household surveys some workers in Kwae and Yeji complained about certain employment practices such as the low salaries, unjustified firing practices, and lack (or low) social security, insurance and paid sick leave. Several respondents also complained about an apparent lack of gender sensitivity in some employment practices. Thus, even though most permanent workers might have a relatively stable salary, these employment conditions might increase job dissatisfaction and give rise to a general feeling of job insecurity, which can possibly influence the low self-reported levels of subjective wellbeing. In this sense improving employment practices can possibly catalyse positive subjective wellbeing outcomes from plantation employment (see “Policy implications and study caveats”).

Similarly, most industrial crop smallholders rely on loans to purchase agricultural inputs such as fertilizers and pesticides. FGDs have suggested that most smallholders enter loan agreements before knowing the actual crop price, as this is established post-harvest depending on many different factors. In fact there is a significant risk of defaulting loan repayments, especially considering yield variability, price volatility, market access (in Dabala), and climatic variability (e.g. droughts and floods in Dabala) (see previous section). Many FGD respondents actually suggested that such factors could cause substantial stress to many famers, whose families depend on industrial crops, possibly influencing their self-reported levels of subjective wellbeing.

Finally, the general lack of strong correlation between objective and subjective wellbeing measures suggests that the different indicators can indeed shed light to different aspects of human wellbeing. Thus, studies that seek to provide a comprehensive understanding of whether different agricultural interventions can improve human wellbeing should contain a combination of different human wellbeing indicators.

### Policy implications and study caveats

Our study identifies two important aspects that need to be considered in industrial crop policies and practices in Ghana, and elsewhere in SSA: (a) securing stable markets for smallholders; (b) improving plantation performance for workers and surrounding communities.

First, stable markets are a strong pre-condition that can allow smallholders reap the benefits of involvement in industrial crop production. This aspect came out strongly in the open-ended survey questions and FGDs in Kwae and Dabala, and is reflected in some of the objective and subjective wellbeing indicator patterns (see previous sections). As already discussed, there are multiple market options in Kwae for oil palm, which allows independent smallholders to choose the most competitive option for selling their produce. This is possibly reflected in their better performance compared to the outgrowers, in terms of higher mean income and better comparison to the control group in the PSM analysis (see “Patterns between groups in each site”), whose only market option is GOPDC. However, the opposite is observed in Dabala, where sugarcane growers often indicate that the lack of stable markets is a key risk of the engagement in sugarcane production. With a pretty much collapsed sugarcane sector in Ghana, stable market development should become a key priority in future efforts to revamp sugarcane production in the country. The government adopted recently a sugarcane policy in its efforts to boost the sector [34], including measures to create market options for sugarcane smallholders such as revitalizing the collapsed sugarcane mills (e.g. the Komenda Sugar Factory). However, more attention should be paid on how to strengthen market development in the areas earmarked and/or targeted for sugarcane development.

Second, commercial agriculture has been promoted in Ghana during the past decade mainly through plantation-based large-scale land acquisitions for jatropha production [95]. Despite the widespread jatropha collapse, there is a renewed interest in oil palm and sugarcane expansion [96]. While such investments can become a substantial source of economic investment (including foreign direct investments, FDIs), it is not clear how appropriate they are for achieving wider rural development and human wellbeing benefits. While plantations can generate rural employment in areas with little formal employment opportunities, these are confined to relatively small portions of the local communities [32,97,98]. Indeed as discussed throughout this paper, such benefits have not always materialized in reality (see “Patterns between groups in each site” and “Patterns between sites”). Yet, the promise of better livelihood opportunities was a major selling point to persuade local communities accept such projects [32]. Such unmet rural development expectations [15] have occasionally catalyzed conflicts between local communities and companies in different parts of the country, leading to the collapse of many industrial crop projects [32]. Policy-makers who support the further promotion of large-scale plantation-based investments need to consider that the eventual generated incomes to workers (and the broader local communities) might be lower than expected.

In this context, some of the possible measures to ensure the delivery of broader sustainability benefits from industrial crop plantations could be: (a) to improve the working conditions of plantation workers, and (b) ensure that the wider community benefits from the infrastructure and social services developed by plantations, even following company restructuring or collapse. In particular, the adoption of sustainability standards can possibly catalyze: (a) improvements in income levels and broader working conditions for plantation workers, (b) development of social services [99]. GOPDC achieved RSPO certification in late 2014 –early 2015. This included the adoption of good production practices to improve both working conditions, as well as the company’s overall sustainability performance. However, given the short period of time between the certification process and our survey, it is doubtful that strong effects would have materialized. To our best knowledge, Smart Oil has not achieved or actively sought any certification. However the long-term compliance and enforcement of such standards (and the evaluation of the outcomes) would be critical to ensure the long-term delivery of these benefits, which is not always guaranteed [100,101]. Additionally, the development of well-thought and socially acceptable exit strategies could safeguard the long-term delivery of social services in the face of company restructuring/collapse [102]. However, the development of such strategies could be an unrealistic expectation in developing countries such as Ghana [103].

Finally, despite the rich information generated presented in the previous sections, establishing causality has been a major challenge. In other words it has been very challenging to ascertain whether those households that are better off in terms of income, consumption and MPI got better off due to their involvement in industrial crop production, and were not already better off. The inability to adopt a random sampling approach and the lack of a baseline study with longitudinal data curtails our ability to definitively conclude this with certainty [22,104]. To compensate for this we have adopted an elaborate purposive sampling and statistical analysis approach to establish causality. We believe that the randomization processes are individually robust to ensure the effective randomization of respondents within each study group [41], even though it was not possible to follow the same sampling and randomization process among all study groups due to the different operational characteristics between industrial crop projects the unique socioeconomic/environmental context of each locality (e.g. population density), and the information available to the research team.

## Conclusion

This study investigated the human wellbeing outcomes of involvement in industrial crop production using evidence from multiple sites, and objective and subjective wellbeing measures. In general, the results suggest that sugarcane and oil palm growers are better off compared to their respective control groups in terms of lower multidimensional poverty, and higher mean income and consumption. However, the PSM analysis suggests that engagement in industrial crop cultivation might not actually deliver positive human wellbeing outcomes in the oil palm study site (i.e. for oil palm outgrowers and independent smallholders). On the other hand, results for workers in oil palm and jatropha plantations are mixed. Workers have consistently lower (or similar) mean income and consumption than their respective control groups. However, the PSM analysis suggests that employment in the jatropha plantation leads to higher income, while employment in oil palm plantations has the exactly opposite effect. Interestingly oil palm workers have the lowest levels of multidimensional poverty than any other groups in Kwae, which possibly reflects their access to the social services offered by the GOPDC.

It is particularly interesting to point out that for most groups there is no strong correlation between the different measures of objective and subjective wellbeing. It is highly possible that diverse factors such as job/market insecurity, climate variability (e.g. floods) and self-comparisons with neighbors could influence the levels of reported subjective wellbeing. This suggests that the different metrics used in this study capture indeed different aspects of human wellbeing. It is therefore important to combine such metrics in order to obtain a more holistic overview of the performance of agricultural investments in SSA. Such a multi-dimensional approach could offer decision-makers and practitioners with more insights as to the potential of different rural development interventions in the continent.

## Supporting information

S1 File Jatropha survey. (DOCX)

S2 File Supplementary material. (DOCX)

S3 File Data. (XLSX)

## References

[1] Wiggins S, Henley G, Keats S. Competitive or complementary? Industrial crops and food security in sub-Saharan Africa. London; 2015.

[2] Gasparatos A, von Maltitz GP, Johnson FX, Lee L, Mathai M, Puppim de Oliveira JA, et al Biofuels in sub-Sahara Africa: Drivers, impacts and priority policy areas. Renew Sustain Energy Rev. 2015;45:879–901.

[3] von Maltitz G, Gasparatos A, Fabricius C. The Rise, Fall and Potential Resilience Benefits of Jatropha in Southern Africa. Sustainability. Multidisciplinary Digital Publishing Institute; 2014 6 5;6(6):3615–43.

[4] Schoneveld G. The geographic and sectoral patterns of large-scale farmland investments in sub-Saharan Africa. Food Policy. 2014;48:34–50.

[5] Tomei J, Helliwell R. Food versus fuel? Going beyond biofuels. Land use policy. 2016;56:320–6.

[6] Dubb A, Scoones I, Woodhouse P. The Political Economy of Sugar in Southern Africa–Introduction. J South Afr Stud. Routledge; 2017 5 4;43(3):447–70.

[7] Carrere R. Oil palm in Africa: Past, present and future scenarios. Montevideo; 2010.

[8] Feintrenie L. Agro-industrial plantations in Central Africa, risks and opportunities. Biodivers Conserv. Springer Netherlands; 2014 6 20;23(6):1577–89.

[9] Ordway EM, Asner GP, Lambin EF. Deforestation risk due to commodity crop expansion in sub-Saharan Africa. Environ Res Lett. IOP Publishing; 2017 4 1;12(4):044015.

[10] Ordway EM, Naylor RL, Nkongho RN, Lambin EF. Oil palm expansion in Cameroon: Insights into sustainability opportunities and challenges in Africa. Glob Environ Chang. Pergamon; 2017 11 1;47:190–200.

[11] Larson DF, Muraoka R, Otsuka K. Why African rural development strategies must depend on small farms. Glob Food Sec. 2016;10:39–51.

[12] Collier P, Dercon S. African Agriculture in 50 Years: Smallholders in a Rapidly Changing World? World Dev. 2014;63:92–101.

[13] Van Vliet JA, Schut AGT, Reidsma P, Descheemaeker K, Slingerland M, Van De Ven GWJ, et al De-mystifying family farming: Features, diversity and trends across the globe. Glob Food Sec. 2015;5:11–8.

[14] Mellor JW, Malik SJ. The Impact of Growth in Small Commercial Farm Productivity on Rural Poverty Reduction. World Dev. 2017;91:1–10.

[15] Schoneveld G, German L a, Nutakor E. Land-based Investments for Rural Development? A Grounded Analysis of the Local Impacts of Biofuel Feedstock Plantations in Ghana. Ecol Soc. 2011;16(4):10-.

[16] Filip O, Janda K, Kristoufek L, Zilbermam D. Food versus fuel: An updated and expanded evidence. Energy Econ. 2017;

[17] Tomei J, Helliwell R. Food versus fuel? Going beyond biofuels. Land use policy. 2015 11;

[18] Kuchler M, Linnér B-O. Challenging the food vs. fuel dilemma: Genealogical analysis of the biofuel discourse pursued by international organizations. Food Policy. 2012 10;37(5):581–8.

[19] Gasparatos A, von Maltitz GP, Johnson FX, Lee L, Mathai M, Puppim de Oliveira JA, et al Biofuels in sub-Sahara Africa: Drivers, impacts and priority policy areas. Renew Sustain Energy Rev. 2015 5;45:879–901.

[20] Balat J, Brambilla I, Porto G. Realizing the gains from trade: Export crops, marketing costs, and poverty. J Int Econ. North-Holland; 2009 6 1;78(1):21–31.

[21] Masanjala WH. Cash crop liberalization and poverty alleviation in Africa: evidence from Malawi. Agric Econ. Blackwell Publishing Inc; 2006 9 1;35(2):231–40.

[22] Mudombi S, Von Maltitz GP, Gasparatos A, Romeu-Dalmau C, Johnson FX, Jumbe C, et al Multi-dimensional poverty effects around operational biofuel projects in Malawi, Mozambique and Swaziland. Biomass and Bioenergy. 2016;

[23] Reyes-García V, Babigumira R, Pyhälä A, Wunder S, Zorondo-Rodríguez F, Angelsen A. Subjective Wellbeing and Income: Empirical Patterns in the Rural Developing World. J Happiness Stud. Springer Netherlands; 2016 4 6;17(2):773–91. 10.1007/s10902-014-9608-2 27642259 PMC5023045

[24] Easterlin RA, McVey LA, Switek M, Sawangfa O, Zweig JS. The happiness-income paradox revisited. Proc Natl Acad Sci U S A. National Academy of Sciences; 2010 12 28;107(52):22463–8. 10.1073/pnas.1015962107 21149705 PMC3012515

[25] Akay A, Martinsson P. Does relative income matter for the very poor? Evidence from rural Ethiopia. Econ Lett. 2011;110:213–5.

[26] Sy I. The subjective approach as a tool for understanding poverty: The case of Senegal. Procedia Econ Financ. 2013;5:336–45.

[27] Kingdon GG, Knight J. Subjective well-being poverty vs. Income poverty and capabilities poverty? J Dev Stud. Routledge; 2006 10;42(7):1199–224.

[28] Battiston D, Cruces G, Lopez-Calva LF, Lugo MA, Santos ME. Income and Beyond: Multidimensional Poverty in Six Latin American Countries. Soc Indic Res. Springer Netherlands; 2013 6 19;112(2):291–314.

[29] Terzi S. How to Integrate Macro and Micro Perspectives: An Example on Human Development and Multidimensional Poverty. Soc Indic Res. Springer Netherlands; 2013 12 25;114(3):935–45.

[30] Wagle UR. The Counting-Based Measurement of Multidimensional Poverty: The Focus on Economic Resources, Inner Capabilities, and Relational Resources in the United States. Soc Indic Res. Springer Netherlands; 2014 1 4;115(1):223–40.

[31] Mitra S. Synergies Among Monetary, Multidimensional and Subjective Poverty: Evidence from Nepal. Soc Indic Res. Springer Netherlands; 2016 1 12;125(1):103–25.

[32] Ahmed A, Campion BB, Gasparatos A. Biofuel development in Ghana: policies of expansion and drivers of failure in the jatropha sector. Renew Sustain Energy Rev. 2017;70:133–49.

[33] Ahmed A, Campion BB, Gasparatos A. Towards a classification of the drivers of jatropha collapse in Ghana elicited from the perceptions of multiple stakeholders. Sustain Sci. Springer Japan; 2018 5 4;1–25.

[34] Ministry of Trade and Industry. Ghana Sugar Policy: Intent and Development Process. Accra; 2016.

[35] Alkire S, Santos ME. Measuring Acute Poverty in the Developing World: Robustness and Scope of the Multidimensional Poverty Index. World Dev. 2014;59:251–74.

[36] OPHI. Measuring Multidimensional Poverty: Insights from Around the World Oxford: Oxford Department of International Development; 2015. 1–16 p.

[37] Augustin R, Fosu K. Growth, inequality, and poverty reduction in developing countries: Recent global evidence. Res Econ. 2016;71:306–36.

[38] Backiny-Yetna P, Steele D, Djima IY. The impact of household food consumption data collection methods on poverty and inequality measures in Niger. 2017;

[39] Ghana Statistical Service. Ghana poverty mapping report. Accra; 2015.

[40] McPherson JM, Sammy J, Sheppard DJ, Mason JJ, Brichieri-Colombi TA, Moehrenschlager A. Integrating traditional knowledge when it appears to conflict with conservation: lessons from the discovery and protection of sitatunga in Ghana. Ecol Soc. The Resilience Alliance; 2016 2 12;21(1):art24.

[41] Gasparatos A, von Maltitz GP, Johnson FX, Romeu-Dalmau C, Jumbe CBL, Ochieng C, et al Survey of local impacts of biofuel crop production and adoption of ethanol stoves in southern Africa. Sci Data. Nature Publishing Group; 2018 9 18;5:180186 10.1038/sdata.2018.186 30226483 PMC6142893

[42] Disney R. Some Measures of Rural Income Distribution in Ethiopia. Dev Change. Blackwell Publishing Ltd; 1976 1 1;7(1):35–44.

[43] Morris SS, Carletto C, Hoddinott J, Christiaensen LJ. Validity of rapid estimates of household wealth and income for health surveys in rural Africa. J Epidemiol Community Health. BMJ Publishing Group Ltd; 2000 5 1;54(5):381–7. 10.1136/jech.54.5.381 10814660 PMC1731675

[44] Davis B, Di Giuseppe S, Zezza A. Are African households (not) leaving agriculture? Patterns of householdsâ€^TM^ income sources in rural Sub-Saharan Africa. Food Policy. 2017;67:153–74. 10.1016/j.foodpol.2016.09.018 28413253 PMC5384437

[45] Tambo JA, Wünscher T. Farmer-led innovations and rural household welfare: Evidence from Ghana. J Rural Stud. 2017;55:263–74.

[46] Bank World. Monitoring Global Poverty Report of the Commission on Global Poverty Monitoring Global Poverty: Report of the Commission on Global Poverty. Washington, DC; 2017.

[47] Weisell R, Dop MC. The Adult Male Equivalent Concept and its Application to Household Consumption and Expenditures Surveys (HCES). Food Nutr Bull. 2012 9 15;33(3_suppl2):S157–62. 23193766 10.1177/15648265120333S203

[48] Haughton J, Khandker SR. Handbook on poverty and inequality 1st ed Washington, DC: World Bank; 2009.

[49] OECD. OECD Guidelines on Measuring Subjective Well-being OECD Publishing; 2013. 24600748

[50] Dehejia R, Wahba S. Propensity Score Matching Methods for Non-experimental Causal Studies. Cambridge, MA; 1998. Report No.: 6829.

[51] Baiyegunhi LJS, Majokweni ZP, Ferrer SRD. Impact of outsourced agricultural extension program on smallholder farmers’ net farm income in Msinga, KwaZulu-Natal, South Africa. Technol Soc. Pergamon; 2018 11 20; 10.1016/j.techsoc.2018.07.007

[52] Chiputwa B, Spielman DJ, Qaim M. Food Standards, Certification, and Poverty among Coffee Farmers in Uganda. World Dev. 2015 2;66:400–12.

[53] Gitonga ZM, De Groote H, Kassie M, Tefera T. Impact of metal silos on households’ maize storage, storage losses and food security: An application of a propensity score matching. Food Policy. Pergamon; 2013 12 1;43:44–55.

[54] Mitiku F, Mey Y De, Nyssen J, Maertens M. Do Private Sustainability Standards Contribute to Income Growth and Poverty Alleviation? A Comparison of Different Coffee Certification Schemes in Ethiopia. Sustainability. MDPI, Open Access Journal; 2017;9(2):1–21.

[55] Hoque MM, Artz GM, Jarboe DH, Martens BJ. Producer Participation in Biomass Markets: Farm Factors, Market Factors, and Correlated Choices. J Agric Appl Econ. 2015;47(3):317–44.

[56] Mendola M. Agricultural technology adoption and poverty reduction: A propensity-score matching analysis for rural Bangladesh. Food Policy. Pergamon; 2007 6 1;32(3):372–93.

[57] Dehejia RH, Wahba S. Propensity Score-Matching Methods for Nonexperimental Causal Studies. Rev Econ Stat. MIT Press 238 Main St., Suite 500, Cambridge, MA 02142–1046 USA journals-info@mit.edu; 2002 2 13;84(1):151–61.

[58] Apiors EK, Suzuki A. Mobile Money, Individuals’ Payments, Remittances, and Investments: Evidence from the Ashanti Region, Ghana. Sustainability. MDPI, Open Access Journal; 2018;10(5):1–26.

[59] Hirano K, Imbens GW. Estimation of Causal Effects using Propensity Score Weighting: An Application to Data on Right Heart Catheterization. Heal Serv Outcomes Res Methodol. Kluwer Academic Publishers; 2001;2(3/4):259–78.

[60] Mwangi JK, Crewett W. The impact of irrigation on small-scale African indigenous vegetable growers’ market access in peri-urban Kenya. Agric Water Manag. Elsevier; 2019 2 1;212:295–305.

[61] Caliendo M, Kopeinig S. SOME PRACTICAL GUIDANCE FOR THE IMPLEMENTATION OF PROPENSITY SCORE MATCHING. J Econ Surv. John Wiley & Sons, Ltd (10.1111); 2008 2 1;22(1):31–72.

[62] Lechner M. Some practical issues in the evaluation of heterogeneous labour market programmes by matching methods. J R Stat Soc Ser A (Statistics Soc. John Wiley & Sons, Ltd (10.1111); 2002 2 1;165(1):59–82.

[63] Rosenbaum PR. Observational Studies. New York: Springer; 2002. 1–17 p.

[64] Alkire S, Foster J. Counting and multidimensional poverty measurement. J Public Econ. North-Holland; 2011 8 1;95(7–8):476–87.

[65] Alkire S, Foster J. Understandings and misunderstandings of multidimensional poverty measurement. J Econ Inequal. Springer US; 2011 6 18;9(2):289–314.

[66] Atkinson AB. Multidimensional Deprivation: Contrasting Social Welfare and Counting Approaches. J Econ Inequal. Kluwer Academic Publishers; 2003;1(1):51–65.

[67] Alkire S, Foster JE, Seth S, Santo ME, Roche JM. Multidimensional poverty measurement and analysis. 4 Counting approaches definitions, origins, and implementations. Oxford Poverty & Human Development Initiative; 2015.

[68] Alkire S, Santos ME. Training material for producing national human development reports: the Multidimensional Poverty Index (MPI): OPHI research in progress 31a. 2011.

[69] Alkire S, Foster JE, Seth S, Santos ME, Roche JM, Ballon P. Multidimensional Poverty Measurement and Analysis: Chapter 5 –the Alkire-Foster Counting Methodology. 2015.

[70] Biewen M. Bootstrap inference for inequality, mobility and poverty measurement. J Econom. North-Holland; 2002 6 1;108(2):317–42.

[71] Alkire S, Foster JE, Seth S, Santos ME, Roche JM, Ballon P. Multidimensional Poverty Measurement and Analysis: Chapter 8 –Robustness Analysis and Statistical Inference. Oxford: Oxford University Press; 2015.

[72] Larsen RJ, Diener E, Emmons RA. An evaluation of subjective well-being measures. Soc Indic Res. Kluwer Academic Publishers; 1985 7;17(1):1–17.

[73] Dolan P, Metcalfe R. Measuring Subjective Wellbeing: Recommendations on Measures for use by National Governments. J Soc Policy. Cambridge University Press; 2012;41(2):409–27.

[74] Duncan OD, Stenbeck M. Are Likert scales unidimensional? Soc Sci Res. Academic Press; 1987 9 1;16(3):245–59.

[75] Gosavi A. Analyzing Responses from Likert Surveys and Risk-adjusted Ranking: A Data Analytics Perspective. Procedia Comput Sci. 2015;61:24–31.

[76] Sullivan GM, Artino AR Jr. Analyzing and interpreting data from likert-type scales. J Grad Med Educ. Accreditation Council for Graduate Medical Education; 2013 12;5(4):541–2. 10.4300/JGME-5-4-18 24454995 PMC3886444

[77] Harpe SE. How to analyze Likert and other rating scale data. Curr Pharm Teach Learn. Elsevier; 2015 11 1;7(6):836–50.

[78] Diener E, Oishi S, Tay L. Advances in subjective well-being research. Nat Hum Behav. Nature Publishing Group; 2018 4 12;2(4):253–60. 10.1038/s41562-018-0307-6 30936533

[79] OECD. OECD Guidelines on Measuring Subjective Well-being. 2013. 24600748

[80] Ghana Statistical Service. Ghana Living Standards Survey (GLSS6). Accra; 2014.

[81] Sulle E. Social Differentiation and the Politics of Land: Sugar Cane Outgrowing in Kilombero, Tanzania. J South Afr Stud. Routledge; 2017 5 4;43(3):517–33.

[82] Matenga CR. Outgrowers and Livelihoods: The Case of Magobbo Smallholder Block Farming in Mazabuka District in Zambia. J South Afr Stud. Routledge; 2017 5 4;43(3):551–66.

[83] Herrmann R, Jumbe C, Bruentrup M, Osabuohien E. Competition between biofuel feedstock and food production: Empirical evidence from sugarcane outgrower settings in Malawi. Biomass and Bioenergy. Pergamon; 2017 9 18;

[84] Osei-Amponsah C, Visser L, Adjei-Nsiah S, Struik PC, Sakyi-Dawson O, Stomph TJ. Processing practices of small-scale palm oil producers in the Kwaebibirem District, Ghana: A diagnostic study. NJAS—Wageningen J Life Sci. Elsevier; 2012 12 1;60–63:49–56.

[85] Awusabo-Asare K, Tanle A. Eking a living: Women entrepreneurship and poverty reduction strategies: The case of palm kernel oil processing in the Central Region of Ghana. Nor Geogr Tidsskr—Nor J Geogr. Taylor & Francis Group; 2008 9;62(3):149–60.

[86] Nchanji YK, Nkongho RN, Mala WA, Levang P. Efficacy of oil palm intercropping by smallholders. Case study in South-West Cameroon. Agrofor Syst. Springer Netherlands; 2016 6 24;90(3):509–19.

[87] Ofosu-Budu K, Sarpong DB. Oil palm industry growth in Africa: A value chain and smallholders’ study for Ghana*. In: Elbehri A, editor. Rebuilding West Africa’s Food Potential. Rome: FAO/IFAD; 2013.

[88] Amanor K, Diderutuah MK. Share contracts in the oil palm and citrus belt of Ghana London: International Institute for Environment and Development; 2001.

[89] Ahmed A, Lawson ET, Mensah A, Gordon C, Padgham J. Adaptation to climate change or non-climatic stressors in semi-arid regions? Evidence of gender differentiation in three agrarian districts of Ghana. Environ Dev. 2016;20:45–58.

[90] Traore PCS, Lamien N, Ayantunde AA, Bayala J, Kalinganire A, Binam JN, et al Sampling the vulnerability reduction-sustainable intensification continuum: a West African paradigm for selection of Dryland Systems sites. Glob Clim Chang its impact food & energy Secur drylands, Proc Elev Int Dryl Dev Conf 18–21 March 2013, Beijing, China. International Dryland Development Commission (IDDC); 2014;186–204.

[91] Lu W, Horlu DSA. Economic well-being of rural farm households in Ghana: A perspective of inequality and polarisation. 2017;

[92] Chin B. Income, health, and well-being in rural Malawi. Demogr Res. NIH Public Access; 2010 11 19;23(35):997–1030. 10.4054/DemRes.2010.23.35 21359133 PMC3045086

[93] Tsai M-C, Dzorgbo D-BS. Familial Reciprocity and Subjective Well-being in Ghana. J Marriage Fam. Wiley/Blackwell (10.1111); 2012 2;74(1):215–28.

[94] Graham C, Pettinato S. Happiness and hardship: opportunity and insecurity in new market economies Washington: Brookings Institution Press; 2002. 174 p.

[95] Ministry of Food and Agriculture. Ghana Commercial Agriculture Project: Environmental and Social Management Framework. Accra; 2011.

[96] Ministry of Food and Agriculture. Master plan study of the Oil palm industry in Ghana. Hampshire: MASDAR; 2011.

[97] Hall R. Land grabbing in Southern Africa: the many faces of the investor rush. Rev Afr Polit Econ. Routledge; 2011 6;38(128):193–214.

[98] Ahmed A, Kuusaana ED, Gasparatos A. The role of chiefs in large-scale land acquisitions for jatropha production in Ghana: insights from agrarian political economy. Land use policy. Pergamon; 2018 6 1;75:570–82.

[99] Brandi C, Cabani T, Hosang C, Schirmbeck S, Westermann L, Wiese H. Sustainability Certification in the Indonesian Palm Oil Sector Benefits and challenges for smallholders. Bonn; 2013.

[100] Morgans CL, Meijaard E, Santika T, Law E, Budiharta S, Ancrenaz M, et al Evaluating the effectiveness of palm oil certification in delivering multiple sustainability objectives. Environ Res Lett. IOP Publishing; 2018 6 1;13(6):064032.

[101] Oya C, Schaefer F, Skalidou D. The effectiveness of agricultural certification in developing countries: A systematic review. World Dev. Pergamon; 2018 12 1;112:282–312.

[102] van Eijck J, Romijn H, Balkema A, Faaij A. Global experience with jatropha cultivation for bioenergy: An assessment of socio-economic and environmental aspects. Renew Sustain Energy Rev. Pergamon; 2014 4 1;32:869–89.

[103] Ahmed A, Jarzebski MP, Gasparatos A. Using the ecosystem service approach to determine whether jatropha projects were located in marginal lands in Ghana: Implications for site selection. Biomass and Bioenergy. Pergamon; 2017 8 18;

[104] von Maltitz GP, Gasparatos A, Fabricius C, Morris A, Willis KJ. Jatropha cultivation in Malawi and Mozambique: impact on ecosystem services, local human well-being, and poverty alleviation. Ecol Soc. The Resilience Alliance; 2016 7 13;21(3):art3.

