# Supplementary material for: Human wellbeing outcomes of involvement in industrial crop production: Evidence from sugarcane, oil palm and jatropha sites in Ghana
Source: PLoS One. 2019 Apr 25;14(4):e0215433. doi: 10.1371/journal.pone.0215433 (PMC6483194; doi:10.1371/journal.pone.0215433)
Supplement: S1 File — (DOCX) [file pone.0215433.s001.docx]

**SUSTAINABILITY ASSESSMENT OF INDUSTRIAL CROPS IN GHANA**

***This questionnaire should be administered only to the head of the household or his wife/husband.***

| **District:** | | | | | **Community:** | | |
| --- | --- | --- | --- | --- | --- | --- | --- |
| **Household ID** | 1 | **Date of interview** | 2 | **Name interviewer** | | 3 | **GPS**  E:  S: |
|  |  |  |  |  | |  |  |
| **Start time:** | | | | **End time:** | | | |

***Category of Respondent (Be careful of mutual exclusiveness, Circle only One of the following)***

| 1. **Permanent worker** | 1. **Seasonal worker** | 1. **Control group** |
| --- | --- | --- |

**SECTION A: DEMOGRAPHIC PROFILES**

*The table below should capture key information on the respondent. Ask questions on age, sex, and educational level of the respondent as well as headship of the household, and fill in/circle the appropriate response.*

| 1 | 2 | 3 | 4 | | 5 | | 6 |
| --- | --- | --- | --- | --- | --- | --- | --- |
| Age of respondent | Sex of respondent | Are you the head of the household? | Relation to household head | | Highest level of education attained | | Total number of years in school |
| *_________*  *Years* | *1=Male*  *2=Female* | *1=Yes*  *2=No* | *1=Head*  *2= Wife or husband* | | *1=No formal schooling*  *2=Some Basic schooling*  *3=Completed Basic schooling*  *4=Some secondary schooling*  *5=Completed secondary school or equivalent*  *6=Completed college/ pre-university /university*  *7=Completed post-graduate* | | ___________ |
| 7. What is the religion of the head of the household? *Circle?*  *1=Christian*  *2=Muslim*  *3=Traditional*  *4= No religion*  *5= Other (Specify)* _____________ | | | | 8. What is your ethnic group  *1=Akans 6=Ga-Adangbe*  *2=Ewe 7=Others________________*  *3=Mole-Dagbon*  *4=Ashanti*  *5=Fante* | | | |
| 9. Were you born in this area?  *1=Yes*  *2=No* | | | | 10. If No, did you move here less than 10 years ago?  *1=Yes*  *2=No* | | | |
| 11. If you have moved to this area as an adult, why did you move?  *1=My wife/husband is from here*  *2=They gave me land here*  *3=Employment opportunity with plantation*  *4=Other employment opportunity*  *5=Other reason (Specify)* __________________ | | | | 12. How many people are there in your household?  *1=1-3*  *2=4-6*  *3=7-9*  *4=10-12*  *5=>12* | | | |
| 13. What is this person’s MAIN occupation  *1= Farmer*  *2= Other agricultural laborer*  *3= Artisan (Mason, Mechanic, shoemaker, hairdresser etc)*  *4= Civil Servant (teacher, nurse, doctor, assembly etc)*  *5= Other formal employment (security, fuel attendant etc)*  *6=Self-employed/own business (Petty trading, food selling etc)*  *7=Student/pupil*  *8=Unemployed,*  *9=Retired*  *10=Other (specify)________* | | | | 14. Months per year this person is employed  *1=Full time*  *2=6-11 months/yr*  *3=3-6 months/yr*  *4=Less than 3 month/yr* | | 15 Monthly salary (GH¢)  *16. Range if no absolute value*  *1= < 100*  *2= 100-500*  *3= 501-1000*  *4= 1001-2000*  *5= 2001-3000*  *6= 3001-4000*  *7= 4001-5000*  *8= > 5000* | |

For each member of your household *(not yourself)*, please tell us the following ***(exclude children below 18 years of age)***

17. What is his/her relation to you? *Insert the response in the relevant column in the table below.*

18. What is his/her age? *Insert age (in years) (18.1) or if not known in the range provided (18.2)*

19. Sex. *Insert (1) if male, (2) if female*

20. How many months per year does this person live in your house? *Insert the number of months per year*

21. What is the highest education level he/she has attained?

22. What is the total years of education?

*23.* What is his/her MAIN occupation? *Insert as appropriate.*

24. How many months is he/she **EMPLOYED** every year? *Insert number*

*25.* ***What is her/his monthly salary? (Please insert the exact amount for people willing to provide or skip to range in question 26)***

*26.* What is her/his monthly salary? *Insert as appropriate*

|  | 17 | 18.1 | 18.2 | 19 | 20 | 21 | 22 | 23 | 24 | 25 | 26 |
| --- | --- | --- | --- | --- | --- | --- | --- | --- | --- | --- | --- |
|  | HH-member Relation to respondent  *1=Respondent*  *2=Spouse*  *3 = Child (>18)*  *4 = Brother/Sister*  *5 = Grand Child*  *6 = Other (Specify)_______* | Age  Exact age | Age (years)  1=18-25  2=26-35  3=36-45  4=46-55  5=56-65  6=>65 | Gender  *1=Male*  *2=Female* | Months per year this person lives in the house | Education  *1=No formal schooling*  *2=Some Basic schooling*  *3=Completed Basic schooling*  *4=Some secondary schooling*  *5=Completed secondary school or equivalent*  *6=Completed college/ pre-university /university*  *7=Completed post-graduate* | *Years of Education* | What is this person’s **MAIN** occupation?  *1=Farmer*  *2= Other agricultural laborer*  *3= Artisan (Mason, Mechanic, shoemaker, hairdresser etc)*  *4= Civil Servant*  *5= Other formal employment (security, fuel attendant etc)*  *6=Self-employed/own business (Petty trading, food selling etc)*  *7=Unemployed,*  *8=Retired*  *9=Other (specify)________* | Months per year this person is employed  1=Full time  2=6-11 months/yr  3=3-6 months/yr  4=Less than 3 month/yr | Monthly salary  (exact amount)  (GHC) | Monthly salary (GHC)  1= < 100  2= 100-500  3= 501-1000  4= 1001-2000  5= 2001-3000  6= 3001-4000  7= 4001-5000  8= > 5000 |
| HHM1 |  |  |  |  |  |  |  |  |  |  |  |
| HHM2 |  |  |  |  |  |  |  |  |  |  |  |
| HHM3 |  |  |  |  |  |  |  |  |  |  |  |
| HHM4 |  |  |  |  |  |  |  |  |  |  |  |
| HHM5 |  |  |  |  |  |  |  |  |  |  |  |
| HHM6 |  |  |  |  |  |  |  |  |  |  |  |
| HHM7 |  |  |  |  |  |  |  |  |  |  |  |

27-31. For Household members below 18 years, please provide their details as follows:

|  | 27 | 28 | 29 | 30 | 31 |
| --- | --- | --- | --- | --- | --- |
|  | Age (years) | Gender  *1 = Male*  *2 = Female* | Months per year this person lives in the house | Is this household member still in school?  1=Yes  2=No | *Years of schooling* |
| HHM8 |  |  |  |  |  |
| HHM9 |  |  |  |  |  |
| HHM10 |  |  |  |  |  |
| HHM11 |  |  |  |  |  |
| HHM12 |  |  |  |  |  |
| HHM13 |  |  |  |  |  |
| HHM14 |  |  |  |  |  |

**SECTION B: AGRONOMIC PRACTICES**

*To be administered to* ***ALL RESPONDENTS***

1. **Do you or your household have agricultural land? If Not ask Question 5-6 and then question 19 (Livestock).**

1=Yes

2=No

1. If Yes, for each agricultural plot owned by the household (incl. currently unused or fallow plots) ask the following questions

2.1 How did you acquire this plot? Insert in table below

2.2 What is the size of the plot? Insert in table below size in acres

2.3 How far away is this plot from your household? Insert in table distance in Km.

2.4 What is the main use of the plot? Insert in table below

2.5 What is the secondary use of the plot? Insert in table below

| **2. Plot #** | **2.1 How acquired?**  *1=Inherited from my parents*  *2= Inherited from spouse parents*  *3=Encroached a virgin forest;*  *4=Purchased*  *5=Allocated by chief*  *6=Allocated by government;*  *7=Other (specify)_______* | **2.2 Size**  (Acre) | **2.3 Distance from home**  (km) | **2.4 Main use**  *1=Maize;*  *2=Groundnuts;*  *3=Beans;*  *4=Cassava;*  *5=millet*  *6=sorghum*  *7=Other food crop: Specify*  *8=Sugarcane;*  *9=Jatropha;*  *10=Oil palm*  *11=Cotton*  *12=Tobacco*  *13=Other non-food crop Specify*  *14=Fallow*  *15=Grazing*  *16=Wood/timber*  *17=Other, specify_* | **2.5 Secondary u**se  *1=Maize;*  *2=Groundnuts;*  *3=Beans;*  *4=Cassava;*  *5=millet*  *6=sorghum*  *7=Other food crop: Specify*  *8=Sugarcane;*  *9=Jatropha;*  *10=Oil palm*  *11=Cotton*  *12=Tobacco*  *13=Other non-food crop Specify*  *14=Fallow*  *15=Grazing*  *16=Wood/timber*  *17=Other, specify* |
| --- | --- | --- | --- | --- | --- |
|  |  |  |  |  |  |
|  |  |  |  |  |  |
|  |  |  |  |  |  |
|  |  |  |  |  |  |
|  |  |  |  |  |  |
|  |  |  |  |  |  |

1. In what type of land agreement is your farm? *Circle*

*1= Share cropping (Abunu)*

*2= Share cropping (Abusa)*

*3=Outright purchase*

*4=Family land (Inheritance)*

*5=Other Specify____________*

1. Is any of your household’s cropland **NOT** being cultivated?

*1=Yes*

*2=No*

1. Did your household own more land in the past?

*1=Yes*

*2=No*

1. For lands **Not Cultivated or Formerly Owned** please ask for the size, years, primary use and reason in the table below
   1. What was the size of the plot? *Insert in table below size in acres*
   2. What is the category of land*? Insert “****Not Cultivated but owned” or “Formerly Owned”***
   3. When did you lose access or stopped cultivating it? *Insert in table below date (year)*
   4. What was the main use of the plot? *Insert in table below*
   5. What was the reason that you lost the plot? *Insert in table below*

| 6  Plot # | 6.1  **Size**  (acres) | 6.2  **Category**  1= Not cultivated but owned  2= Formerly Owned | 6.3  **When**  (year) | 6.4  **Primary use**  *1=Grow food crop*  *2=Grow non-food crop*  *3=Fallow*  *4=Grazing*  *5=Wood/timber lot*  *6=Other, specify_* | 6.5  **Reason**  *1=Sold it to get money*  *2=Shortage of money/investment capital to maintain*  *3=Shortage of labor to cultivate it*  *4=Lost it from the jatropha plantation*  *5=Dispute over rights/ownership*  *6=It was not suitable for agriculture so stopped using it*  *7=It was the preference of a member of the household*  *(Specify reason, if known)*  *8=Other reason (Specify* |
| --- | --- | --- | --- | --- | --- |
|  |  |  |  |  |  |
|  |  |  |  |  |  |
|  |  |  |  |  |  |
|  |  |  |  |  |  |
|  |  |  |  |  |  |
|  |  |  |  |  |  |

1. How would you assess your current land compared with the “Formerly Owned Land” in terms of the following?

| - 1. Soil quality   1=Higher soil quality  2=Moderate soil quality  3=Lower soil quality  4=Do not know | - 1. Access to water   1=Better access  2=Moderate access  3=Lower access  4=Do not know | 7.3 Distance from household  1=Further  2=Same distance  3=Closer  4=Do not know | 7.4 Distance from market  1=Further  2=Same distance  3=Closer  4=Do not know |
| --- | --- | --- | --- |

For **LAST SEASON**, fill in the following table in relation to the type of **FOOD CROPS** grown by the household, the amount sold, the cash received

1. Ask **FOOD CROPS** consequently and specify amounts produced and sold for each crop from ALL farmer’ plots in the last season.

In the questions below, (…) denotes the different types of crops.

- 1. Did you grow any (….) in the last season? *Enter response in the table*

If Yes, continue with the following questions

8.2 What was the area under (…) in ALL your plots?

8.3 How much of your plots under (….) was under irrigation?

8.4-8.5 How much (…) did you produce last season? *Insert in table below in local units (if applicable) and calculate in kilos (Kg) last season*

8.6 Did you sell any of the (…) you produced last season?

8.7-8.8 How much (…) did you sell last season? *Insert in table below in local units and calculate in Kg*

8.9 How much money did you receive for the (…) you sold last season? *Insert in table amount received last season*

9.10 Which member of the household received this cash?

| **8**  **Crop** | **8.1**  **Did you grow any last season**  1=Yes  2=No | **8.2**  **Area** (Acre) | **8.3**  **Irrigation**  1=All  2=Most  3=Little  4=None | **8.4**  **Amount produced**  **(local unit)** | **8.5**  **Amount produced**  (KG last season) | **8.6**  **Sold?**  1=Yes  2=No | **8.7**  **Amount sold**  **(local unit)**  **(bag/sack, bucket, pan, olonka),** | **8.8**  **Amount sold**  (KG last season) | **8.9**  **Cash received**  *(GHC)* | **8.10**  **Who received the money**  1=Household head  2=Spouse  3=Other household member (specify)  4=Other person (specify)  5=Shared between family |
| --- | --- | --- | --- | --- | --- | --- | --- | --- | --- | --- |
| Dry Maize |  |  |  |  |  |  |  |  |  |  |
| Green/wet maize |  |  |  |  |  |  |  |  |  |  |
| Rice |  |  |  |  |  |  |  |  |  |  |
| Millet |  |  |  |  |  |  |  |  |  |  |
| Plantain |  |  |  |  |  |  |  |  |  |  |
| Groundnut |  |  |  |  |  |  |  |  |  |  |
| Cassava |  |  |  |  |  |  |  |  |  |  |
| Beans |  |  |  |  |  |  |  |  |  |  |
| Vegetables |  |  |  |  |  |  |  |  |  |  |
| Fruits/berries |  |  |  |  |  |  |  |  |  |  |
| Yam |  |  |  |  |  |  |  |  |  |  |
| Cowpea |  |  |  |  |  |  |  |  |  |  |
| Other  Specify_________ |  |  |  |  |  |  |  |  |  |  |

1. For the **FOOD CROPS you sold last season**, please specify the months you **RECEIVED THE CASH FROM SELLING THEM**. *Ask for each crop sold as indicated in previous Table and tick the appropriate month(s)*

|  | **Jan** | **Feb** | **Mar** | **Apr** | **May** | **Jun** | **Jul** | **Aug** | **Sep** | **Oct** | **Nov** | **Dec** |
| --- | --- | --- | --- | --- | --- | --- | --- | --- | --- | --- | --- | --- |
| Dry Maize |  |  |  |  |  |  |  |  |  |  |  |  |
| Green/wet maize |  |  |  |  |  |  |  |  |  |  |  |  |
| Rice |  |  |  |  |  |  |  |  |  |  |  |  |
| Millet |  |  |  |  |  |  |  |  |  |  |  |  |
| Plantain |  |  |  |  |  |  |  |  |  |  |  |  |
| Groundnut |  |  |  |  |  |  |  |  |  |  |  |  |
| Cassava |  |  |  |  |  |  |  |  |  |  |  |  |
| Beans |  |  |  |  |  |  |  |  |  |  |  |  |
| Vegetables |  |  |  |  |  |  |  |  |  |  |  |  |
| Fruits/berries |  |  |  |  |  |  |  |  |  |  |  |  |
| Yam |  |  |  |  |  |  |  |  |  |  |  |  |
| Cowpea |  |  |  |  |  |  |  |  |  |  |  |  |
| Others______ |  |  |  |  |  |  |  |  |  |  |  |  |
| Others______ |  |  |  |  |  |  |  |  |  |  |  |  |

For **LAST SEASON**, fill in the following table in relation to the type of **NON**-**FOOD CROPS** grown by the household, the amount sold, the cash received

1. Ask **NON-FOOD CROPS** consequently and specify amounts produced and sold for each crop from ALL farmer’ plots in the last season.

In the questions below, (…) denotes the different types of crops.

- 1. Did you grow any (….) in the last season? *Enter response in the table*

*If Yes, continue with the following questions*

10.2 What was the area under (…) in ALL your plots?

10.3 How much of your plots under (….) was under irrigation?

10.4-10.5 How much (…) did you produce last season? *Insert in table below in local units (if applicable) and calculate in kilos (Kg) last season*

10.6 Did you sell any of the (…) you produced last season

10.7-10.8 How much (…) did you sell last season? *Insert in table below in local units and calculate in Kg*

10.9 How much money did you receive for the (…) you sold last season? *Insert in table amount received last season*

10.10 Which member of the household received this cash?

| **10**  **Crop** | **10.1**  **Did you grow any last season**  1=Yes  2=No | **10.2**  **Area** (Acre) | **10.3**  **Irrigation**  1=All  2=Most  3=Little  4=None | **10.4**  **Amount produced**  **(local unit)** | **10.5**  **Amount produced**  (KG last season) | **10.6**  **Sold?**  1=Yes  2=No | **10.7**  **Amount sold**  **(local unit)**  **(bag/sack, bucket, pan, olonka),** | **10.8**  **Amount sold**  (KG last season) | **10.9**  **Cash received**  *(GHC)* | **10.10**  **Who received the money**  1=Household head  2=Spouse  3=Other household member (specify)  4=Other person (specify)  5=Shared between family |
| --- | --- | --- | --- | --- | --- | --- | --- | --- | --- | --- |
| Sugarcane |  |  |  |  |  |  |  |  |  |  |
| Tea |  |  |  | ________Liters  _______ Sticks | ________Liters  _______ Sticks |  |  |  |  |  |
| Cotton |  |  |  |  |  |  |  |  |  |  |
| Tobacco |  |  |  |  |  |  |  |  |  |  |
| Coffee |  |  |  |  |  |  |  |  |  |  |
| Sun flower |  |  |  |  |  |  |  |  |  |  |
| Other_________ |  |  |  |  |  |  |  |  |  |  |

1. For the **NON-FOOD CROPS you sold last season**, please specify the months you **RECEIVED THE CASH FROM SELLING THEM**. *Ask for each crop sold as indicated in previous Table and tick the appropriate month(s)*

|  | **Jan** | **Feb** | **Mar** | **Apr** | **May** | **Jun** | **Jul** | **Aug** | **Sep** | **Oct** | **Nov** | **Dec** |
| --- | --- | --- | --- | --- | --- | --- | --- | --- | --- | --- | --- | --- |
| Sugarcane |  |  |  |  |  |  |  |  |  |  |  |  |
| Tea |  |  |  |  |  |  |  |  |  |  |  |  |
| Cotton |  |  |  |  |  |  |  |  |  |  |  |  |
| Tobacco |  |  |  |  |  |  |  |  |  |  |  |  |
| Coffee |  |  |  |  |  |  |  |  |  |  |  |  |
| Sun flower |  |  |  |  |  |  |  |  |  |  |  |  |

*12. Fill in the following table in relation to the type of agricultural inputs purchased by the household for each major type of crops for the past year.*

*Insert in table below both the volume purchased (in Kg or liters) and the cost for the whole year.* ***(MATERIAL INPUTS)***

| **Crops** | Seeds (kg) | | Pesticides (Lt) | | Herbicides (Lt) | | Weedicides (Lt) | | Fertilizers (kg) | | Others | |
| --- | --- | --- | --- | --- | --- | --- | --- | --- | --- | --- | --- | --- |
|  | Volume | Cost*(GHC)* | Volume | Cost*(GHC)* | Volume | Cost*(GHC)* | Volume | Cost*(GHC)* | Volume | Cost*(GHC)* | Volume | Cost*(GHC)* |
| **Sugarcane** |  |  |  |  |  |  |  |  |  |  |  |  |
| **Tea** |  |  |  |  |  |  |  |  |  |  |  |  |
|  |  |  |  |  |  |  |  |  |  |  |  |  |
| **Cotton** |  |  |  |  |  |  |  |  |  |  |  |  |
| **Tobacco** |  |  |  |  |  |  |  |  |  |  |  |  |
| **All food crops** |  |  |  |  |  |  |  |  |  |  |  |  |

1. Did you hire agricultural labour last season? *Circle*

*1=Yes*

*2=No*

*If yes, please ask the following questions (from 14 and if NO go to question 19)*

1. For which agricultural activity? *Circle*

1=Land preparation

2=Planting

3=Weeding

4=Harvesting

5=Transport

6=Apply agrochemicals

7=Other agricultural activity (Specify)___

| 1. How many people over how many days was it?   ____________ man days | 1. What was the total cost for hired agricultural labour last year?   _________ *(GHC)* |
| --- | --- |

1. Did you use shared labour systems?

*1=Yes*

*2=No*

*If Yes,*

1. What kind of activity did they help you with?

1=Land preparation

2=Planting

3=Weeding

4=Harvesting

5=Transport

6=Apply agrochemicals

7=Other agricultural activity (Specify)___

19.1 **Do you own livestock and poultry?**

19.2 How many animals does your household own?

*For Cattle, Goats, Sheep, Pigs. Beehives add the exact number*

*For Chicken, Pigeons, Guinea Fowls add the code in the table*

19.3 How many did you sell last year?

19.4 How much money did you receive from selling this livestock?

19.5 Which member of the household received the cash from selling this livestock?

19.6 Why did you sell this livestock?

|  | **19.1 Do you own?**  1=Yes  2=No | **19.2 Number owned**  1=< 5  2=5-10;  3=10-20;  4=20-30;  5=30-40;  6=40-50;  7=> 50 | **19.3 Number sold last year** | **19.4 Money received**  (GHC) | **19.5 Who received the money**  1=Household head  2=Spouse  3=Other household member (specify)  5=Shared between members of family  6=Other person (specify) | **19.6 Reason for selling**  1=Meet regular household needs (e.g. food)  2=Meet unexpected household need (e.g. health)  3=Repay debt  4=Dowry  5=Could not maintain them  6=Other (specify) |
| --- | --- | --- | --- | --- | --- | --- |
| **Cattle** |  |  |  |  |  |  |
| **Goats** |  |  |  |  |  |  |
| **Sheep** |  |  |  |  |  |  |
| **Pigs** |  |  |  |  |  |  |
| **Rabbits** |  |  |  |  |  |  |
| **Chickens** |  |  |  |  |  |  |
| **Guinea Fowls** |  |  |  |  |  |  |
| **Pigeons** |  |  |  |  |  |  |
| **Beehives** |  |  |  |  |  |  |
| **Other (Specify)**  **__________** |  |  |  |  |  |  |

**Water Inputs**

20. What is the main source of water for irrigation and livestock? *Insert code*

| **20.1**  **For irrigation (for ALL crops)** | *Categories*  *1 = Piped water to dwelling*  *2= Piped water to plot/yard*  *3= Piped water to public tap/stand-pipe*  *4= Borehole*  *5= Dug well*  *6= Spring*  *7= Rainwater*  *8= River/lake/pond/stream/dam*  *9= Tanker truck*  *10= Other (Specify)* |
| --- | --- |
| **20.2**  **For livestock** |  |

1. On average, how far away and how much do you pay for water per month for the following activities? *Insert amount per month or per year.*

|  | 21.1  Distance (in meters) | 21.2  Time spent per day (in minutes) | 21.3  Cost (per month) (GHC) | 21.4  Cost (per year) (GHC) |
| --- | --- | --- | --- | --- |
| **For irrigation** |  |  |  |  |
| **For livestock** |  |  |  |  |

1. How important is to your household to have water for irrigation? *Circle*
2. Do you have enough water for irrigation? *Circle*
3. How has your access to water for irrigation changed since the company started operating? *Circle*
4. How has your access to water for irrigation changed because of the operations other farmers? *Circle*

| **22 Importance**  *1=Not important*  *2=Low importance*  *3=Medium importance*  *4=High importance* | **23 Quantity**  *1=Always enough*  *2=Sometimes enough*  *3=Usually not enough*  *4=Never enough*  *5=Never irrigated* | **24 Change in access since the community started growing (…)**  *1=Decreased significantly*  *2=Decreased moderately*  *3=Remained the same*  *4=Increased moderately*  *5=Increased significantly* | **25 Change in access due to operations of other farmer**  *1=Decreased significantly*  *2=Decreased moderately*  *3=Remained the same*  *4=Increased moderately*  *5=Increased significantly* |
| --- | --- | --- | --- |

26. Did you receive any extension support in the previous years?

*1=Yes*

*2=No*

If the answer is yes, proceed to questions 27-36 in next page. **If no, proceed to Question 37**

27 Did you receive extension support from any of the following organisations last season? If Yes Add in Table

1. If yes, for which crop did you receive extension support? (use the codes)
2. What type of extension support did you receive last season? Add all appropriate (more than one answers are allowed) (Use the codes)
3. If you received any inputs or farm implements, can you specify the quantity received last season? Put number and description. If more than one implement/input received, then add and explain all
4. Did you have to pay for this extension services last season?
5. If you had to pay, what was the cost last season?
6. What has been the frequency of this extension service in the past 5 years?
7. How would you rate overall the quality of this extension service?

| **Extension**  **Provider** | 27 | 28 | 29 | 30 | 31 | 32 | 33 | 34 |
| --- | --- | --- | --- | --- | --- | --- | --- | --- |
|  | Received extension last season?  1=Yes  2=No | Which crop?^(a)^ | What type of service^(b)^ | Quantity and type of tools / implements | Did you have to pay?  1=Yes  2=No | Cost (GHC) |  |  |
|  |  |  |  |  |  |  | Frequency  1=Rising  2=Remained same  3=Falling, | Quality  1=Very good  2=Good  3=Average  4=Poor  5=Not good  6=Do not know |
| **Government extension service** |  |  |  |  |  |  |  |  |
| **NGO/ Development project** |  |  |  |  |  |  |  |  |
| **Cooperative** |  |  |  |  |  |  |  |  |
| **Large Scale Farmer** |  |  |  |  |  |  |  |  |
| **Private practitioners** |  |  |  |  |  |  |  |  |
| **Agro-Industrial Company** |  |  |  |  |  |  |  |  |
| **Religious group** |  |  |  |  |  |  |  |  |
| **Other (specify)** |  |  |  |  |  |  |  |  |

Codes:

(a): 1=Maize, 2=Groundnuts, 3=Beans, 4=Cassava, 5=millet, 6=Rice, 7=other food crops, 8=Sugarcane, 9=Jatropha, 10=Tobacco, 11=other non-food crops

(b): 1- Spacing, 2- Use of agro-chemicals, 3- Erosion control, 4- Organic fertilizer use, 5- Inorganic fertilizer use, 6- Use of Improved seed, 7- Mechanization practices, 8- Irrigation Technology, 9- Crop storage, 10- Agro-processing, 11- Agro-forestry, 12- Fish farming, 13- Other (specify)

1. Do you think such improvements have affected your yields or production output for the targeted crops?

1=Yes

2=No

1. Please explain your response

___________________________________________________________________________________________________________________

1. Do you belong to any farmers’ association?

1=Yes

2=No

38. If Yes, specify what crop and what activities this association undertakes ________________________________

39. What kind of benefits do you get? *Tick all that is applicable*

| **Benefit** | **Tick** | **Benefit** | **Tick** |
| --- | --- | --- | --- |
| Access to farm inputs |  | Dispute settlement |  |
| Access to training |  | Collective bargaining |  |
| Sharing of labour |  | Price setting and marketing |  |
| Savings and loans |  | Others______________________ |  |

**SECTION C: RELIANCE ON ENVIRONMENTAL RESOURCES**

*To be administered to all respondents*

1. Do you obtain any of the following goods for **FREE** from nature? *Read through the list in the table below and enter response*

*If Yes, ask following questions*

1. How important is/are these **FREE** goods provided by nature to your household? *Insert in table below, for each good*
2. How often do/did you take (…) from nature? *Insert in table below*
3. Why do you collect these **FREE** goods from nature?
4. How much money did you receive for selling these **FREE** products from nature?

|  | **1.**  1=Yes  2=No | | **2. Importance**  *1=Not important*  *2=Low importance*  *3=Medium importance*  *4=High importance* | | **3. Periodicity**  *1=Every day*  *2=A few times/week*  *3=A few times/month*  *4=A few times/year*  *5=Never* | | **4 Reason for collection**  *1=Mainly for own household consumption*  *2=Mainly for selling*  *3=Both for own consumption and selling* | | **5 Money received**  (local currency) | |
| --- | --- | --- | --- | --- | --- | --- | --- | --- | --- | --- |
| **Wood fuel** |  |  |  |  |  |  |  |  |  |  |
| **Charcoal** |  |  |  |  |  |  |  |  |  |  |
| **Timber/grass for construction** |  |  |  |  |  |  |  |  |  |  |
| **Medicinal plants** |  |  |  |  |  |  |  |  |  |  |
| **Honey** |  |  |  |  |  |  |  |  |  |  |
| **Wild fruits** |  |  |  |  |  |  |  |  |  |  |
| **Mushrooms** |  |  |  |  |  |  |  |  |  |  |
| **Wild animals for food (bush meat)** |  |  |  |  |  |  |  |  |  |  |
| **Other edible products from forest**  **(Specify)__________** |  |  |  |  |  |  |  |  |  |  |
| **Fish** |  |  |  |  |  |  |  |  |  |  |
| **Grazing of animals** |  |  |  |  |  |  |  |  |  |  |
| **Other 1 (Specify)**  **_________________** |  |  |  |  |  |  |  |  |  |  |
| **Other 2 (Specify)**  **_________________** |  |  |  |  |  |  |  |  |  |  |

6. For the resources you get **FREE** from nature, please specify the months you collect them? *Tick appropriate month*

|  | **Jan** | **Feb** | **Mar** | **Apr** | **May** | **Jun** | **Jul** | **Aug** | **Sep** | **Oct** | **Nov** | **Dec** |
| --- | --- | --- | --- | --- | --- | --- | --- | --- | --- | --- | --- | --- |
| **Wood fuel** |  |  |  |  |  |  |  |  |  |  |  |  |
| **Charcoal** |  |  |  |  |  |  |  |  |  |  |  |  |
| **Timber/grass for construction** |  |  |  |  |  |  |  |  |  |  |  |  |
| **Medicinal plants** |  |  |  |  |  |  |  |  |  |  |  |  |
| **Honey** |  |  |  |  |  |  |  |  |  |  |  |  |
| **Wild fruits** |  |  |  |  |  |  |  |  |  |  |  |  |
| **Mushrooms** |  |  |  |  |  |  |  |  |  |  |  |  |
| **Wild animals for food (bush meat)** |  |  |  |  |  |  |  |  |  |  |  |  |
| **Other edible products from forest**  **(Specify)__________** |  |  |  |  |  |  |  |  |  |  |  |  |
| **Fish** |  |  |  |  |  |  |  |  |  |  |  |  |
| **Grazing of animals** |  |  |  |  |  |  |  |  |  |  |  |  |
| **Other 1 (Specify)**  **_________________** |  |  |  |  |  |  |  |  |  |  |  |  |
| **Other 2 (Specify)**  **_________________** |  |  |  |  |  |  |  |  |  |  |  |  |

7. How has your access to these **FREE** resources changed since the company started operation?

8. How has your access to these **FREE** resources changed because of the plantation operations

|  | **7**  **Since the community started growing**  *1=Decreased significantly*  *2=Decreased moderately*  *3=Remained the same*  *4=Increased moderately*  *5=Increased significantly* | | **8**  **Due to plantation operations**  *1=Decreased significantly*  *2=Decreased moderately*  *3=Remained the same*  *4=Increased moderately*  *5=Increased significantly* | |
| --- | --- | --- | --- | --- |
| **Wood fuel** |  |  |  |  |
| **Charcoal** |  |  |  |  |
| **Timber/grass for construction** |  |  |  |  |
| **Medicinal plants** |  |  |  |  |
| **Honey** |  |  |  |  |
| **Wild fruits** |  |  |  |  |
| **Mushrooms** |  |  |  |  |
| **Wild animals for food (bush meat)** |  |  |  |  |
| **Other edible products from forest**  **(Specify)__________** |  |  |  |  |
| **Fish** |  |  |  |  |
| **Grazing of animals** |  |  |  |  |
| **Other 1 (Specify)**  **_________________** |  |  |  |  |
| **Other 2 (Specify)**  **_________________** |  |  |  |  |

**SECTION D: FOOD SECURITY**

***To be administered to all respondents***

1. How many meals does your household usually eat per day? ________ meals
2. Could you tell me how many days in the past 7days your household has eaten the following foods? *Ask consequently each food category. Insert 0-7 in the table below*
3. What is the primary source of each food item you ate this week? *Insert responses in the table below*
4. What is the second main source of food (if any)? *Insert responses in table below as specified*

|  | 2. Days eaten in past 7 days  (0-7) | 3. Primary source  1=Own production  2=Purchase  3=From environment  4=Borrowed  5=Received as gift  6=Food Aid  7=Other, specify | 4. Secondary source  1=Own production  2=Purchase  3=From environment  4=Borrowed  5=Received as gift  6=Food Aid  7=Other, specify |
| --- | --- | --- | --- |
| Maize |  |  |  |
| Rice |  |  |  |
| Bread/wheat/other cereals |  |  |  |
| Tubers (cassava/potatoes/sweet potatoes/yams/etc.) |  |  |  |
| Groundnuts, beans and peas |  |  |  |
| Fish |  |  |  |
| Meat from livestock |  |  |  |
| Meat from poultry |  |  |  |
| Vegetable oils/fats |  |  |  |
| Eggs |  |  |  |
| Milk and dairy products |  |  |  |
| Vegetables (incl leaves) |  |  |  |
| Fruits |  |  |  |
| Sugar and sweets |  |  |  |

***Note: Please remember to cross-check with Section C***

***if primary/secondary source is 1-Own production***

***if primary/secondary source is 2-Purchase***

***if primary/secondary source is 3-From environment***

*For each of the questions in the following table first ask the occurrence question – that is, whether the condition in the question happened at all in the past four weeks (yes or no). If the respondent answers “yes” to an occurrence question, then ask the frequency-of-occurrence question to determine whether the condition happened rarely (once or twice), sometimes (three to ten times) or often (more than ten times) in the past four weeks.*

*Example:*

*5.1 In the past four weeks, did you worry that your household would not have enough food?*

*1 = Yes (go to 5.2)*

*2 = No (skip to Question 6)*

*5.2 How often did this happen?*

|  | 5.1  1= Yes  2 = No | 5.2  How often did this happen?  1 = Rarely (once or twice)  2 = Sometimes (three to ten times in the past four weeks)  3 = Often (more than ten times in the past four weeks) |
| --- | --- | --- |
| In the past four weeks, did you **worry that your household would not have enough food?** |  |  |
| In the past four weeks, were **you or any household member not able to eat the kinds of foods you preferred** because of a lack of resources? |  |  |
| In the past four weeks, **did you or any household member have to eat a limited variety of foods** due to a lack of resources? |  |  |
| In the past four weeks, **did you or any household member have to eat some foods that you really did not want to eat** because of a lack of resources? |  |  |
| In the past four weeks, **did you or any household member have to eat a smaller meal than you felt you needed** because there was not enough food? |  |  |
| In the past four weeks, **did you or any other household member have to eat fewer meals in a day** because there was not enough food? |  |  |
| In the past four weeks, **was there ever no food to eat of any kind in your household** because of lack of resources to get food? |  |  |
| In the past four weeks, **did you or any household member go to sleep at night hungry** because there was not enough food? |  |  |
| In the past four weeks, **did you or any household member go a whole day and night without eating anything** because there was not enough food? |  |  |

1. How many months in the year did you or any household member go to sleep at night hungry because there was not enough food? _________
2. During which months does your household not have enough food? *Tick relevant month*

| **Jan** | **Feb** | **Mar** | **Apr** | **May** | **Jun** | **Jul** | **Aug** | **Sep** | **Oct** | **Nov** | **Dec** |
| --- | --- | --- | --- | --- | --- | --- | --- | --- | --- | --- | --- |
|  |  |  |  |  |  |  |  |  |  |  |  |

1. In the past 7 days, if there have been times when you did not have enough food or money to buy food, how many days has your household had to?

*Use numbers 0 – 7 to answer number of days; Use NA for not applicable*

| **Behavior** | **8. Frequency** |
| --- | --- |
| Rely on less preferred and less expensive foods? |  |
| Borrow food, or rely on help from a friend or relative? |  |
| Purchased on credit? |  |
| Gather wild food, hunt, or harvest immature crops? |  |
| Consume seed stock held for next season? |  |
| Send household members to eat elsewhere? |  |
| Send household members to beg? |  |
| Limit portion size at meal times? |  |
| Restrict consumption by adults in order for small children to eat? |  |
| Feed working members of HH at the expense of non-working members? |  |
| Reduce number of meals eaten in a day? |  |
| Skip entire day without eating? |  |
| Borrow money to buy food |  |
| Casual labour for food (as payment) |  |
| Food Aid |  |

1. **FOOD PURCHASE** Did your household purchase the following?

*For LAST SEASON, fill in the following table in relation to the type of* ***FOOD CROPS*** ***BOUGHT*** *by the household.*

*We need the amount bought, the cash spent, and the reason for purchasing.*

9.1 Did you buy any (…) last season? *If yes, continue with the following questions*

9.2-9.3 How much did you buy?

*Insert in table below amount bought in local units and calculate in Kg for the entire season*

9.4 How much did it cost?

9.5 What was the reason buying this (…)?

| **Crop** | **9.1 Did you purchase any last season**  1=Yes  2=No | **9.2 Amount purchased**  (local unit) | **9.3 Amount purchased**  (KG last season) | **9.4 Cost**  (Local currency | **9.5 Reason for buying**  1=Food  2=Re-sell  3=Other (specify) |
| --- | --- | --- | --- | --- | --- |
| Dry Maize |  |  |  |  |  |
| Green/wet maize |  |  |  |  |  |
| Rice |  |  |  |  |  |
| Millet |  |  |  |  |  |
| Plantain |  |  |  |  |  |
| Groundnut |  |  |  |  |  |
| Cassava |  |  |  |  |  |
| Beans |  |  |  |  |  |
| Vegetables |  |  |  |  |  |
| Fruits/berries |  |  |  |  |  |
| Yam |  |  |  |  |  |
| Cowpea |  |  |  |  |  |
| Other __________ |  |  |  |  |  |

**SECTION E: LIVELIHOOD, INCOME AND ASSETS**

*To be administered to all respondents*

How much **income** did your household **as a whole** receive in the past twelve months from each of the following activities?

How much did your household spend in the past 12 months for each of the following expenditure items?

1. **Income (please ask for absolute values)**

| **Duration** | **Own Business/self-employment** | **Remittances** | **Pension** | **Other 1 (Specify)______** | **Other 2 (Specify)______** |
| --- | --- | --- | --- | --- | --- |
| Monthly |  |  |  |  |  |
| 12 months |  |  |  |  |  |

1. **Expenditure (Use the following scale). *First ask for absolute value. If not known, then scale***

| *Scale for monthly* | *Scale for 12 month (yearly)* |
| --- | --- |
| 1= < 100 5= 2001-3000  2= 100-500 6= 3001-4000  3= 501-1000 7= 4001-5000  4= 1001-2000 8= > 5000 | 1=<1,000 5=15,001-20,000  2=1,001-5,000 6=>20,000  3=5001-10,000  4=10,001-15,000 |

|  | Farming | Food | Education | Health | Housing | Clothing | Energy (cooking/lighting) | Savings | Supporting relatives | Communication | Ceremonies | Others  _________ |
| --- | --- | --- | --- | --- | --- | --- | --- | --- | --- | --- | --- | --- |
| Monthly |  |  |  |  |  |  |  |  |  |  |  |  |
| 12 months |  |  |  |  |  |  |  |  |  |  |  |  |

1. During the past 12 months, did your household borrow money to meet its needs if household income was not enough? *Circle* 1=Yes 2=No
2. **If Yes,** how much did you borrow for each for the following categories?  *First ask for absolute value. If not known, then scale as in Question 2*

| Duration | Farming food crop | Food | Education | Health | Housing | Clothing | Energy (cooking and lighting) | Farming feedstock | Supporting relatives | Communication | Ceremonies | Others  _________ |
| --- | --- | --- | --- | --- | --- | --- | --- | --- | --- | --- | --- | --- |
| Monthly |  |  |  |  |  |  |  |  |  |  |  |  |
| 12 months |  |  |  |  |  |  |  |  |  |  |  |  |

1. Please provide the number of household assets that you own

*Ask each asset category consecutively. Make it clear we are ONLY referring to members of the household that live in the household.*

*For each asset category insert the total number owned by all household members. Insert 0 if a specific asset category in not owned by any member.*

*For Other insert only important assets (i.e. not clothes, chairs, etc.)*

|  | Tick if you have | Indicate the number of those Ticked |
| --- | --- | --- |
| Watch/clock |  |  |
| Radio |  |  |
| Television |  |  |
| Mobile phone |  |  |
| Refrigerator |  |  |
| Improved stove |  |  |
| Sofa set |  |  |
| Chairs and Table |  |  |
| Truck |  |  |
| Bicycle |  |  |
| Motorcycle/scooter |  |  |
| Bed |  |  |
| Mattress |  |  |
| Car |  |  |
| Tractor |  |  |
| Ox-plough |  |  |
| Animal drawn-cart |  |  |
| Axe |  |  |
| Spade |  |  |
| Hoe |  |  |
| Boat (fishing) |  |  |
| Electricity generator |  |  |
| Pressure lamp |  |  |
| LED lantern |  |  |
| Solar panel |  |  |
| Treadle Pump |  |  |
| Sprayer |  |  |
| Other (specify)  __________________ |  |  |

**SECTION F: POVERTY INDICATORS**

*To be administered to all respondents*

**H1. Water supply and Sanitation**

1. What is the main source of water for members of your household? Insert code

| **Use** | **Source**  *1 = Piped water to dwelling 6= Spring*  *2= Piped water to plot/yard 7= Rainwater*  *3= Piped water to public tap/stand-pipe 8= River/lake/pond/dam*  *4= Borehole 9= Tanker truck*  *5= Dug well* |
| --- | --- |
| For drinking |  |
| For cooking |  |
| For washing/cleaning/showering |  |
| Others (specify______ |  |

1. How far is the water source for different household uses and how much do you pay? *Insert distance (in meters)* and time it takes to get to the source and back *(in minutes)*

| **Use** | 2.1 Distance (in meters) | 2.2 Time spent per day (in minutes) | 2.3 Cost per month (in Cedi) |
| --- | --- | --- | --- |
| For drinking |  |  |  |
| For cooking |  |  |  |
| For washing/cleaning/showering |  |  |  |
| Others (specify______ |  |  |  |

1. How would you describe the quality of water you use for cooking and drinking, now?

*1=Very good*

*2=Good*

*3=Bad*

*4=Very bad*

1. Do you (now) have enough water for cooking and drinking?

*1=Always enough*

*2=Sometimes enough*

*3=Usually not enough*

*4=Never enough*

1. What kind of toilet is available at your house? *Circle*.

*1=Pour flash toilet to pit latrine*

*2=Flush to piped sewer system*

*3=Open pit latrine*

*4=Pit latrine with slab*

*5=VIP latrine*

*6=Bucket toilet*

*7=Bush/field*

*8=Other (Specify)_____________*

1. Do you share your toilet facility with other households?

1=Yes

2=No

**H2. Housing**

What is the MAIN construction material of the MAIN dwelling of the household? *Observe and circle only one for each category.*

| 1. Floor | 1. Roof | 1. Exterior walls | 1. How old is your house? | 1. How many habitable room do you have? |
| --- | --- | --- | --- | --- |
| *1=Natural floor/ earth/sand*  *2=Dung*  *3=Palm/bamboo*  *4=Wood*  *5=Ceramic tiles*  *6=Cement*  *7=Carpet*  *8=Other (Specify)*  *______________* | *1=Leaf/ palm/ bamboo*  *2 = Mud*  *3=Wood planks*  *4=Card-board*  *5=Corrugated iron*  *6=Asbestos*  *7=Cement/concrete*  *8=Other (Specify)*  *___________________* | *1=Mud*  *2= Leaf/ palm/ bamboo*  *3=Card-board*  *4=Wood*  *5=Stone*  *6=Cement blocks*  *7=Mud bricks*  *8 = commercial bricks*  *9=Other (Specify)*  *___________________* | *1=<10 years*  *2=10-20*  *3=21-30*  *4=31-40*  *5=51-50* | *1=1-3*  *2=4-6*  *3=7-9*  *4=10-12*  *5=>12* |
|  |  |  | 1. Are you the owner of the house you stay in?   1=Yes  2=No | |

**H3: Health**

| 1. Has any member of your Household under 5 years died in the past 5 years? | 1. Are your household members covered by Health Insurance? | 1. How many members of your household are covered by health insurance? | 1. How long does it take to reach your nearest health facility?     _________ (in minutes) |
| --- | --- | --- | --- |
| 1=Yes  2=No  If yes, how many?_______ | 1=Yes  2=No | *1=All*  *2=1-3*  *3=7-9*  *4=4-6*  *5=10-12* |  |
| 1. Has any member of your household suffered from any sickness in the past 12 months?   1=Yes  2=No | | 1. What is the type of sickness?   1=Malaria 4= Tetanus 7=Diarrhoea  2=Measles 5= Whooping cough 8= Pneumonia  3=Meningitis 6= Tuberculosis 9=Other …………… | |

**G4: *Spill over effects of SMART OIL***

1. How much has your household benefited from these amenities developed by the company (SMART)? *Enter in the table below*

|  | *19.1*  *1=Yes,*  *2=No*  *3=Do not know* | 19.2  1=Very much  2=Much  3=Moderately  4=Little  5=Very little  6=Does not apply  7=Do not know |
| --- | --- | --- |
| Roads |  |  |
| Schools |  |  |
| Clinics |  |  |
| Dams/boreholes for water |  |  |
| Electricity |  |  |
| Other (Specify)_________ |  |  |

**SECTION G: ENERGY ACCESS AND USE**

*To be administered to all respondents*

1. Please indicate which of the following sources of energy is used for cooking and lighting?

|  | Firewood | Charcoal/  briquettes | Electricity | Ethanol | Kerosene/paraffin | Coal | Biogas | LPG | Agricultural residues | Dung | Other fuel 1 | Other fuel  _________ |
| --- | --- | --- | --- | --- | --- | --- | --- | --- | --- | --- | --- | --- |
| 1.1 Fuel used cooking  1=Yes 2=No |  |  |  |  |  |  |  |  |  |  |  |  |
| 1.2 Used for lighting  1=Yes 2=No |  |  |  |  |  |  |  |  |  |  |  |  |
| 1.3 Indicate the distance to access this source (meters) |  |  |  |  |  |  |  |  |  |  |  |  |

**SECTION H1: WOMEN EMPOWERMENT**

1. Now I’d like to ask you some questions about your participation in certain types of work activities and on making decisions on various aspects of household life

1.1 Did you yourself participate in [ACTIVITY] in the past 12 months (that is, during the last [one/two] cropping seasons), from August last year to August this year?

1.2 When decisions are made regarding [ACTIVITY], who is it that normally takes the decision? *CIRCLE ALL APPLICABLE. IF THE RESPONSE IS SELF ONLY SKIP*

1.3 How much input did you have in making decisions about [ACTIVITY]?

1.4 To what extent do you feel you can make your own personal decisions regarding [ACTIVITY] if you want(ed) to? *CIRCLE ONE*

- 1. How much input did you have in decisions on the use of income generated from [ACTIVITY]

|  | 1.1  Participation  1=Yes  2=No | 1.2 Who take decisions?  1=Respondent  2=Spouse  3=Other household member  4=NA | 1.3 H  1=No input in few decisions  2=Input in some decisions  3=Input in most or all decisions  4=No decision made | 1.4  1=Not at all  2=Small extent  3=Medium extent  4=High extent | 1.5  1=No input in few decisions  2=Input in some decisions  3=Input in most or all decisions  4=No decision made |
| --- | --- | --- | --- | --- | --- |
| 1. Food crop farming |  |  |  |  |  |
| 2. Cash crop farming |  |  |  |  |  |
| 3. Livestock raising |  |  |  |  |  |
| 4. Non-farm economic activity |  |  |  |  |  |
| 5. Wage/salary employment |  |  |  |  |  |
| 6. Other local activity  Specify__________ |  |  |  |  |  |
| 7. Major household expenditures |  |  |  |  |  |
| 8. Minor household expenditures |  |  |  |  |  |

2. Now I’d like to ask you about your household’s access to and ownership of a number of items that could be used to generate income

2.1 Does anyone in your household currently have any [ITEM]?

*2.2* Do you own any of the item? ***CIRCLE ALL APPLICABLE***

|  | 2.1  1=Yes  2=No | 2.2  1=Yes, solely  2=Yes, jointly  3=No |
| --- | --- | --- |
| 1. Agricultural land (pieces/plots) |  |  |
| 2. Large livestock (oxen, cattle) |  |  |
| 3. Small livestock (goats, pigs, sheep) |  |  |
| 4. Chickens, Ducks, Turkeys, Pigeons |  |  |
| 5. Fish pond or fishing equipment |  |  |
| 6. Farm equipment non-mechanized (e.g. hand tools, animal-drawn plough) |  |  |
| 7. Farm equipment mechanized (e.g. tractor-plough, power tiller, treadle pump) |  |  |
| 8. Non-farm business equipment  (e.g. solar panels used for recharging, sewing machine, brewing equipment, fryers) |  |  |
| 9. House or other structures |  |  |
| 10. Large consumer durables (tricycles, refrigerator, TV, sofa) |  |  |
| 11. Small consumer durables (radio, cookware) |  |  |
| 12. Cell phone |  |  |
| 13. Other land not used for agricultural purposes (e.g. pieces/plots, residential or commercial land) |  |  |
| 14. Means of transportation (e.g. bicycle, motorcycle, car) |  |  |

3. Next I’d like to ask about your household’s experience with borrowing money or other items in the past 12 months.”

3.1 Would you or anyone in your household be able to take a loan or borrow cash/in-kind from [SOURCE] if you wanted to? *

3.2 Has anyone in your household taken any loans or borrowed cash/in-kind from [SOURCE] in the past 12 months? ***CIRCLE ONE***

3.3 Who made the decision to borrow from [SOURCE] most of the time? ***CIRCLE ALL APPLICABLE***

3.4 Who makes the decision about what to do with the money/ item borrowed from [SOURCE] most of the time? **CIRCLE ALL APPLICABLE**

|  | 3.1  1=Yes  2=No  3=Maybe | 3.2  1=Yes, cash  2=Yes, in kind  3=Yes, cash and kind  4=No  5=Do not know | 3.3  1=Respondent  2=Spouse  3=Other household member  4= Other non-household member  5=NA | 3.4  1=Respondent  2=Spouse  3=Other household member  4= Other non-household member  5=NA |
| --- | --- | --- | --- | --- |
| 1. Non-governmental organization (NGO) |  |  |  |  |
| 2. Formal lender (bank/financial institution) |  |  |  |  |
| 3. Informal lender |  |  |  |  |
| 4. Friends or relatives |  |  |  |  |
| 5. Group based microfinance or lending including VSLAs |  |  |  |  |
| 6. Informal credit/savings groups such as merrygo-rounds, tontines, funeral societies, etc. |  |  |  |  |

4. Now I’m going to ask you about groups in the community. These can be either formal or informal and customary groups.

4.1 Is there a [GROUP] in your community?

4.2 Are you an active member of this [GROUP]?

|  | **4.1**  1=Yes  2=No  3=Do not know | **4.2**  1=Yes  2=No  3=Do not know |
| --- | --- | --- |
| 1. Agricultural / livestock/ fisheries producer’s group (including marketing groups) |  |  |
| 2. Water users’ group |  |  |
| 3. Forest users’ group |  |  |
| 4. Credit or microfinance group (including SACCOs/merry-go-rounds/ VSLAs) |  |  |
| 5. Mutual help or insurance group (including burial societies) |  |  |
| 6. Trade and business association group |  |  |
| 7. Civic groups (improving community) or charitable group (helping others) |  |  |
| 8. Religious group |  |  |
| 9. Other [women’s/men’s] group (only if it does not fit into one of the other categories) |  |  |
| 10. Other. Specify_________________________________________________ |  |  |

.5. Now I’d like to ask you about how you spent your time during the past 24 hours. We’ll begin from yesterday morning, and continue through to this morning. This will be a detailed accounting. I’m interested in everything you do (i.e. resting, eating, personal care, work inside and outside the home, caring for children, cooking, shopping, socializing, etc.), even if it doesn’t take you much time.


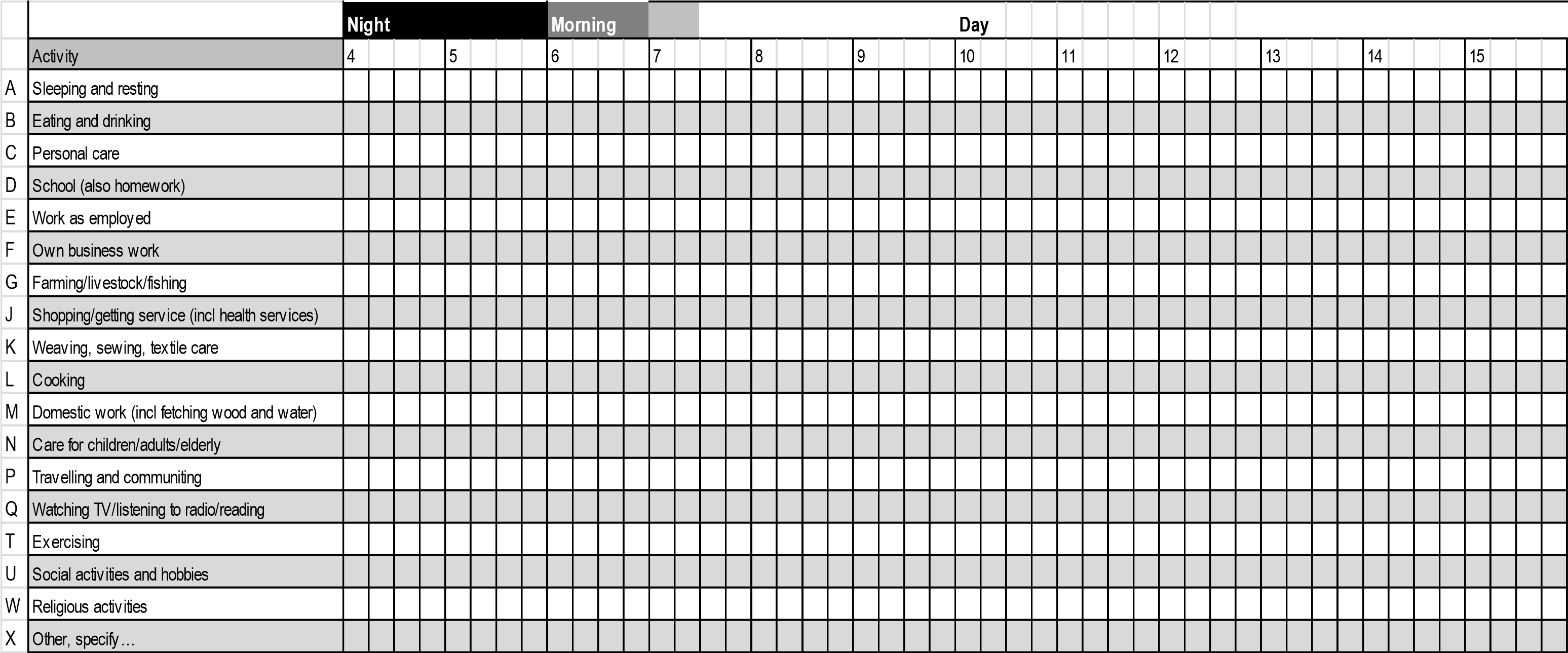


*
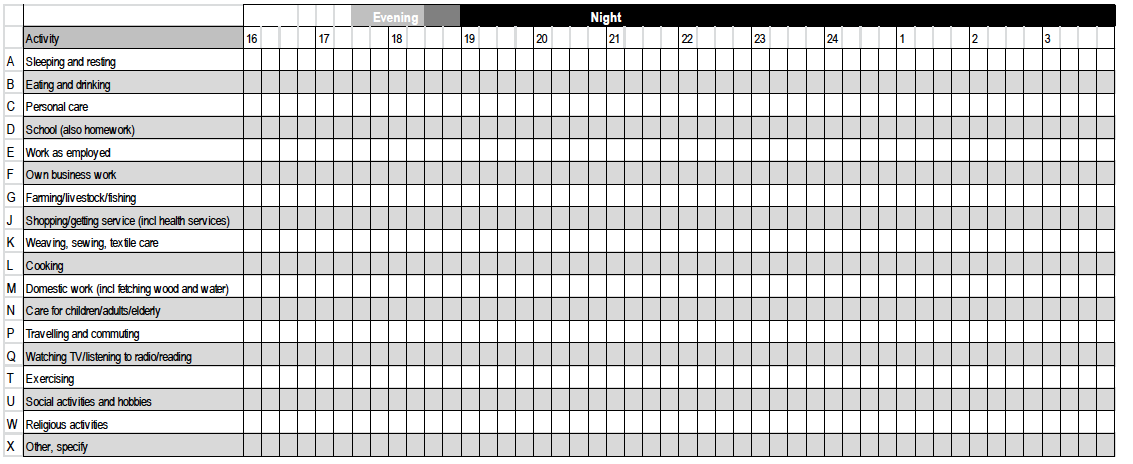
*

6. **In the last 24 hours did you work (at home or outside of the home) more than usual, about the same as usual, or less than usual?**

**1=More than usual**

**2=About the same as usual**

**3=Less than usual**

**SECTION H2: WOMEN EMPOWERMENT**

1. Now I’d like to ask you some questions about your participation in certain types of work activities and on making decisions on various aspects of household life

1.1 Did you yourself participate in [ACTIVITY] in the past 12 months (that is, during the last [one/two] cropping seasons), from August last year to August this year?

1.2 When decisions are made regarding [ACTIVITY], who is it that normally takes the decision? *CIRCLE ALL APPLICABLE. IF THE RESPONSE IS SELF ONLY SKIP*

1.3 How much input did you have in making decisions about [ACTIVITY]?

1.4 To what extent do you feel you can make your own personal decisions regarding [ACTIVITY] if you want(ed) to? *CIRCLE ONE*

- 1. How much input did you have in decisions on the use of income generated from [ACTIVITY]

|  | 1.1  Participation  1=Yes  2=No | 1.2 Who take decisions?  1=Respondent  2=Spouse  3=Other household member  4=NA | 1.3 H  1=No input in few decisions  2=Input in some decisions  3=Input in most or all decisions  4=No decision made | 1.4  1=Not at all  2=Small extent  3=Medium extent  4=High extent | 1.5  1=No input in few decisions  2=Input in some decisions  3=Input in most or all decisions  4=No decision made |
| --- | --- | --- | --- | --- | --- |
| 1. Food crop farming |  |  |  |  |  |
| 2. Cash crop farming |  |  |  |  |  |
| 3. Livestock raising |  |  |  |  |  |
| 4. Non-farm economic activity |  |  |  |  |  |
| 5. Wage/salary employment |  |  |  |  |  |
| 6. Other local activity  Specify__________ |  |  |  |  |  |
| 7. Major household expenditures |  |  |  |  |  |
| 8. Minor household expenditures |  |  |  |  |  |

2. Now I’d like to ask you about your household’s access to and ownership of a number of items that could be used to generate income

2.1 Does anyone in your household currently have any [ITEM]?

*2.2* Do you own any of the item? ***CIRCLE ALL APPLICABLE***

|  | 2.1  1=Yes  2=No | 2.2  1=Yes, solely  2=Yes, jointly  3=No |
| --- | --- | --- |
| 1. Agricultural land (pieces/plots) |  |  |
| 2. Large livestock (oxen, cattle) |  |  |
| 3. Small livestock (goats, pigs, sheep) |  |  |
| 4. Chickens, Ducks, Turkeys, Pigeons |  |  |
| 5. Fish pond or fishing equipment |  |  |
| 6. Farm equipment non-mechanized (e.g. hand tools, animal-drawn plough) |  |  |
| 7. Farm equipment mechanized (e.g. tractor-plough, power tiller, treadle pump) |  |  |
| 8. Non-farm business equipment  (e.g. solar panels used for recharging, sewing machine, brewing equipment, fryers) |  |  |
| 9. House or other structures |  |  |
| 10. Large consumer durables (tricycles, refrigerator, TV, sofa) |  |  |
| 11. Small consumer durables (radio, cookware) |  |  |
| 12. Cell phone |  |  |
| 13. Other land not used for agricultural purposes (e.g. pieces/plots, residential or commercial land) |  |  |
| 14. Means of transportation (e.g. bicycle, motorcycle, car) |  |  |

3. Next I’d like to ask about your household’s experience with borrowing money or other items in the past 12 months.”

3.1 Would you or anyone in your household be able to take a loan or borrow cash/in-kind from [SOURCE] if you wanted to? *

3.2 Has anyone in your household taken any loans or borrowed cash/in-kind from [SOURCE] in the past 12 months? ***CIRCLE ONE***

3.3 Who made the decision to borrow from [SOURCE] most of the time? ***CIRCLE ALL APPLICABLE***

3.4 Who makes the decision about what to do with the money/ item borrowed from [SOURCE] most of the time? **CIRCLE ALL APPLICABLE**

|  | 3.1  1=Yes  2=No  3=Maybe | 3.2  1=Yes, cash  2=Yes, in kind  3=Yes, cash and kind  4=No  5=Do not know | 3.3  1=Respondent  2=Spouse  3=Other household member  4= Other non-household member  5=NA | 3.4  1=Respondent  2=Spouse  3=Other household member  4= Other non-household member  5=NA |
| --- | --- | --- | --- | --- |
| 1. Non-governmental organization (NGO) |  |  |  |  |
| 2. Formal lender (bank/financial institution) |  |  |  |  |
| 3. Informal lender |  |  |  |  |
| 4. Friends or relatives |  |  |  |  |
| 5. Group based microfinance or lending including VSLAs |  |  |  |  |
| 6. Informal credit/savings groups such as merrygo-rounds, tontines, funeral societies, etc. |  |  |  |  |

4. Now I’m going to ask you about groups in the community. These can be either formal or informal and customary groups.

4.1 Is there a [GROUP] in your community?

4.2 Are you an active member of this [GROUP]?

|  | **4.1**  1=Yes  2=No  3=Do not know | **4.2**  1=Yes  2=No  3=Do not know |
| --- | --- | --- |
| 1. Agricultural / livestock/ fisheries producer’s group (including marketing groups) |  |  |
| 2. Water users’ group |  |  |
| 3. Forest users’ group |  |  |
| 4. Credit or microfinance group (including SACCOs/merry-go-rounds/ VSLAs) |  |  |
| 5. Mutual help or insurance group (including burial societies) |  |  |
| 6. Trade and business association group |  |  |
| 7. Civic groups (improving community) or charitable group (helping others) |  |  |
| 8. Religious group |  |  |
| 9. Other [women’s/men’s] group (only if it does not fit into one of the other categories) |  |  |
| 10. Other. Specify_________________________________________________ |  |  |

.5. Now I’d like to ask you about how you spent your time during the past 24 hours. We’ll begin from yesterday morning, and continue through to this morning. This will be a detailed accounting. I’m interested in everything you do (i.e. resting, eating, personal care, work inside and outside the home, caring for children, cooking, shopping, socializing, etc.), even if it doesn’t take you much time.


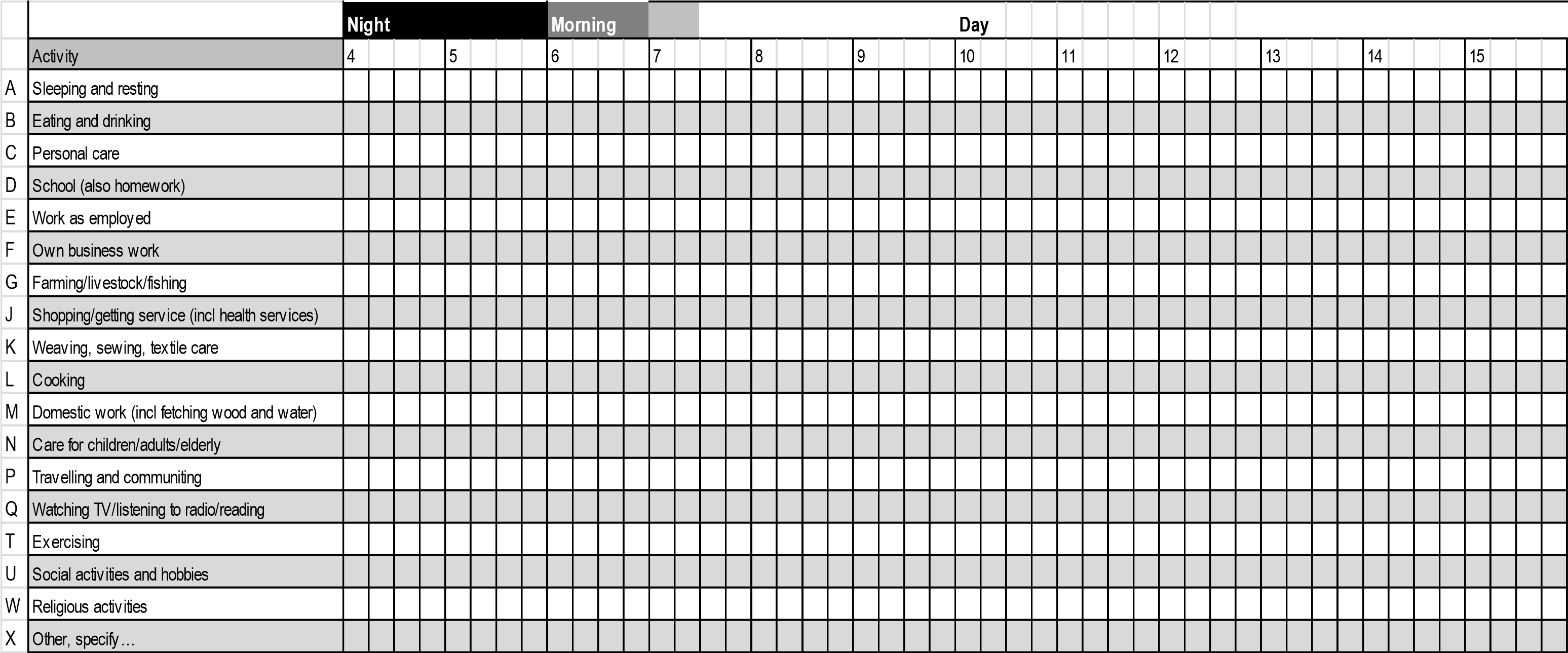


*
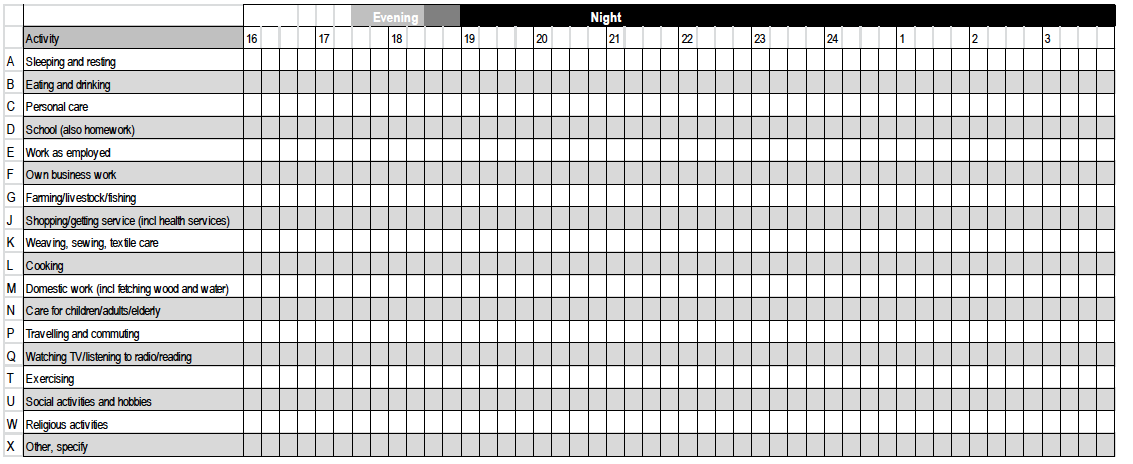
*

6. **In the last 24 hours did you work (at home or outside of the home) more than usual, about the same as usual, or less than usual?**

**1=More than usual**

**2=About the same as usual**

**3=Less than usual**

**SECTION I: RESILIENCE**

*To be administered to all respondents*

1. Over the past 12 months, from which of the following sources did your household receive income? *Tick*

2. Which of the following sources provided your household the most income? Which was the second source that provided the most income? Etc *Rank them starting with 1 for the source that provided the most income*

3. How stable was each income source? *Use the scale provided in the table.*

4. Which period of the year did the income received from these sources fluctuate? (Tick)

| Item | 1. Income  Source (Tick) | 2  Importance  (rank) | 3.  Stability  1=Highly stable  2= Moderately stable  3=Moderately unstable  4=Highly unstable | 4. Fluctuation (Tick) | | |
| --- | --- | --- | --- | --- | --- | --- |
|  |  |  |  | Dry | Wet | All year |
| Food crop production and sales |  |  |  |  |  |  |
| Company employee |  |  |  |  |  |  |
| Cultivation of other non-food crop |  |  |  |  |  |  |
| Sales of household assets (livestock) |  |  |  |  |  |  |
| Fishing |  |  |  |  |  |  |
| Sales of forest products |  |  |  |  |  |  |
| Formal employment |  |  |  |  |  |  |
| Own business or self-employment |  |  |  |  |  |  |
| Borrowing |  |  |  |  |  |  |
| Remittance |  |  |  |  |  |  |
| Other (Specify)___________________ |  |  |  |  |  |  |

1. Which of the following amenities do you have access to in your community? *Tick*
2. For which of these amenities access is only possible due to your work at Smart Oil? *Tick*
3. How stable is your access to each amenity? *Use the scale provided in the table.*
4. Explain how your involvement as a jatropha employee allow you to access the amenity and why it is stable or not

| **Category** | 5.  Access  (Tick) | 6.  Access to due to employment with Smart Oil  (Tick) | 7.  Stability  1=Highly stable  2=Moderately stable  3=Moderately unstable  4=Highly instable | 8. Explain why |
| --- | --- | --- | --- | --- |
| Community centre |  |  |  |  |
| Hospital |  |  |  |  |
| School |  |  |  |  |
| Weekly Market |  |  |  |  |
| Potable Water |  |  |  |  |
| Electricity |  |  |  |  |

1. Has your community experienced any of the following threats during the past 12 months?
2. Has any of the following threats affected your food crop cultivation in the past 12 months?
3. What was the most important threat to your farming activity in the past 12 months? *Rank the threats below in order of significance to farming activities. Start with 1 for the threat that had the largest impact*
4. When your household experienced these threats what did you do to cope?

| **Category** | **9. Experience of threats**  1=Yes  2-No | **10. Affecting farming**  1=Yes  2-No | **11. Rank the importance** | *12****. Coping strategies***  *1= Sell labour power*  *2= Short term migration*  *3= Borrow cash or grain*  *4= Sell livestock*  *5= Sell land*  *6=Use improved seed variety*  *7=Plant early or change planting times*  *8=Others specify___________* |
| --- | --- | --- | --- | --- |
| Drought |  |  |  |  |
| Flood |  |  |  |  |
| Soil erosion |  |  |  |  |
| Land degradation |  |  |  |  |
| Water pollution |  |  |  |  |
| Erratic rainfall |  |  |  |  |
| Pests |  |  |  |  |
| Other specify___ |  |  |  |  |

1. Which of the situations below has affected your household income (excluding income from jatropha) in the past 12 months? *Tick*
2. Which was the situation that has affected your non-jatropha household income the most in the past 12 months? *Rank in order of significance. Start with 1 for the situation that had the greatest effect on non-jatropha income*

|  | 13.  Effect | 14  Ranking |
| --- | --- | --- |
| Loss of formal employment |  |  |
| Collapse of personal business |  |  |
| Delays in payment of salaries/wages |  |  |
| Reduction in salaries/wages |  |  |
| Reduction in food crop selling prices |  |  |
| Limited Remittances |  |  |
| Loss of household labour |  |  |
| Loss of land |  |  |
| Drought |  |  |
| Floods |  |  |
| Erratic rainfalls |  |  |
| Increase in fertilizer/fuel price |  |  |
| Other (Specify)_________ |  |  |
| Other (Specify)_________ |  |  |

**For questions 15-17,19,22, and 25 Use the following scale**

**Ask the question in relation to the entire household and not only the respondent**

| 1 | Strongly Disagree |
| --- | --- |
| 2 | Disagree |
| 3 | Neither agree nor disagree |
| 4 | Agree |
| 5 | Strongly Agree |
| 6 | Not applicable |

1. Rate the following statements according to the scale above ***(for household income, excluding jatropha income). If clarification is needed such situations are included in question 13:***

| **Question** | **Rate** |
| --- | --- |
| If your household experiences this year a situation that can affect/reduce your income significantly, then you will be able to successfully cope with income reduction. |  |
| If this situation increases significantly (or combines with another situation that affects/reduces your income) in the next 5 years, your household would have the ability to adapt even if this requires to completely change your ways of life. |  |
| If this situation (or combination of situations) occurs this year, your household would have access to sufficient financial resources to ensure that you can fully recover |  |
| If this situation (or combination of situations) occurs this year, your household would be able to draw on support of family and friends to ensure that you fully recover |  |
| Your household has learned considerably from how you have dealt with past situations that has affected your income. |  |
| If situations that affect your income are expected this year, your household members would have access to early-warning information to ensure that you are fully prepared |  |

1. Rate the following statements according to the scale above *(for Flood)*

| **Question** | **Rate** |
| --- | --- |
| If flood occurs this year, your household would be able to successfully cope with the effects of the flood |  |
| If the rate of floods increases significantly in the next 5 years, your household would have the ability to adapt to the changing level of flooding even if this require to completely change your ways of life |  |
| If flood occurs this year, your household would have access to sufficient financial resources to ensure that you can fully recover from the effects of the flood |  |
| If flood occurs this year, your household would be able to draw on the support of family and friends to ensure that you fully recover from the effects of the flood |  |
| Your household has learned considerably from how you have dealt with flood events in the past |  |
| If flood occurs this year, your household members would have access to early-warning information to ensure that you are fully prepared for the flood |  |

1. Rate the following statements according to the scale above ***(for Drought)***

| **Question** | **Rate** |
| --- | --- |
| If drought occurs this year, your household would be able to successfully cope with the effects of the drought |  |
| If the rate of droughts increases significantly in the next 5 years, your household would have the ability to adapt to the changing level of drought even if this require to completely change your ways of life |  |
| If drought occurs this year, your household would have access to sufficient financial resources to ensure that you can fully recover from the effects of the drought |  |
| If drought occurs this year, your household would be able to draw on the support of family and friends to ensure that you fully recover from the effects of the drought |  |
| Your household has learned considerably from how you have dealt with drought events in the past |  |
| If drought occurs this year, your household members would have access to early-warning information to ensure that you are fully prepared for the drought |  |

**SECTION J: EMPLOYEES OF SMART OIL**

*To be administered to respondents who are EMPLOYED by SMART OIL company*

1. When did you start working for SMART OIL? Year
2. Why did you start working for SMART OIL?

*1=No access to land*

*2=No access to cash*

*3=Better than farming*

*4=Other (specify)* ___________

1. What is your position at the Company? ____________________

1. Did you have to move to start working for SMART OIL?

*1=Yes*

*2 =No*

1. If so, how many Km away from the plantation is your original home? Km
2. Does your household have access to land for farming? *Clarify that we do not refer to parents’ household, but the respondent’s own household. Circle*

*1=Yes*

*2 = No*

1. How far is it from the plantation? Km
2. Who farms MAINLY your land while you work for SMART OIL? *Circle*

*1 = Respondent in spare time (or out of season)*

*2 = Wife or husband*

*3 = Other household member*

*4 = Close friend/neighbor*

*5 = Hired labor*

*6= Other (please specify) _________*

1. How has your work for SMART OIL affected your farm yields? *Circle*

*1 = Yields Increased significantly*

*2 =Yields Increase moderately*

*3= Yields Remained Same*

*4 =Yields Decreased significantly*

*5=Yields Decreased moderately*

*6= Cannot tell*

1. Explain the answer___________________________________________________________________________________
2. Has your household lost land due to the expansion of SMART OIL?

1=Yes

2=No

1. If yes, what was the size of the land lost? ___________ha
2. What was this land used for? *Circle*.

*1=Unused*

*2=Subsistence farming (Specify main crops) ____________________*

*3=Livestock production/grazing*

*4=Cash crops (Specify main crops) ______________*

*5=Non-agricultural use (Specify uses) ________________*

*6=Commercially leased*

*7=Leased to relatives/friends at no cost*

*8=Other use (Specify) __________________*

1. Did you receive compensation for the land lost? *Circle*

1=Yes

2=No

***If, yes*:**

1. What was the form of this compensation? *Circle*

1=Equivalent amount of land

2=Smaller amount of land

3=Larger amount of land

4=Money

5=Other compensation, Specify______________

1. If you received money as compensation, can you specify sum? ________ Local currency
2. Was it enough to buy equivalent amount of land?

1=Yes

2=No

1. If you received money as compensation, what was the size? __________ ha
2. How did you get this land?

1=Allocated by chief

2=Allocated by government

3=Opened individually

4=Given by family

5=Buy

6=Other

1. Has working for the Company affected your household access to food? *Circle*

*1 = Much more access*

*2 = More Access*

*3=Same access*

*4= Less access*

*5=Much less access*

*6=Cannot tell*

1. What do you think is the MAIN reason for this effect on access to food? *Please ask as an open question and choose as appropriate. Circle ONLY one answer*

1=Loss of land

2=Loss of labour

3=High income

4=Stable income

5=Timing of the income

6=Access to fertilizer/pesticides

7=Loss of access to fertilizer/pesticides

8=Other (please specify) ______________________

1. Are you satisfied working for SMART OIL? *Circle*.

*1 = Not happy at all*

*2= Somewhat happy*

*3 = Moderately happy*

*4= Very happy*

1. Would you like to continue being engaged in the Company in the future? *Circle.*
2. *= Yes*
3. *= No*
4. *= Not sure*
5. Please provide reasons for your response above. *Summarize main reasons offered by respondent*

24.1 Main reason for continuation

_________________________________________________________________

24.2 Main reason for discontinuing

_______________________________________________________________________

**SECTION K. EFFECTS OF JATROPHA**

*To be administered to CONTROL GROUP WITHIN a jatropha growing area*

1. Why are you not currently farming jatropa? *Circle*

1=Not enough land

2=Not enough labour in household

3=Too far away from the mill/selling point

4=Too little profit to be made

5=My land does not meet the requirements for jatropha cultivation

6=Only want to grow food crops

7=My application to join SMART OIL was rejected

8= Other *(specify)* _______________________

1. Has your household lost land due to the expansion of SMART OIL?

1=Yes

2=No

1. If yes, what was the size of the land lost? ___________ha
2. What was this land used for? *Circle*.

*1=Unused*

*2=Subsistence farming (Specify main crops) ____________________*

*3=Livestock production/grazing*

*4=Cash crops (Specify main crops) ______________*

*5=Non-agricultural use (Specify uses) ________________*

*6=Commercially leased*

*7=Leased to relatives/friends at no cost*

*8=Other use (Specify) __________________*

1. Did you receive compensation for the land lost? *Circle*

1=Yes

2=No

***If, yes*:**

1. What was the form of this compensation? *Circle*

1=Equivalent amount of land

2=Smaller amount of land

3=Larger amount of land

4=Money

5=Other compensation, Specify______________

1. If you received money as compensation, can you specify sum? ________ Local currency
2. Was it enough to buy equivalent amount of land?

1=Yes

2=No

1. if you received money as compensation, what was the size? __________ ha
2. How did you get this land?

1=Allocated by chief

2=Allocated by government

3=Opened individually

4=Given by family

5=Buy

6=Other

1. Would you like to grow Jatropha in your land? *Circle*

1=Yes

2=No

3=Unsure

1. Please explain your response
2. If you were to grow Jatropha, would you do it independently or through the proposed out-grower scheme of Smart Oil?

1=Independently

2=Contracting scheme

3=Other. Specify_____________

4=Not sure

5=Do not know

1. Please explain your response
2. Would you be willing to allocate land to plant Jatropha?

1=Yes

2=No

1. If No, please explain (if Yes go to the next question)

**K. Wellbeing (to be asked to all groups)**

*To be asked to all groups*

| 1 Overall, how satisfied are you with your life?  *1= Not at all satisfied;*  *2= Somewhat satisfied*  *3 = Moderately satisfied*  *4=Very satisfied* | 1. Overall, to what extent do you feel the things you do in your life are worthwhile?   *1 = Not at all*  *2= Somewhat worthwhile*  *3= Moderately worthwhile*  *4=Very worthwhile* |
| --- | --- |
| 1. Overall, how happy do you feel?   *1 = Not at all;*  *2 = Somewhat happy;*  *3 = Moderately happy;*  *4=Very happy* | 1. Overall, how anxious do you feel?   *1= Not at all;*  *2= Somewhat anxious;*  *3=Moderately anxious;*  *4 =Very anxious* |

**M. General comments**

*To be asked to all groups*

Do you have any general comments on this study/interview?

_____________________________________________________________________________________________

_____________________________________________________________________________________________

*Thank the respondents for their time*

General comments from interviewer

__________________________________________________________________________________________________________________________________________________________________________________________
