# Supplementary material for: Human wellbeing outcomes of involvement in industrial crop production: Evidence from sugarcane, oil palm and jatropha sites in Ghana
Source: PLoS One. 2019 Apr 25;14(4):e0215433. doi: 10.1371/journal.pone.0215433 (PMC6483194; doi:10.1371/journal.pone.0215433)
Supplement: S2 File — (DOCX) [file pone.0215433.s002.docx]

Table A: Confounders used for the propensity score estimations

| **Variable** | **Variable in analysis** | **Measurement** |
| --- | --- | --- |
| Age of respondents | Age | Years |
| Education of respondents | Education | Dummy, Education=1, No education=0 |
| Household size | Adult_Equivalent | Number |
| Percentage of off-farm income | percentageofffarmincome | Ratio of Off-farm income to Total household income |
| Farm size | Cultivatedland | Hectares |

Table B: Probit regression estimation for participation in jatropha production [Jatropha Permanent Workers (JPW) and Control (JC) in Yeji]

-----------------------------------------------------------------------------------------

InvolvedinIC | Coef. Std. Err. z P>|z| [95% Conf. Interval]

------------------------+----------------------------------------------------------------

Age | -.0221958 .0088986 -2.49 0.013 -.0396368 -.0047549

Education | .0913117 .2343133 0.39 0.697 -.3679339 .5505574

Adult_Equivalent | -.053853 .068162 -0.79 0.429 -.1874479 .079742

percentageofffarmincome | -4.15139 1.01776 -4.08 0.000 -6.146162 -2.156618

Cultivatedland | -.1781678 .0395752 -4.50 0.000 -.2557338 -.1006017

_cons | 2.253967 .5006299 4.50 0.000 1.27275 3.235184

Table C: Probit regression estimation for participation in sugarcane production [Sugarcane Smallholders (DB_S) and Control (DB_NS) in Dabala]

-----------------------------------------------------------------------------------------

InvolvedinIC | Coef. Std. Err. z P>|z| [95% Conf. Interval]

------------------------+----------------------------------------------------------------

Age | -.0325998 .0106736 -3.05 0.002 -.0535198 -.0116799

Education | .3655482 .2081832 1.76 0.079 -.0424835 .7735798

Adult_Equivalent | .0536515 .0994564 0.54 0.590 -.1412795 .2485824

percentageofffarmincome | -1.493333 .3830827 -3.90 0.000 -2.244162 -.7425051

Cultivatedland | .0391931 .0266532 1.47 0.141 -.0130462 .0914325

_cons | 1.367954 .5596544 2.44 0.015 .2710513 2.464856

-----------------------------------------------------------------------------------------

Table D: Probit regression estimation for participation in oil palm production [Oil palm workers (KW_W) and Control (KW_C) in Kwae]

-----------------------------------------------------------------------------------------

InvolvedinIC | Coef. Std. Err. z P>|z| [95% Conf. Interval]

------------------------+----------------------------------------------------------------

Age | -.0668251 .013372 -5.00 0.000 -.0930336 -.0406165

Education | -.3672209 .271364 -1.35 0.176 -.8990846 .1646428

Adult_Equivalent | -.3447523 .1456662 -2.37 0.018 -.6302529 -.0592517

percentageofffarmincome | -.7599128 .5233943 -1.45 0.147 -1.785747 .2659212

Cultivatedland | -.2249291 .0426197 -5.28 0.000 -.3084621 -.1413961

_cons | 4.618062 .6521015 7.08 0.000 3.339966 5.896157

-----------------------------------------------------------------------------------------

Table E: Probit regression estimation for participation in oil palm production [Oil palm outgrowers (KW_OG) and Control (KW_C) in Kwae]

-----------------------------------------------------------------------------------------

InvolvedinIC | Coef. Std. Err. z P>|z| [95% Conf. Interval]

------------------------+----------------------------------------------------------------

Age | -.0122779 .0172784 -0.71 0.477 -.0461431 .0215872

Education | .2320356 .3343267 0.69 0.488 -.4232326 .8873039

Adult_Equivalent | -.0353701 .1626369 -0.22 0.828 -.3541325 .2833923

percentageofffarmincome | -1.505515 .7951916 -1.89 0.058 -3.064062 .0530314

Cultivatedland | .2376258 .0865343 2.75 0.006 .0680217 .4072299

_cons | -1.440066 1.060441 -1.36 0.174 -3.518493 .6383603

-----------------------------------------------------------------------------------------

Table F: Probit regression estimation for participation in oil palm production [Oil palm independent smallholders (KW_ID) and Control (KW_C) in Kwae]

-------------------------------------------------------------------------------------------

InvolvedinIC | Coef. Std. Err. z P>|z| [95% Conf. Interval]

--------------------------+----------------------------------------------------------------

Age | .0074478 .0126318 0.59 0.555 -.0173101 .0322057

Education | .3568767 .2888362 1.24 0.217 -.2092319 .9229853

AdultEquivalent | .1927573 .1249001 1.54 0.123 -.0520423 .437557

percentageofofffarmincome | -1.558429 .6626492 -2.35 0.019 -2.857197 -.2596601

Cultivated_land | .2069613 .0717182 2.89 0.004 .0663962 .3475264

_cons | -2.64446 .9607915 -2.75 0.006 -4.527577 -.7613435

Table G: Balancing test with different algorithms for Jatropha Permanent Workers (YJ_JPW) and Control (YJ_JC)

|  | No. Of Significant. Variables | Pseudo R2 | p-Value LR* Test | Mean Bias |
| --- | --- | --- | --- | --- |
| Impact of involvement in IC on |  |  |  |  |
| **Household Income** |  |  |  |  |
| **Before matching** | 3 | 0.205 | 0.000 | 42.1 |
| Radius Caliper (0.1) | 0 | 0.003 | 0.976 | 4.7 |
| Nearness neighbour | 0 | 0.007 | 0.862 | 7.2 |
| Kernel common trim (0.1) | 0 | 0.006 | 0.89 | 7.0 |
| **Per capita income** |  |  |  |  |
| **Before matching** | 3 | 0.205 | 0.000 | 42.1 |
| Radius Caliper (0.1) | 0 | 0.003 | 0.976 | 4.7 |
| Nearness neighbour | 0 | 0.007 | 0.862 | 7.2 |
| Kernel common trim | 0 | 0.006 | 0.89 | 7.0 |
| **Household consumption** |  |  |  |  |
| **Before matching** | 3 | 0.205 | 0.000 | 42.1 |
| Radius Caliper (0.1) | 0 | 0.003 | 0.976 | 4.7 |
| Nearness neighbour | 0 | 0.007 | 0.862 | 7.2 |
| Kernel common trim | 0 | 0.006 | 0.89 | 7.0 |
| **Per capita consumption** |  |  |  |  |
| Before matching | 3 | 0.205 | 0.000 | 42.1 |
| Radius Caliper (0.1) | 0 | 0.003 | 0.976 | 4.7 |
| Nearness neighbour | 0 | 0.007 | 0.862 | 7.2 |
| Kernel common trim (0.1) | 0 | 0.006 | 0.89 | 7.0 |

Figure A: Histogram of propensity scores for jatropha permanent workers (YJ_JPW) and control group (YJ_JC)

Table H: Balancing test with different algorithms for Sugarcane Smallholders (DB_S) and Control (DB_NS)

|  | No. Of Significant. Variables | Pseudo R2 | p-Value LR* Test | Mean Bias |
| --- | --- | --- | --- | --- |
| Impact of involvement in IC on |  |  |  |  |
| **Household Income** |  |  |  |  |
| **Before matching** | 2 | 0.102 | 0.00 | 23.0 |
| Radius Caliper (0.1) | 0 | 0.002 | 0.992 | 2.6 |
| Nearness neighbour | 0 | 0.017 | 0.533 | 8.7 |
| Kernel common trim (0.1) | 0 | 0.001 | 0.998 | 3.2 |
|  |  |  |  |  |
| **Per capita income** |  |  |  |  |
| **Before matching** | 2 | 0.102 | 0.00 | 23.0 |
| Radius Caliper (0.1) | 0 | 0.002 | 0.992 | 2.6 |
| Nearness neighbour | 0 | 0.017 | 0.533 | 8.7 |
| Kernel common trim | 0 | 0.001 | 0.998 | 3.2 |
|  |  |  |  |  |
| **Household consumption** |  |  |  |  |
| **Before matching** | 2 | 0.102 | 0.00 | 23.0 |
| Radius Caliper (0.1) | 0 | 0.002 | 0.992 | 2.6 |
| Nearness neighbour | 0 | 0.017 | 0.533 | 8.7 |
| Kernel common trim | 0 | 0.001 | 0.998 | 3.2 |
|  |  |  |  |  |
| **Per capita consumption** |  |  |  |  |
| Before matching | 2 | 0.102 | 0.00 | 23.0 |
| Radius Caliper (0.1) | 0 | 0.002 | 0.992 | 2.6 |
| Nearness neighbour | 0 | 0.017 | 0.533 | 8.7 |
| Kernel common trim (0.1) | 0 | 0.001 | 0.998 | 3.2 |

Figure B: Histogram of propensity scores for sugarcane smallholders (DB_S) and control group (DB_NS)

Table I: Balancing test with different algorithms for Oil palm workers (KW_GW) and Control (KW_C)

|  | No. Of Significant. Variables | Pseudo R2 | p-Value LR* Test | Mean Bias |
| --- | --- | --- | --- | --- |
| Impact of involvement in IC on |  |  |  |  |
| **Household Income** |  |  |  |  |
| **Before matching** | 3 | 0.172 | 0.000 | 42.9 |
| Radius Caliper (0.1) | 0 | 0.028 | 0.815 | 15.0 |
| Nearness neighbour | 0 | 0.041 | 0.650 | 21.4 |
| Kernel common trim (0.1) | 0 | 0.031 | 0.775 | 15.4 |
| **Per capita income** |  |  |  |  |
| **Before matching** | 3 | 0.172 | 0.000 | 42.9 |
| Radius Caliper (0.1) | 0 | 0.028 | 0.815 | 15.0 |
| Nearness neighbour | 0 | 0.041 | 0.650 | 21.4 |
| Kernel common trim | 0 | 0.031 | 0.775 | 15.4 |
| **Household consumption** |  |  |  |  |
| **Before matching** | 3 | 0.172 | 0.000 | 42.9 |
| Radius Caliper (0.1) | 0 | 0.028 | 0.815 | 15.0 |
| Nearness neighbour | 0 | 0.041 | 0.650 | 21.4 |
| Kernel common trim | 0 | 0.031 | 0.775 | 15.4 |
| **Per capita consumption** |  |  |  |  |
| Before matching | 3 | 0.172 | 0.000 | 42.9 |
| Radius Caliper (0.1) | 0 | 0.028 | 0.815 | 15.0 |
| Nearness neighbour | 0 | 0.041 | 0.650 | 21.4 |
| Kernel common trim (0.1) | 0 | 0.031 | 0.775 | 15.4 |

Figure C: Histogram of propensity scores for oil palm workers (KW_GW) and control group (KW_C)

Table J: Balancing test with different algorithms for Oil palm outgrowers (KW_OG) and Control (KW_C)

|  | No. Of Significant. Variables | Pseudo R2 | p-Value LR* Test | Mean Bias |
| --- | --- | --- | --- | --- |
| Impact of involvement in IC on |  |  |  |  |
| **Household Income** |  |  |  |  |
| **Before matching** | 2 | 0.133 | 0.033 | 31.2 |
| Radius Caliper (0.1) | 0 | 0.037 | 0.858 | 13.6 |
| Nearness neighbour | 0 | 0.119 | 0.282 | 25.3 |
| Kernel common trim (0.1) | 0 | 0.047 | 0.777 | 15.7 |
| **Per capita income** |  |  |  |  |
| **Before matching** | 2 | 0.133 | 0.033 | 31.2 |
| Radius Caliper (0.1) | 0 | 0.037 | 0.858 | 13.6 |
| Nearness neighbour | 0 | 0.119 | 0.282 | 25.3 |
| Kernel common trim | 0 | 0.047 | 0.777 | 15.7 |
| **Household consumption** |  |  |  |  |
| **Before matching** | 2 | 0.133 | 0.033 | 31.2 |
| Radius Caliper (0.1) | 0 | 0.037 | 0.858 | 13.6 |
| Nearness neighbour | 0 | 0.119 | 0.282 | 25.3 |
| Kernel common trim | 0 | 0.047 | 0.777 | 15.7 |
| **Per capita consumption** |  |  |  |  |
| Before matching | 2 | 0.133 | 0.033 | 31.2 |
| Radius Caliper (0.1) | 0 | 0.037 | 0.858 | 13.6 |
| Nearness neighbour | 0 | 0.119 | 0.282 | 25.3 |
| Kernel common trim (0.1) | 0 | 0.047 | 0.777 | 15.7 |

Figure D: Histogram of propensity scores for oil palm outgrowers (KW_OG) and control group (KW_C)

Table K: Balancing test with different algorithms for Oil palm independent smallholders (KW_ID) and control (KW_C)

|  | No. Of Significant. Variables | Pseudo R2 | p-Value LR* Test | Mean Bias |
| --- | --- | --- | --- | --- |
| Impact of involvement in IC on |  |  |  |  |
| **Household Income** |  |  |  |  |
| **Before matching** | 2 | 0.135 | 0.005 | 27.7 |
| Radius Caliper (0.1) | 0 | 0.008 | 0.990 | 8.2 |
| Nearness neighbour | 0 | 0.015 | 0.951 | 8.5 |
| Kernel common trim (0.1) | 0 | 0.007 | 0.992 | 6.8 |
|  |  |  |  |  |
| **Per capita income** |  |  |  |  |
| **Before matching** | 2 | 0.135 | 0.005 | 27.7 |
| Radius Caliper (0.1) | 0 | 0.008 | 0.990 | 8.2 |
| Nearness neighbour | 0 | 0.015 | 0.951 | 8.5 |
| Kernel common trim | 0 | 0.007 | 0.992 | 6.8 |
|  |  |  |  |  |
| **Household consumption** |  |  |  |  |
| **Before matching** | 2 | 0.135 | 0.005 | 27.7 |
| Radius Caliper (0.1) | 0 | 0.008 | 0.990 | 8.2 |
| Nearness neighbour | 0 | 0.015 | 0.951 | 8.5 |
| Kernel common trim | 0 | 0.007 | 0.992 | 6.8 |
|  |  |  |  |  |
| **Per capita consumption** |  |  |  |  |
| Before matching | 2 | 0.135 | 0.005 | 27.7 |
| Radius Caliper (0.1) | 0 | 0.008 | 0.990 | 8.2 |
| Nearness neighbour | 0 | 0.015 | 0.951 | 8.5 |
| Kernel common trim (0.1) | 0 | 0.007 | 0.992 | 6.8 |

Figure E: Histogram of propensity scores for oil palm independent smallholders (KW_ID) and control group (KW_C)

Table L: Sensitivity analysis- Rosenbaum bounds results for participation in jatropha production [Jatropha Permanent Workers (JPW) and Control (JC) in Yeji]

**Total Household income**

Gamma sig+ sig- t-hat+ t-hat- CI+ CI-

----------------------------------------------------------------------

1 .01514 .01514 377.135 377.135 92.3368 671.846

1.1 .038571 .005082 314.14 449.746 26.0104 749.437

1.2 .079729 .00162 241.798 515.865 -35.4243 826.01

1.3 .140622 .000496 184.823 579.823 -102.566 885.314

1.4 .219428 .000147 138.542 628.641 -149.949 947.688

1.5 .311136 .000043 86.9013 678.531 -196.668 1005.87

1.6 .409105 .000012 41.8737 730.298 -239.15 1061.99

1.7 .506692 3.4e-06 -2.18419 783.406 -291.667 1124.63

1.8 .598439 9.2e-07 -35.5386 826.225 -329.035 1175.17

1.9 .680626 2.5e-07 -74.6862 862.612 -362.219 1213.85

2 .751308 6.7e-08 -116.067 904.094 -409.302 1257.14

**Per capita income**

Gamma sig+ sig- t-hat+ t-hat- CI+ CI-

----------------------------------------------------------------------

1 .502257 .502257 -.925781 -.925781 -93.6948 101.148

1.1 .657998 .346171 -27.024 22.8589 -114.74 128.945

1.2 .780591 .222782 -46.5823 41.9126 -134.45 152.849

1.3 .867199 .135266 -63.3473 62.9488 -149.445 177.208

1.4 .923461 .078202 -80.229 81.8052 -165.6 205.439

1.5 .957666 .043387 -95.1702 104.394 -179.965 228.616

1.6 .977383 .023249 -107.358 121.4 -194.924 248.862

1.7 .988266 .012098 -120.827 138.367 -207.89 272.679

1.8 .994063 .006139 -134.638 154.361 -218.388 293.941

1.9 .997059 .003049 -143.734 167.241 -228.782 314.367

2 .99857 .001487 -153.149 183.46 -239.285 330.125

**Household Consumption**

Gamma sig+ sig- t-hat+ t-hat- CI+ CI-

----------------------------------------------------------------------

1 9.3e-06 9.3e-06 -590.383 -590.383 -809.458 -379.981

1.1 1.4e-06 .000051 -642 -540.55 -863.424 -329.236

1.2 2.0e-07 .000207 -695.33 -495.695 -907.049 -278.583

1.3 2.9e-08 .000661 -738.533 -454.791 -949.133 -229.083

1.4 4.1e-09 .001752 -772.125 -407.667 -988.813 -191.844

1.5 5.7e-10 .004004 -814.163 -373.189 -1026.13 -151.958

1.6 7.9e-11 .008108 -851.533 -340.245 -1061.97 -120.083

1.7 1.1e-11 .014867 -878.555 -307.927 -1095.54 -85.5697

1.8 1.5e-12 .025103 -907.401 -277.708 -1125.1 -46.65

1.9 2.0e-13 .039551 -935.286 -245.975 -1151.71 -16.1333

2 2.8e-14 .058767 -959.313 -221.708 -1183.82 13.125

2.1 3.8e-15 .083063 -984.208 -196.824 -1213.05 37.0555

2.2 5.6e-16 .112475 -1007.38 -175.048 -1241.49 66.875

2.3 1.1e-16 .146764 -1029.45 -149.991 -1266.89 97.0589

2.4 0 .185451 -1049.85 -132.494 -1297.99 123.969

2.5 0 .227866 -1074.57 -111.538 -1317.36 149.75

2.6 0 .27321 -1091.41 -89.6979 -1341.43 173.988

2.7 0 .320614 -1109.97 -71 -1361.81 205.25

2.8 0 .369198 -1125.25 -45.9614 -1383.53 221.774

2.9 0 .418118 -1142.38 -28.2961 -1404.16 248.181

3 0 .4666 -1157.64 -10.8437 -1430.08 273.083

**Per capita consumption**

Gamma sig+ sig- t-hat+ t-hat- CI+ CI-

----------------------------------------------------------------------

1 .000023 .000023 -196.47 -196.47 -266.919 -128.057

1.1 3.7e-06 .000118 -212.278 -179.063 -287.152 -106.913

1.2 5.9e-07 .000445 -226.38 -163.931 -305.66 -89.8744

1.3 9.1e-08 .001341 -240.818 -152.225 -321.077 -71.4849

1.4 1.4e-08 .003371 -255.05 -138.657 -332.796 -56.7706

1.5 2.1e-09 .007342 -268.14 -126.518 -343.594 -40.7168

1.6 3.1e-10 .01423 -282.952 -111.868 -357.185 -27.7542

1.7 4.6e-11 .025064 -293.891 -99.6499 -368.177 -17.6546

1.8 6.8e-12 .040773 -305.843 -89.5523 -377.454 -4.3965

1.9 1.0e-12 .062055 -314.857 -79.6115 -384.734 6.52144

2 1.5e-13 .089276 -323.89 -67.9801 -394.266 17.0669

2.1 2.1e-14 .122431 -331.254 -58.1455 -401.623 27.61

2.2 3.1e-15 .16115 -337.922 -49.0888 -410.565 36.3163

2.3 4.4e-16 .204752 -345.173 -40.0644 -419.427 47.942

2.4 1.1e-16 .252324 -352.965 -31.9167 -430.153 56.1284

2.5 0 .302808 -360.132 -23.743 -439.913 65.929

2.6 0 .355092 -366.397 -18.6258 -447.881 73.6886

2.7 0 .408085 -371.635 -11.8085 -454.995 81.8445

2.8 0 .460775 -377.68 -4.2776 -461.651 91.3658

2.9 0 .512277 -382.305 3.27081 -469.391 98.212

3 0 .561852 -386.823 8.50448 -474.062 105.935

Table M: Sensitivity analysis - Rosenbaum bounds results for participation in sugarcane production [Sugarcane Smallholders (DB_S) and Control (DB_NS) in Dabala]

**Total Household Income**

Gamma sig+ sig- t-hat+ t-hat- CI+ CI-

----------------------------------------------------------------------

1 4.3e-08 4.3e-08 2821.21 2821.21 1917.67 3793.49

1.1 3.3e-07 4.5e-09 2625.89 3034.94 1717.48 4029.05

1.2 1.8e-06 4.6e-10 2433.53 3241.33 1523.1 4231.51

1.3 7.5e-06 4.8e-11 2255.46 3407.08 1366.36 4455.85

1.4 .000025 4.9e-12 2066.21 3595.25 1215.95 4671.63

1.5 .000072 5.1e-13 1924.17 3779.54 1097.65 4867.32

1.6 .000178 5.2e-14 1791.41 3925.04 981.659 5067.79

1.7 .000393 5.3e-15 1668.28 4073.02 880.387 5244.13

1.8 .000792 5.6e-16 1556.04 4203.29 782.362 5421.43

1.9 .001472 0 1446.44 4335.66 701.471 5552.68

2 .002559 0 1345.08 4470.2 601.167 5707.44

2.1 .004198 0 1264.31 4597.79 509.475 5840.41

2.2 .006551 0 1189.71 4720.32 432.549 5991.19

2.3 .009788 0 1111.35 4831.05 374.532 6137.94

2.4 .014077 0 1045.45 4959.54 305.379 6241.79

2.5 .019581 0 977.341 5071.8 219.865 6423.85

2.6 .026443 0 905.403 5176.36 157.29 6568.39

2.7 .034784 0 860.153 5279.29 79.3125 6692.13

2.8 .044698 0 802.631 5368.53 18.994 6836.53

2.9 .056248 0 754.047 5473.44 -32.1515 6975.74

3 .069463 0 708.847 5539.44 -69.9582 7085.13

3.1 .084341 0 647.384 5643.49 -112.155 7193.85

3.2 .100848 0 601.167 5707.44 -159.742 7300.98

3.3 .118921 0 541.41 5775.98 -206.902 7382.75

3.4 .138474 0 499.645 5850.23 -266.121 7438.28

3.5 .159397 0 462.468 5946.59 -292.306 7547.51

**Per capita income**

Gamma sig+ sig- t-hat+ t-hat- CI+ CI-

----------------------------------------------------------------------

1 5.2e-06 5.2e-06 510.885 510.885 305.986 766.391

1.1 .000028 7.8e-07 460.694 563.092 258.55 829.38

1.2 .000114 1.2e-07 409.156 616.72 224.545 887.839

1.3 .000367 1.7e-08 369.574 674.639 186.523 939.238

1.4 .000981 2.5e-09 336.76 729.343 152.802 992.57

1.5 .002267 3.5e-10 309.588 763.448 128.21 1041.52

1.6 .004653 5.1e-11 279.136 808.89 100.917 1085.28

1.7 .008663 7.2e-12 250.088 842.922 77.0633 1126.27

1.8 .014875 1.0e-12 228.87 876.589 59.0978 1162.69

1.9 .023857 1.4e-13 207.292 908.718 37.3519 1193.68

2 .036115 2.0e-14 185.141 942.294 17.2575 1230.29

2.1 .052036 2.9e-15 162.039 976.379 -2.77847 1256.4

2.2 .071858 4.4e-16 145.732 1001.05 -19.4649 1299.17

2.3 .095645 1.1e-16 130.854 1033.39 -36.7401 1327.96

2.4 .123297 0 117.363 1065.92 -51.8065 1353.09

2.5 .154554 0 100.123 1087.9 -66.2561 1380.12

2.6 .189028 0 85.784 1108.21 -78.405 1413.11

2.7 .226233 0 73.3072 1134.22 -92.4474 1436.76

2.8 .265617 0 62.339 1152.88 -105.807 1461.36

2.9 .306595 0 50.2939 1174.19 -119.295 1481.84

3 .348576 0 37.8433 1188.68 -129.285 1501.93

3.1 .390993 0 28.9461 1210.05 -145.038 1522.47

3.2 .433317 0 17.2575 1230.29 -153.372 1544.34

3.3 .47507 0 5.84775 1246.3 -164.72 1561.1

3.4 .51584 0 -4.37975 1256.93 -178.733 1580.28

3.5 .555277 0 -11.6194 1278.44 -188.081 1600

**Household Consumption**

Gamma sig+ sig- t-hat+ t-hat- CI+ CI-

----------------------------------------------------------------------

1 .003595 .003595 305.607 305.607 116.001 482.345

1.1 .010651 .001037 258.305 341.286 74.6387 529.085

1.2 .025269 .000288 223.163 380.224 38.7697 563.626

1.3 .050578 .000078 184.464 415.3 -1.96572 593.918

1.4 .088676 .00002 148.593 450.239 -43.8259 632.61

1.5 .139993 5.3e-06 118.172 479.976 -75.3584 660.493

1.6 .203197 1.3e-06 91.4328 507.861 -109.311 700.889

1.7 .275547 3.3e-07 66.2967 536.174 -139.115 724.578

1.8 .353503 8.3e-08 41.7241 558.781 -157.435 748.621

1.9 .433357 2.0e-08 18.2315 579.091 -181.674 774.074

2 .511736 5.0e-09 -4.67618 596.171 -201.989 801.687

**Per capita consumption**

Gamma sig+ sig- t-hat+ t-hat- CI+ CI-

----------------------------------------------------------------------

1 .00135 .00135 51.8213 51.8213 23.5879 81.0495

1.1 .004461 .000347 44.3615 58.1712 17.1105 89.8492

1.2 .011642 .000086 38.4689 64.6942 12.6407 96.8435

1.3 .025349 .000021 32.4791 70.9663 6.45037 106.692

1.4 .047912 4.9e-06 28.3934 75.6128 .640255 114.374

1.5 .080929 1.2e-06 23.7165 80.9595 -3.76179 120.94

1.6 .124871 2.6e-07 19.7518 85.8846 -7.98734 127.563

1.7 .178986 6.0e-08 16.243 91.0915 -11.2837 134.338

1.8 .24149 1.4e-08 13.0457 95.3163 -14.018 141.934

1.9 .309909 3.0e-09 9.98277 100.77 -17.9198 148.715

2 .381489 6.7e-10 5.9437 106.952 -21.4488 158.022

Table N: Sensitivity analysis - Rosenbaum bounds results for participation in oil palm production [Oil palm workers (KW_W) and Control (KW_C) in Kwae]

**Total Household income**

Gamma sig+ sig- t-hat+ t-hat- CI+ CI-

----------------------------------------------------------------------

1 .023932 .023932 -1044.87 -1044.87 -1913.03 -201.5

1.1 .013726 .039492 -1125.55 -933.721 -2024.34 -49.3

1.2 .007843 .05968 -1252.32 -799.3 -2202.62 70.8786

1.3 .00447 .084274 -1413.48 -718.732 -2301.5 243.169

1.4 .002542 .112821 -1523.19 -671.02 -2420.23 417.679

1.5 .001444 .144721 -1554.29 -632.007 -2553 518.129

1.6 .000819 .179307 -1619.57 -566.787 -2686.37 571.741

1.7 .000465 .2159 -1677.25 -540.541 -2737.59 665.812

1.8 .000263 .253849 -1721.84 -487.783 -2816.85 836.45

1.9 .000149 .292558 -1767.57 -361.025 -2883.28 936.9

2 .000084 .3315 -1855.87 -251.647 -2947.18 1000.63

**Per capita income**

Gamma sig+ sig- t-hat+ t-hat- CI+ CI-

----------------------------------------------------------------------

1 .010048 .010048 -593.378 -593.378 -930.358 -247.17

1.1 .005364 .017733 -641.59 -557.827 -967.398 -161.893

1.2 .002859 .028397 -686.901 -538.118 -985.987 -94.7115

1.3 .001522 .042187 -718.378 -502.567 -1017.13 -30.3497

1.4 .000809 .059079 -757.979 -430.655 -1057.03 25.1229

1.5 .00043 .078907 -792.588 -417.185 -1101.79 53.0214

1.6 .000229 .101405 -816.982 -386.901 -1134.15 139.158

1.7 .000122 .12624 -834.15 -341.018 -1159.11 186.963

1.8 .000065 .153046 -858.329 -310.73 -1177.46 213.107

1.9 .000034 .181443 -883.695 -269.035 -1213.01 253.173

2 .000018 .211061 -906.822 -253.483 -1231 308.586

**Household Consumption**

Gamma sig+ sig- t-hat+ t-hat- CI+ CI-

----------------------------------------------------------------------

1 .344559 .344559 -159.381 -159.381 -598.381 440.455

1.1 .266074 .430035 -200.891 -51.1842 -673.931 547.097

1.2 .202967 .511024 -313.881 22.4667 -798.072 601.419

1.3 .153328 .585219 -324.485 85.9625 -829.024 618.815

1.4 .114927 .651465 -351.185 116.95 -942.231 667.097

1.5 .085599 .709443 -414.553 150.175 -973.218 734.65

1.6 .063425 .75939 -454.714 169.286 -1019.29 763.3

1.7 .046794 .801874 -485.667 236.603 -1088.99 790.238

1.8 .034401 .83764 -519.667 310.119 -1107.1 878.809

1.9 .025215 .867497 -529.611 377.034 -1151.11 953.309

2 .018435 .892245 -553.238 418.154 -1193.8 1032.36

**Per capita consumption**

Gamma sig+ sig- t-hat+ t-hat- CI+ CI-

----------------------------------------------------------------------

1 .110895 .110895 -89.3121 -89.3121 -226.663 44.0297

1.1 .073894 .15891 -113.865 -75.8862 -247.479 69.917

1.2 .048839 .212577 -122.453 -69.2222 -282.081 99.7084

1.3 .03208 .269757 -137.511 -58.423 -294.331 125.429

1.4 .02097 .328483 -165.394 -37.3621 -302.454 168.488

1.5 .013656 .387089 -177.602 -27.9027 -315.52 174.038

1.6 .008866 .444257 -192.056 -17.4362 -324.49 216.395

1.7 .005742 .499008 -198.875 -3.87757 -332.508 223.571

1.8 .003712 .550661 -203.018 8.78423 -336.689 262.5

1.9 .002396 .598793 -212.269 14.3574 -349.497 300.211

2 .001544 .643183 -220.459 33.3761 -366.546 317.116

Table O: Sensitivity analysis - Rosenbaum bounds results for participation in oil palm production [Oil palm outgrowers (KW_OG) and Control (KW_C) in Kwae]

**Total Household income**

Gamma sig+ sig- t-hat+ t-hat- CI+ CI-

----------------------------------------------------------------------

1 .01343 .01343 -1808.89 -1808.89 -2844.73 -772.679

1.1 .008235 .020987 -1938.24 -1755 -2961.32 -490.985

1.2 .005059 .030484 -2102.23 -1617 -3040.23 -295.485

1.3 .003113 .041849 -2164 -1556.31 -3133.28 -90.9848

1.4 .001919 .054947 -2195.28 -1508.11 -3274.56 267.904

1.5 .001184 .069605 -2281.62 -1408.81 -3385.81 376.909

1.6 .000732 .085632 -2359.5 -1336.43 -3414.68 469.959

1.7 .000453 .102834 -2374.39 -1232.36 -3525.46 955.19

1.8 .000281 .121018 -2555 -1140.93 -3618.51 1150.69

1.9 .000174 .140001 -2593.07 -1139.86 -3642.81 1355.19

2 .000108 .159614 -2619.74 -1069.27 -3702.32 1518.94

**Per capita income**

Gamma sig+ sig- t-hat+ t-hat- CI+ CI-

----------------------------------------------------------------------

1 .006293 .006293 -909.595 -909.595 -1454.19 -307.118

1.1 .003677 .010284 -972.682 -838.811 -1542.61 -241.146

1.2 .002154 .015518 -1011.03 -791.982 -1612.14 -190.078

1.3 .001265 .022017 -1093.43 -754.398 -1672.68 -167.963

1.4 .000744 .029756 -1109.16 -738.75 -1715.86 -79.5618

1.5 .000439 .038675 -1202.1 -648.327 -1740.83 -58.1663

1.6 .000259 .04869 -1207.95 -621.727 -1767.68 -18.6521

1.7 .000153 .059704 -1239.79 -595.604 -1828.23 28.4143

1.8 .000091 .071612 -1244.34 -541.999 -1841.67 43.8542

1.9 .000054 .084307 -1304.88 -471.008 -1847.41 164.38

2 .000032 .097685 -1356.06 -444.7 -1866.76 177.725

2.1 .000019 .111645 -1378.21 -424.596 -1879.25 196.456

2.2 .000011 .126093 -1393.3 -390.112 -1907.95 373.448

2.3 6.7e-06 .140941 -1393.65 -358.037 -1916.65 382.872

2.4 4.0e-06 .156109 -1441.89 -322.558 -1939.79 414.948

2.5 2.4e-06 .171524 -1454.19 -307.118 -1942.5 430.388

2.6 1.4e-06 .187119 -1473.72 -275.285 -1980.08 433.704

2.7 8.5e-07 .202834 -1517.7 -269.612 -1980.08 475.486

2.8 5.0e-07 .218617 -1534.89 -241.146 -1996.38 532.183

2.9 3.0e-07 .234421 -1574.56 -209.07 -2015.73 564.259

3 1.8e-07 .250204 -1597.43 -209.07 -2017.67 630.081

**Household Consumption**

Gamma sig+ sig- t-hat+ t-hat- CI+ CI-

----------------------------------------------------------------------

1 .451954 .451954 36.3115 36.3115 -587.716 639.84

1.1 .524476 .3809 -40.4909 74.0341 -656.258 747.525

1.2 .590085 .319099 -78.5329 112.043 -752.024 801.801

1.3 .648466 .266078 -130.2 169.8 -789.847 823.326

1.4 .69978 .221052 -165.524 249.686 -909.743 937.468

1.5 .744458 .183108 -236.608 288.439 -956.258 993.905

1.6 .783078 .151322 -279.116 317.826 -975.225 1025.46

1.7 .816269 .124817 -325.725 359.743 -1070.78 1085.16

1.8 .844666 .102796 -371.919 390.764 -1074.64 1140.78

1.9 .868873 .084554 -389.173 469.8 -1099.69 1191.68

2 .889446 .069478 -408.847 491.923 -1121.25 1233.06

**Per capita consumption**

Gamma sig+ sig- t-hat+ t-hat- CI+ CI-

----------------------------------------------------------------------

1 .404602 .404602 -14.9864 -14.9864 -143.683 128.465

1.1 .335789 .476288 -32.6745 -4.5511 -160.723 142.014

1.2 .27711 .542421 -35.4109 10.1758 -176.526 163.807

1.3 .227688 .602343 -55.5563 19.2861 -180.671 179.023

1.4 .186442 .655911 -66.6616 26.3103 -195.628 199.153

1.5 .152257 .703307 -71.7183 48.1134 -203.507 206.833

1.6 .124074 .744909 -81.5541 58.8456 -218.613 220.034

1.7 .100935 .781196 -87.3721 62.3127 -228.72 239.993

1.8 .081998 .812688 -100.656 67.912 -235.509 250.698

1.9 .06654 .839908 -107.502 78.6175 -239.494 253.101

2 .053946 .863359 -108.777 84.2779 -244.533 271.995

2.1 .043704 .883509 -115.556 95.389 -260.941 273.255

2.2 .035386 .900782 -127.631 108.581 -269.965 282.7

2.3 .028636 .915564 -129.135 111.11 -271.834 284.7

2.4 .023165 .928193 -140.593 121.732 -287.637 307.071

2.5 .018733 .938968 -143.683 128.465 -296.478 314.095

2.6 .015145 .948153 -145.77 131.991 -297.23 317.777

2.7 .012242 .955975 -155.239 140.834 -301.781 320.263

2.8 .009893 .96263 -158.56 141.836 -306.739 330.968

2.9 .007994 .968289 -161.474 145.932 -321.911 346.097

3 .006459 .973098 -168.43 154.848 -322.662 348.097

Table P: Sensitivity analysis - Rosenbaum bounds results for participation in oil palm production [Oil palm independent smallholders (KW_ID) and Control (KW_C) in Kwae]

**Total household income**

Gamma sig+ sig- t-hat+ t-hat- CI+ CI-

----------------------------------------------------------------------

1 .156475 .156475 -810.901 -810.901 -2311.04 875.121

1.1 .109974 .213678 -925.961 -664.091 -2485.76 1101.51

1.2 .076681 .274674 -1186.76 -488.516 -2579.48 1245.76

1.3 .053143 .337107 -1445.55 -295.897 -2788.4 1415.92

1.4 .036656 .399037 -1569.16 -206.124 -2984.08 1586.45

1.5 .02519 .458987 -1672.85 -114.353 -3049.04 1718.75

1.6 .017259 .515908 -1798.48 29.1048 -3230.58 1890.22

1.7 .011797 .569118 -1866.49 146.596 -3352.19 2302.51

1.8 .008048 .618232 -1982.11 385.802 -3509.85 2626.33

1.9 .005482 .663095 -2079.37 607.557 -3567.65 2765.04

2 .003729 .70372 -2185.62 714.099 -3685.35 2891.81

**Per capita income**

Gamma sig+ sig- t-hat+ t-hat- CI+ CI-

----------------------------------------------------------------------

1 .145233 .145233 -258.53 -258.53 -724.34 243.386

1.1 .101195 .199942 -328.295 -212.161 -785.497 362.454

1.2 .069971 .258821 -373.526 -172.052 -833.796 425.65

1.3 .048098 .319593 -407.677 -127.531 -908.032 514.288

1.4 .032912 .380346 -440.921 -93.0545 -935.526 633.334

1.5 .02244 .439583 -485.93 -47.1326 -972.985 677.092

1.6 .015257 .496211 -516.507 -14.9223 -1036.94 779.089

1.7 .01035 .549492 -553.966 56.6148 -1083.82 859.654

1.8 .007008 .598977 -573.747 102.756 -1120.09 923.103

1.9 .004738 .64445 -638.48 144.453 -1173.82 940.696

2 .003199 .685866 -671.522 219.032 -1217.64 1001.69

**Total Household consumption**

Gamma sig+ sig- t-hat+ t-hat- CI+ CI-

----------------------------------------------------------------------

1 .03973 .03973 -706.491 -706.491 -1303.9 -21.9394

1.1 .024288 .06186 -792.194 -625.639 -1398.81 40.6072

1.2 .014792 .089053 -858.466 -504.408 -1508.25 117.777

1.3 .008983 .120686 -915.885 -464.725 -1575.58 186.367

1.4 .005445 .155965 -972.703 -380.08 -1643.01 272.256

1.5 .003295 .194036 -1030.81 -334.588 -1674.13 367.586

1.6 .001992 .234058 -1068.24 -280.135 -1736.07 471.383

1.7 .001203 .275249 -1096.77 -221.91 -1769.68 548.745

1.8 .000727 .316919 -1165.56 -174.33 -1823.84 616.183

1.9 .000438 .358476 -1187.22 -120.489 -1855.43 683.732

2 .000265 .39943 -1255.46 -92.403 -1909.59 760.044

**Per capita Consumption**

Gamma sig+ sig- t-hat+ t-hat- CI+ CI-

----------------------------------------------------------------------

1 .035783 .035783 -210.489 -210.489 -397.892 -17.2162

1.1 .021671 .056205 -226.976 -190.953 -417.931 15.7685

1.2 .013078 .081529 -244.139 -164.011 -445.153 61.4632

1.3 .007873 .111227 -259.539 -139.908 -464.428 93.7044

1.4 .00473 .144592 -276.691 -129.218 -476.913 117.839

1.5 .002838 .180842 -297.589 -114.957 -494.065 147.185

1.6 .001701 .21919 -313.254 -78.728 -511.217 180.93

1.7 .001019 .258892 -334.151 -58.5767 -530.882 195.775

1.8 .00061 .29928 -351.894 -45.8488 -540.524 201.828

1.9 .000365 .339774 -359.46 -36.777 -553.499 219.197

2 .000219 .379883 -374.513 -28.2562 -596.619 246.017

**Notes for Tables L-P**

gamma - log odds of differential assignment due to unobserved factors

sig+ - upper bound significance level

sig- - lower bound significance level

t-hat+ - upper bound Hodges-Lehmann point estimate

t-hat- - lower bound Hodges-Lehmann point estimate

CI+ - upper bound confidence interval (a= .9)

CI- - lower bound confidence interval (a= .9)

Table Q: Correlations between income, consumption and subjective wellbeing indicators for sugarcane smallholders in Dabala

|  | **Mean adult consumption equivalent (GH¢)** | **Mean annual income per household (GH¢)** | **Satisfaction with life** | **Worthwhileness** | **Happiness** | **Anxiousness** |
| --- | --- | --- | --- | --- | --- | --- |
| **Mean adult consumption equivalent (GH¢)** | 1 |  |  |  |  |  |
| **Mean annual income per household (GH¢)** | 0.468^**^ | 1 |  |  |  |  |
| **Satisfaction with life** | -0.115 | -0.230^*^ | 1 |  |  |  |
| **Worthwhileness** | -0.054 | -0.008 | 0.111 | 1 |  |  |
| **Happiness** | 0.003 | -0.084 | 0.057 | 0.212^*^ | 1 |  |
| **Anxiousness** | -0.076 | 0.104 | -0.013 | .024 | -0.052 | 1 |

** Correlation is significant at the 0.01 level, * Correlation is significant at the 0.05 level (2 tailed test)

Table R: Correlations between income, consumption and subjective wellbeing indicators for control group in Dabala

|  | **Mean adult consumption equivalent (GH¢)** | **Mean annual income per household (GH¢)** | **Satisfaction with life** | **Worthwhileness** | **Happiness** | **Anxiousness** |
| --- | --- | --- | --- | --- | --- | --- |
| **Mean adult consumption equivalent (GH¢)** | 1 |  |  |  |  |  |
| **Mean annual income per household (GH¢)** | 0.432^**^ | 1 |  |  |  |  |
| **Satisfaction with life** | 0.292^**^ | 0.042 | 1 |  |  |  |
| **Worthwhileness** | 0.133 | 0.008 | 0.152 | 1 |  |  |
| **Happiness** | 0.130 | 0.072 | 0.185 | 0.017 | 1 |  |
| **Anxiousness** | -0.113 | 0.049 | -0.145 | -0.079 | -0.068 | 1 |

** Correlation is significant at the 0.01 level, * Correlation is significant at the 0.05 level (2 tailed test)

Table S: Correlations between income, consumption and subjective wellbeing indicators for permanent jatropha workers in Yeji

|  | **Mean adult consumption equivalent (GH¢)** | **Mean annual income per household (GH¢)** | **Satisfaction with life** | **Worthwhileness** | **Happiness** | **Anxiousness** |
| --- | --- | --- | --- | --- | --- | --- |
| **Mean adult consumption equivalent (GH¢)** | 1 |  |  |  |  |  |
| **Mean annual income per household (GH¢)** | 0.225^*^ | 1 |  |  |  |  |
| **Satisfaction with life** | 0.236^*^ | 0.163 | 1 |  |  |  |
| **Worthwhileness** | 0.160 | 0.121 | 0.647^**^ | 1 |  |  |
| **Happiness** | 0.233^*^ | 0.115 | 0.760^*^ | 0.538^**^ | 1 |  |
| **Anxiousness** | -0.001 | 0.090 | 0.271^**^ | 0.504^**^ | 0.245^*^ | 1 |

** Correlation is significant at the 0.01 level, * Correlation is significant at the 0.05 level (2 tailed test)

Table T: Correlations between income, consumption and subjective wellbeing indicators for seasonal jatropha seasonal workers in Yeji

|  | **Mean adult consumption equivalent (GH¢)** | **Mean annual income per household (GH¢)** | **Satisfaction with life** | **Worthwhileness** | **Happiness** | **Anxiousness** |
| --- | --- | --- | --- | --- | --- | --- |
| **Mean adult consumption equivalent (GH¢)** | 1 |  |  |  |  |  |
| **Mean annual income per household (GH¢)** | 0.492^**^ | 1 |  |  |  |  |
| **Satisfaction with life** | 0.093 | 0.025 | 1 |  |  |  |
| **Worthwhileness** | -0.116 | -0.151 | 0.407^**^ | 1 |  |  |
| **Happiness** | -0.042 | 0.054 | 0.542^**^ | 0.667^**^ | 1 |  |
| **Anxiousness** | -0.239 | -0.182 | -0.151 | 0.508^**^ | 0.419^**^ | 1 |

** Correlation is significant at the 0.01 level, * Correlation is significant at the 0.05 level (2 tailed test)

Table U: Correlations between income, consumption and subjective wellbeing indicators for the control group in Yeji

|  | **Mean adult consumption equivalent (GH¢)** | **Mean annual income per household (GH¢)** | **Satisfaction with life** | **Worthwhileness** | **Happiness** | **Anxiousness** |
| --- | --- | --- | --- | --- | --- | --- |
| **Mean adult consumption equivalent (GH¢)** | 1 |  |  |  |  |  |
| **Mean annual income per household (GH¢)** | 0.412^**^ | 1 |  |  |  |  |
| **Satisfaction with life** | 0.127 | 0.184 | 1 |  |  |  |
| **Worthwhileness** | 0.119 | 0.256^*^ | 0.517^**^ | 1 |  |  |
| **Happiness** | 0.306^**^ | 0.239^*^ | 0.565^**^ | 0.573^**^ | 1 |  |
| **Anxiousness** | -0.043 | 0.151 | 0.020 | 0.499^**^ | 0.292^**^ | 1 |

** Correlation is significant at the 0.01 level, * Correlation is significant at the 0.05 level (2 tailed test)

Table V: Correlations between income, consumption and subjective wellbeing indicators for GOPDC workers in Kwae

|  | **Mean adult consumption equivalent (GH¢)** | **Mean annual income per household (GH¢)** | **Satisfaction with life** | **Worthwhileness** | **Happiness** | **Anxiousness** |
| --- | --- | --- | --- | --- | --- | --- |
| **Mean adult consumption equivalent (GH¢)** | 1 |  |  |  |  |  |
| **Mean annual income per household (GH¢)** | 0.520^**^ | 1 |  |  |  |  |
| **Satisfaction with life** | -0.139 | -0.106 | 1 |  |  |  |
| **Worthwhileness** | 0.084 | -0.107 | -0.223^*^ | 1 |  |  |
| **Happiness** | 0.049 | 0.306^**^ | 0.125 | 0.105 | 1 |  |
| **Anxiousness** | -0.177 | -0.084 | -0.182 | 0.320^**^ | 0.129 | 1 |

** Correlation is significant at the 0.01 level, * Correlation is significant at the 0.05 level (2 tailed test)

Table W: Correlations between income, consumption and subjective wellbeing indicators for oil palm outgrowers in Kwae

|  | **Mean adult consumption equivalent (GH¢)** | **Mean annual income per household (GH¢)** | **Satisfaction with life** | **Worthwhileness** | **Happiness** | **Anxiousness** |
| --- | --- | --- | --- | --- | --- | --- |
| **Mean adult consumption equivalent (GH¢)** | 1 |  |  |  |  |  |
| **Mean annual income per household (GH¢)** | 0.088 | 1 |  |  |  |  |
| **Satisfaction with life** | 0.038 | -0.061 | 1 |  |  |  |
| **Worthwhileness** | 0.059 | -0.100 | 0.515^**^ | 1 |  |  |
| **Happiness** | 0.077 | -0.144 | 0.737^**^ | 0.457^**^ | 1 |  |
| **Anxiousness** | 0.009 | -0.140 | 0.184 | 0.214^*^ | 0.245^*^ | 1 |

** Correlation is significant at the 0.01 level, * Correlation is significant at the 0.05 level (2 tailed test)

Table Z: Correlations between income, consumption and subjective wellbeing indicators for independent oil palm smallholders in Kwae

|  | **Mean adult consumption equivalent (GH¢)** | **Mean annual income per household (GH¢)** | **Satisfaction with life** | **Worthwhileness** | **Happiness** | **Anxiousness** |
| --- | --- | --- | --- | --- | --- | --- |
| **Mean adult consumption equivalent (GH¢)** | 1 |  |  |  |  |  |
| **Mean annual income per household (GH¢)** | 0.642^**^ | 1 |  |  |  |  |
| **Satisfaction with life** | 0.076 | 0.221^*^ | 1 |  |  |  |
| **Worthwhileness** | 0.249^*^ | 0.226^*^ | 0.207 | 1 |  |  |
| **Happiness** | 0.004 | 0.205 | 0.567^**^ | 0.196^*^ | 1 |  |
| **Anxiousness** | -0.017 | -0.076 | -0.267^**^ | 0.416^**^ | -0.251^*^ | 1 |

** Correlation is significant at the 0.01 level, * Correlation is significant at the 0.05 level (2 tailed test)

Table Y: Correlations between income, consumption and subjective wellbeing indicators for control group in Kwae

|  | **Mean adult consumption equivalent (GH¢)** | **Mean annual income per household (GH¢)** | **Satisfaction with life** | **Worthwhileness** | **Happiness** | **Anxiousness** |
| --- | --- | --- | --- | --- | --- | --- |
| Mean adult consumption equivalent (GH¢) | 1 |  |  |  |  |  |
| Total income (GH¢) | 0.506^**^ | 1 |  |  |  |  |
| Satisfaction with life | -0.059 | -0.147 | 1 |  |  |  |
| Worthwhileness | 0.024 | -0.027 | 0.534^**^ | 1 |  |  |
| Happiness | -0.036 | 0.003 | 0.442^**^ | 0.315^**^ | 1 |  |
| Anxiety | -0.100 | -0.184 | 0.573^**^ | 0.379^**^ | 0.398^**^ | 1 |

** Correlation is significant at the 0.01 level, * Correlation is significant at the 0.05 level (2 tailed test)
